# Dynamic rewiring of the human interactome by interferon signaling

**DOI:** 10.1186/s13059-020-02050-y

**Published:** 2020-06-15

**Authors:** Craig H. Kerr, Michael A. Skinnider, Daniel D. T. Andrews, Angel M. Madero, Queenie W. T. Chan, R. Greg Stacey, Nikolay Stoynov, Eric Jan, Leonard J. Foster

**Affiliations:** 1 Michael Smith Laboratories, University of British Columbia, Vancouver, BC V6T 1Z4 Canada; 2 Department of Biochemistry and Molecular Biology, University of British Columbia, Vancouver, BC V6T 1Z3 Canada; 3 Current Address: Department of Genetics, Stanford University, Stanford, CA 94305 USA

**Keywords:** Interferon, Proteomics, Interferon-stimulated gene, Innate immunity, Protein correlation profiling, Interactome, Protein complexes

## Abstract

**Background:**

The type I interferon (IFN) response is an ancient pathway that protects cells against viral pathogens by inducing the transcription of hundreds of IFN-stimulated genes. Comprehensive catalogs of IFN-stimulated genes have been established across species and cell types by transcriptomic and biochemical approaches, but their antiviral mechanisms remain incompletely characterized. Here, we apply a combination of quantitative proteomic approaches to describe the effects of IFN signaling on the human proteome, and apply protein correlation profiling to map IFN-induced rearrangements in the human protein-protein interaction network.

**Results:**

We identify > 26,000 protein interactions in IFN-stimulated and unstimulated cells, many of which involve proteins associated with human disease and are observed exclusively within the IFN-stimulated network. Differential network analysis reveals interaction rewiring across a surprisingly broad spectrum of cellular pathways in the antiviral response. We identify IFN-dependent protein-protein interactions mediating novel regulatory mechanisms at the transcriptional and translational levels, with one such interaction modulating the transcriptional activity of STAT1. Moreover, we reveal IFN-dependent changes in ribosomal composition that act to buffer IFN-stimulated gene protein synthesis.

**Conclusions:**

Our map of the IFN interactome provides a global view of the complex cellular networks activated during the antiviral response, placing IFN-stimulated genes in a functional context, and serves as a framework to understand how these networks are dysregulated in autoimmune or inflammatory disease.

## Background

Type I interferons (IFNs) are an evolutionary ancient family of cytokines that play a central role in the immune response to viral pathogens [1]. IFN synthesis and secretion are triggered in response to pathogen detection by intra- and extracellular receptors, leading to the activation of multiple defense mechanisms via the transcription of IFN-stimulated genes (ISGs) [2]. These ISGs contribute to the establishment of a cell-intrinsic antiviral state in infected and neighboring cells, while also modulating the development of innate and adaptive immune responses [3]. Activation of the IFN response must be carefully regulated in order to strike a balance between effective pathogen clearance on the one hand and tissue damage or auto-inflammatory pathology on the other, as aberrant IFN signaling has been implicated in a range of autoimmune and neuropsychiatric diseases [4, 5].

In the canonical type I IFN signaling pathway, IFNs bind the heterodimeric IFNɑ receptor (IFNAR) complex, thereby activating the receptor-associated tyrosine kinases JAK1 and TYK2. In turn, these kinases phosphorylate the cytoplasmic STAT1 and STAT2 transcription factors. Translocation of STAT1 and STAT2 to the nucleus, followed by association with IRF9 to form the IFN-stimulated gene factor 3 (ISGF3) complex, activates ISG transcription. While some of these ISGs encode proteins with direct antiviral activity, many ISG products modulate parallel signaling pathways or encode additional transcription factors. Consequently, IFN stimulation induces a complex response that is not limited to a simple antiviral program, but instead activates a number of additional signaling pathways such as the MAPK cascade and the mTOR-AKT-S6K axis, which contribute to ISG induction or the antiviral response more broadly [3]. Ultimately, this cascade results in substantial remodeling of mRNA processing, post-translational modifications, metabolism, cellular trafficking, chromatin organization, and the cytoskeleton, among other processes [6].

A combination of unbiased transcriptome profiling [2, 7–9] and biochemical approaches [10–13] has identified hundreds of ISGs and, in some cases, elucidated their mechanism of action. Yet the functional roles of most ISGs as effectors of the innate immune response remain to be fully characterized. Furthermore, in view of the limited ability of mRNA levels to predict cellular protein abundance [14, 15], the degree to which IFN-induced changes in transcriptional activity ultimately manifest at the level of the proteome remains incompletely understood. A complete understanding of the IFN signaling repertoire would include a direct interrogation of the complex network of interacting proteins that mediate the type I IFN response, beyond those with a direct role in restricting viral replication. However, experimentally mapping the cellular interaction network in differential and physiologically relevant contexts at the proteome scale represents a long-standing challenge [16].

Here, we apply a combination of quantitative proteomic approaches to chart the molecular landscape of type I IFN signaling, culminating in the use of protein correlation profiling (PCP) [17] to map interferon-induced rearrangements in the human interactome. The resulting protein-protein interaction network, encompassing over 26,000 interactions, reveals widespread rewiring of physical interactions and places known ISGs in an IFN-dependent functional context. We find evidence that an evolutionarily conserved subset of ISGs are induced to physically interact in response to IFN-β stimulation, and experimentally validate the role of one such interaction in modulating STAT1-mediated transcription. We develop statistical methods for differential network analysis to characterize interactome rewiring at the functional level, leading us to identify alterations in ribosome composition induced by interferon signaling that selectively downregulate ISG synthesis in order to fine-tune the IFN response. Collectively, this differential network map of the IFN-induced interactome provides a resource to mechanistically dissect the IFN response in the context of viral infection and autoimmune disease.

## Results

### Proteome-wide analysis of the type I IFN response

**Fig. 1:**
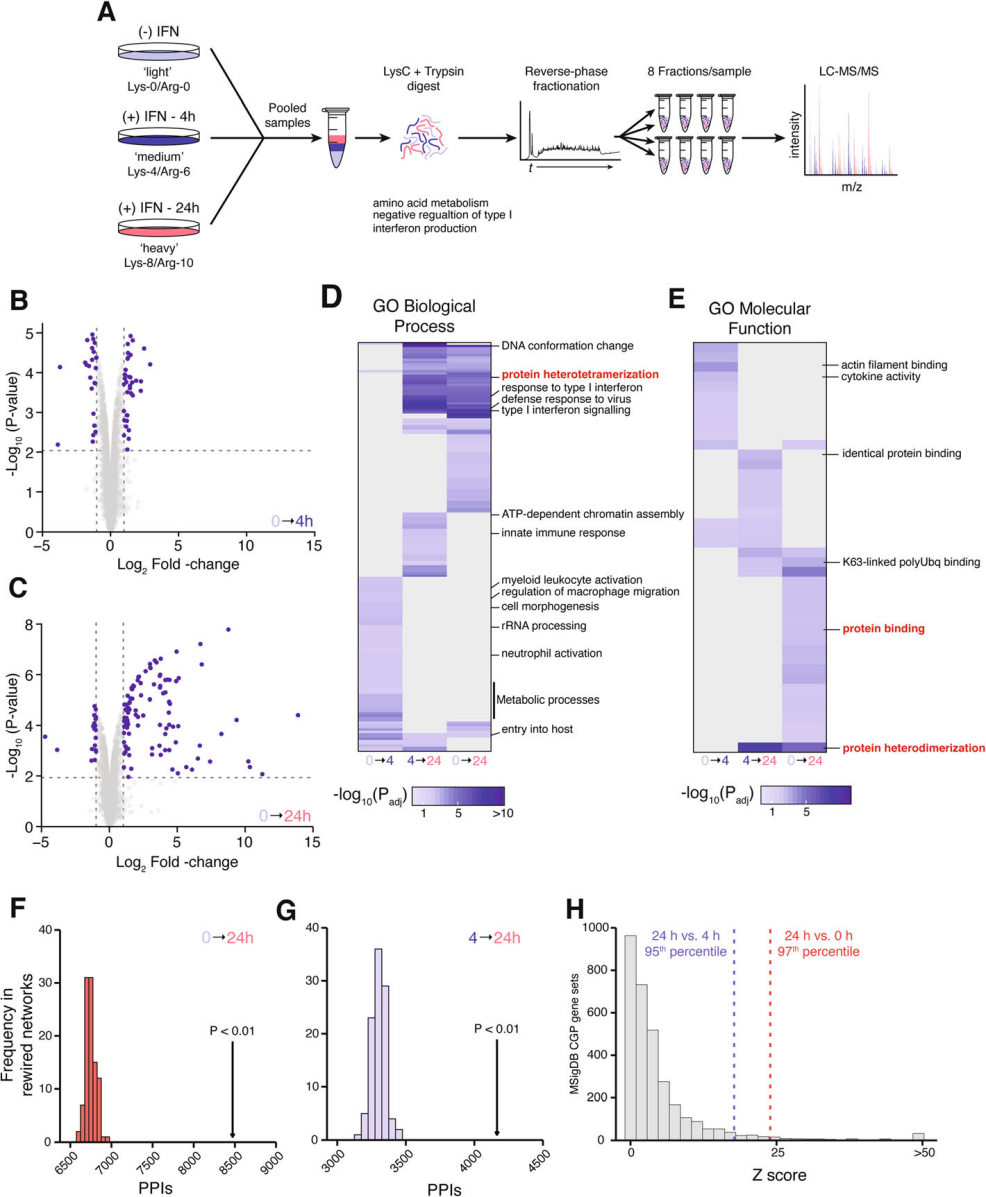
Quantitative proteomic analysis of IFNβ stimulation. **a** Schematic overview of the shotgun proteomics workflow for analysis of IFN-induced proteome changes. **b**, **c** Volcano plot showing differential protein abundance in cells stimulated with IFNβ for 4 h (**b**) or 24 h (**c**). Vertical lines denote absolute fold change ≥ 2. Horizontal lines show 5% FDR threshold. **d**, **e** Gene Ontology (GO) terms for biological processes (**d**) and molecular functions (**e**) significantly enriched among differentially expressed proteins after 4 h or 24 h of IFNβ stimulation. **f**, **g** Number of protein-protein interactions in the InBioMap database [19] between differentially expressed proteins after 24 h (**f**) of IFNβ stimulation, or between cells stimulated for 4 and 24 h (**f**), arrows, and in 100 randomly rewired networks derived from the same database, histograms. **h** Enrichment for protein-protein interactions between 3263 curated gene signatures from the MSigDB chemical and genetic perturbations collection, histogram, and the sets of proteins differentially expressed after 24 h of IFN stimulation relative to unstimulated cells or cells stimulated with IFN for 4 h, dotted lines

Whereas the transcriptional response to IFN stimulation has been extensively characterized, the dynamic changes occurring at the proteome level remain unclear. In view of the multiple biological mechanisms that exist to decouple protein abundance from mRNA expression [18], we therefore first sought to establish the proteome-wide response to IFN-β (hereafter, “IFN”) stimulation. We applied stable isotopic labeling by amino acids in cell culture (SILAC)-based mass spectrometry to precisely quantify protein abundance in HeLa cells after 4 h or 24 h of IFN stimulation (Fig. 1a). A total of 7421 proteins were identified, of which 5016 were quantified in all three replicates (Additional file 2). After 4 h of IFN stimulation, a timepoint by which most ISGs have reached their maximal mRNA expression [8], we detected 924 differentially expressed proteins at a 5% FDR, but only 36 with greater than twofold induction (Fig. 1b). Conversely, after 24 h of IFN stimulation, we observed more pronounced changes in the cellular proteome, with 1172 proteins differentially expressed at 5% FDR and 105 with at least a twofold induction (Fig. 1c, Additional file 1: Fig. S1A). Several proteins with well-appreciated roles in the type I IFN response were induced over 100-fold, including the IFIT proteins (IFIT1, IFIT2, and IFIT3), MX1, and ISG15 (Additional file 2).

Functional enrichment analysis of the proteins that were differentially expressed at 4 h or 24 h, or which were differentially expressed between the two timepoints, revealed marked temporal differences in the cellular processes activated by IFN stimulation (Fig. 1d, e, Additional file 1: Fig. S1B, Additional file 3). Proteins that were differentially expressed at 4 h were enriched for Gene Ontology (GO) terms related to involvement in metabolic processes, such as “glycolipid catabolic process” and “regulation of steroid biosynthetic process,” consistent with the notion that IFN signaling may induce changes to cellular metabolism in order to establish an antiviral state [20, 21]. Other enriched GO terms pointed to a role for cell migration, including “regulation of macrophage migration” and “cell morphogenesis.” In contrast, some of the most significantly enriched GO terms at 24 h were related to chromatin rearrangements: for instance, “DNA conformational change” and “DNA-replication-dependent nucleosome assembly.” These enrichments are in line with the finding that IFN stimulation induces chromatin modifications to establish a transcriptional “memory,” resulting in faster and greater transcriptional responses upon restimulation [22]. Enrichment was also observed for processes such as “posttranscriptional gene silencing,” “gene silencing by RNA,” and “de novo protein folding,” which may reflect the changing cellular environment after IFN stimulation in preparation for host defense. Surprisingly, despite the rapid induction of ISG transcription (as early as 30 min post-stimulation [8];), we did not observe an enrichment of Gene Ontology (GO) terms related to the innate immune response until 24 h, reflecting an apparent lag in translation of canonical ISGs (Fig. 1b, c).

Our attention was drawn to the enrichment for GO terms related to protein-protein interactions after 24 h of IFN stimulation, including “protein binding,” “protein heterodimerization,” and “protein heterotetramerization.” We therefore sought to determine whether proteins that were differentially expressed at 24 h, in comparison to cells stimulated with IFN for 4 h or to unstimulated cells, displayed a statistically significant tendency to physically interact. To test this hypothesis, we compared the observed number of protein-protein interactions between differentially expressed proteins in each of the two comparisons, using the InBio Map database [19], to the number of interactions observed in networks rewired using degree-preserving randomization [23]. Importantly, unlike a null model in which an equivalent number of proteins are drawn at random from the network, this rewiring-based null model controls for artifactual differences stemming from the degree distribution of the network, whereby a statistically significant result can reflect the high connectivity of the differentially exposed proteins across the entire network as opposed to their selective interconnectivity with one another [24, 25]. This analysis revealed a substantial excess of physical protein-protein interactions between differentially expressed proteins, relative to random expectation (Fig. 1f, g). We compared the strength of this enrichment to that observed for 3263 curated gene expression signatures of disparate biological and clinical states cataloged in the MSigDB “chemical and genetic perturbations” (CGP) collection. Remarkably, proteins differentially expressed in the 24 h vs. 4 h and 24 h vs. unstimulated comparisons displayed a greater enrichment for physical interactions than 95% and 97% of CGP signatures, respectively (Fig. 1h). Together, these results led us to hypothesize that in addition to its effects on ISG transcription, IFN stimulation induces rewiring of cellular protein-protein interaction networks.

### Quantitative interactome profiling of the type I IFN response

**Fig. 2:**
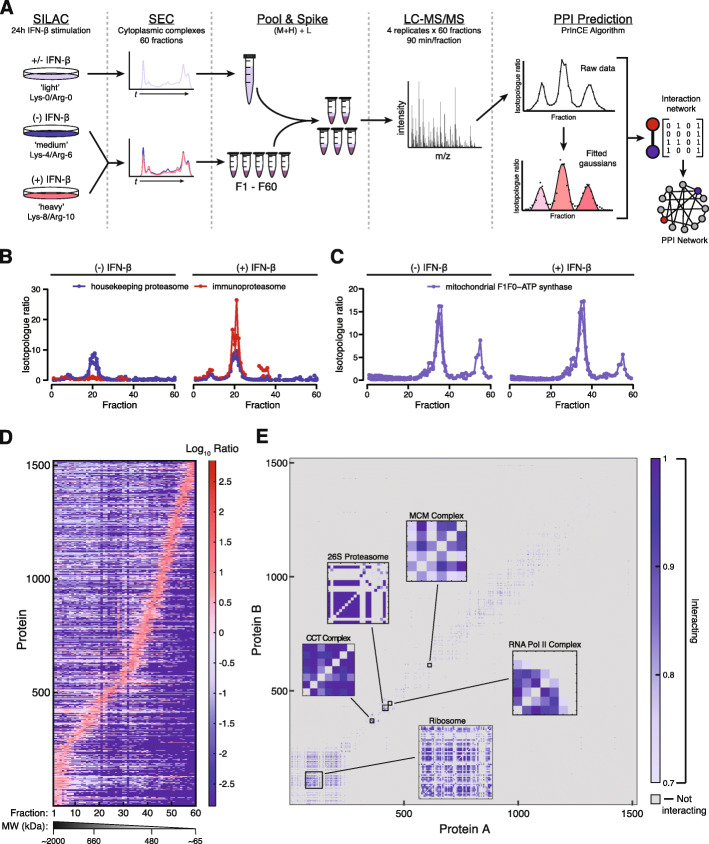
Mapping the interactome of IFN-stimulated cells by SEC-PCP-SILAC. **a** Schematic overview of the SEC-PCP-SILAC workflow. **b** PCP chromatograms of housekeeping proteasome and immunoproteasome-specific proteins in stimulated and unstimulated cells. **c** PCP chromatograms of mitochondrial F_1_F_0_–ATPase complex proteins in stimulated and unstimulated cells. **d** Complete set of PCP chromatograms passing quality control in PrInCE (*n* = 1520) defined by heavy/light ratio, arranged by index of maximum protein abundance, from a representative biological replicate. **e** Protein-protein interaction adjacency matrix for all 27,694 interactions with a precision greater than 70%, colored by interaction precision. Insets show adjacency matrices of known protein complexes from the CORUM database [28]

To map rearrangements in the human interactome induced by IFN stimulation, we applied a quantitative proteomic strategy based on protein correlation profiling (PCP) [26, 27] in combination with size exclusion chromatography (SEC). Under this workflow, protein complexes are separated by their size, and interacting protein pairs are inferred based on the similarity of their elution profiles (Fig. 2a). The use of triplex SILAC labeling (SEC-PCP-SILAC) further enables differential analysis of protein-protein interactions between stimulated and unstimulated cellular states, to a high degree of quantitative precision [17].

We applied SEC-PCP-SILAC to simultaneously compare the interactomes of cells stimulated with IFN for 24 h, labeled with heavy isotopes, and unstimulated cells, labeled with medium isotopes (Fig. 2a). Fractions from the light channel, which included both stimulated and unstimulated cells in order to maximize proteome coverage, were pooled and spiked into all fractions as an internal standard. Sixty fractions were collected from each of three biological replicates and were individually subjected to liquid chromatography–tandem mass spectrometry (LC-MS/MS) analysis. The resulting dataset was processed as a single experiment via MaxQuant [29] at a peptide and protein false discovery rate (FDR) of 1%, leading to the identification of 42,843 unique peptides from 2590 protein groups across all 180 fractions (Fig. 2d). Inspection of the PCP chromatograms revealed marked shifts between conditions for protein complexes with known roles in the innate immune response, such as the immunoproteasome (Fig. 2b). Conversely, no differences between conditions were observed for housekeeping complexes such as the mitochondrial F_1_F_0_–ATP synthase, supporting the specificity of the technique (Fig. 2c).

### Reconstruction of a high-confidence interactome network

To recover a high-confidence network of protein-protein interactions, we developed a multi-stage bioinformatic pipeline. First, whereas discussion of error rates in quantitative proteomics to date has focused primarily on errors in protein identification [30, 31], we observed a number of apparent errors in protein quantitation, some of which resulted in high-magnitude deviations in protein chromatograms (Additional file 1: Fig. S2C). Errors of this type have the potential to interfere with interaction detection or to introduce spurious differential interactions between conditions. We therefore developed a network-based algorithm, MODERN, to remove erroneous protein quantitations prior to further analysis (see the “Methods” section). Application of MODERN to all three replicates led to removal of 1308 erroneous protein quantitations (of 245,841 total quantitations, or 0.53%; Additional file 1: Figs. S2A-B).

Next, to infer a network of protein-protein interactions from the resulting chromatogram matrices, we applied PrInCE, a machine-learning pipeline for analysis of co-fractionation data [32, 33]. PrInCE fits a mixture of Gaussians to each chromatogram, then calculates a series of six features for each protein pair that reflect the likelihood of a physical interaction between those two proteins (see the “Methods” section). These features are provided as input to a naive Bayes classifier, which calculates an interaction probability for every pair. Importantly, PrInCE assigns the likelihood of putative interactions based solely on the chromatograms themselves, without incorporating additional evidence from published functional genomics datasets, in contrast to several other approaches [34–37]. This approach facilitates unbiased detection of novel protein-protein interactions, without sacrificing discriminative power [38].

**Fig. 3:**
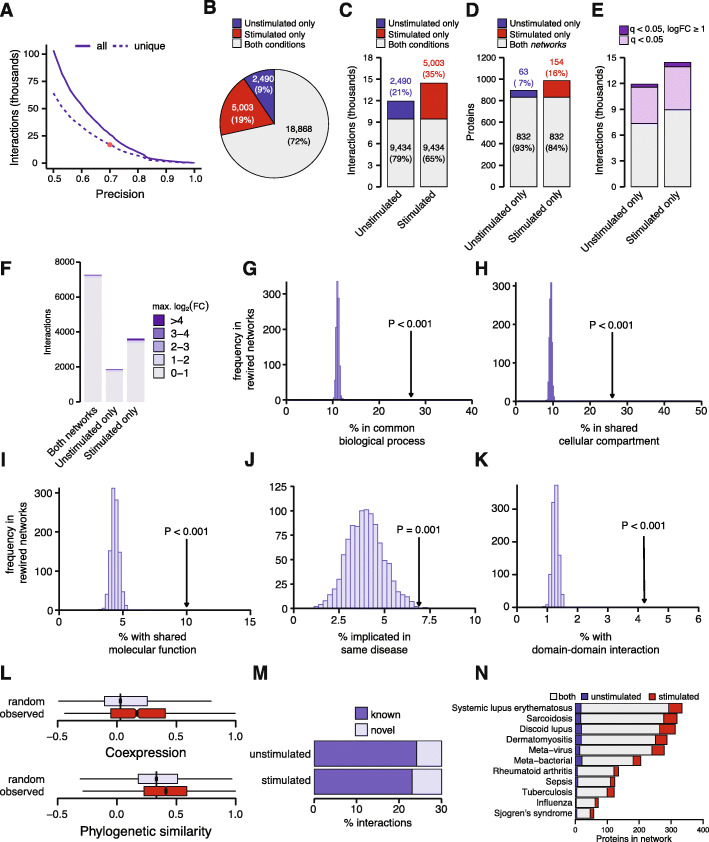
Biological relevance of the IFN interactome. **a** Precision-recall curve of all protein-protein interactions from unstimulated and IFN-stimulated networks (solid line), or unique interactions only (dashed line). **b** Proportion of all detected interactions observed in both networks or in one condition only. **c** Number of interactions in the unstimulated and IFN-stimulated network detected in both networks or in one condition only. **d** Number of proteins for which at least one interaction was detected in the unstimulated and IFN-stimulated network or in one condition only. **e** Number of interactions in the unstimulated and IFN-stimulated network involving a protein for which a statistically significant change in abundance was detected at 24 h by SILAC shotgun proteomics at 5% FDR, and the subset of these for which a twofold or greater change in abundance was detected. **f** Maximum log_2_-fold change among interacting protein pairs detected in both networks, the unstimulated network only, or the IFN-stimulated network only. **g**–**i** Proportion of interacting protein pairs sharing at least one biological process (**g**), cellular compartment (**h**), or molecular function (**i**) Gene Ontology term in the IFN interactome, arrow, or 1000 randomly rewired networks, histogram. **j** Proportion of interacting protein pairs implicated in the same disease in the IFN interactome, arrow, or 1000 randomly rewired networks, histogram. **k** Proportion of interacting protein pairs supported by a domain-domain interaction [39] in the IFN interactome, arrow, or 1000 randomly rewired networks, histogram. **l** Pearson’s correlations reflecting protein abundance and phylogenetic profile similarity between interacting protein pairs in the IFN interactome or a randomly rewired network. **m** Proportion of previously known interactions in unstimulated and IFN-stimulated cells. **n** Number of genes found to be differentially expressed at 1% FDR in meta-analyses of gene expression in eleven infectious or autoimmune diseases that were identified in the unstimulated or IFN-stimulated networks, or both

At a precision of 70%, PrInCE recovered a total of 26,361 protein-protein interactions involving 1049 proteins (Fig. 3a, Additional file 4). Visualization of the adjacency matrix revealed a sparse network of physical interactions and confirmed recovery of well-known cellular protein complexes, such as the 26S proteasome, the chaperonin containing TCP-1 complex, and RNA polymerase II (Fig. 2e). To more systematically assess the recovery of known protein complexes, we compared the network to the CORUM database, finding 206 of 296 human protein complexes (69.6%) had at least one subunit represented in the network (Additional file 1: Fig. S3A and C, Additional file 5). This proportion rose to 81.4% (96 of 118) when considering only those complexes previously found to be amenable to detection by PCP [40] (Additional file 1: Fig. S3B and D), and was broadly similar for protein complexes of different sizes (Additional file 1: Fig. S3E-F), albeit with a modest bias towards large complexes. Among the 16,927 unique interactions detected in either condition, 4046 represented co-complex interactions within known CORUM protein complexes, corresponding to a recall of 6.9% of unique interactions in CORUM and 15.0% within the subset of complexes amenable to detection by PCP (Additional file 1: Fig. S3G).

Among the 26,361 protein-protein interactions detected overall, 11,924 were identified in unstimulated cells and 14,437 in IFN-stimulated cells, with 9434 detected under both conditions (Fig. 3c-d and Additional file 1: Fig. S4A-B). Thus, 71.6% of all detected interactions were identified in both networks. This rewiring could not be attributed solely to the variable protein composition of the two networks, as a smaller proportion of protein nodes themselves were specific to one of the two networks (Fig. 3d). To investigate the degree to which changes in protein abundance in response to IFN stimulation could underlie the observed rewiring, we overlaid our shotgun proteomics data (at the 24-h timepoint; Fig. 1c) onto the IFN-stimulated and unstimulated networks (Additional file 1: Fig. S4C-D, Additional file 4). A total of 6562 unique interactions involved at least one protein with a statistically significant difference in abundance upon IFN stimulation, but most of these proteins exhibited relatively subtle changes in expression (Fig. 3e). In contrast, fewer interactions involved proteins with more dramatic IFN-induced changes in abundance (i.e., twofold or greater). Moreover, whereas interactions involving differentially expressed proteins were equally likely to be observed in either or both of the IFN-stimulated or unstimulated networks (*p* = 0.20, *χ*^2^ test), interactions involving proteins with twofold or greater differences in abundance were significantly enriched among the set of condition-specific interactions (*p* < 10^−15^, *χ*^2^ test; Additional file 1: Fig. S4E). To further assess this trend, we calculated, for all interactions, the maximum log_2_-fold change within the interacting protein pair and confirmed that only a small fraction of interactions involve proteins with dramatic changes in abundance (Fig. 3f, Additional file 1: Fig. S4F).

Overall, these results suggest that only a small number of condition-specific interactions involve proteins with pronounced IFN-induced changes in abundance. More subtle changes in expression affect a larger number of interactions and might act to “fine-tune” interactions. However, the large number of interactions between proteins that are not differentially expressed in response to IFN stimulation suggests a role for other mechanisms, such as post-translational modification, subcellular localization, or protein degradation, in IFN-dependent interactome remodeling.

### Biological relevance of the IFN interactome

We evaluated the overall biological relevance of the IFN-induced interactome by quantifying the degree to which interacting protein pairs tend to be involved in the same biological functions, localize to the same cellular compartments, or share the same molecular activities (Fig. 3g–i). In all cases, we observed highly significant enrichments for shared GO terms between interacting pairs, relative to rewired networks (all *p* < 0.001, permutation test). Further, we found interacting protein pairs were significantly more likely than random expectation to be implicated in the same disease (*p* = 0.005, permutation test; Fig. 3j), and had more correlated patterns of protein abundance and phylogenetic profiles than non-interacting pairs (*p* < 10^−15^, Brunner–Munzel test; Fig. 3l). Finally, interacting proteins were significantly more likely to share pairs of protein domains observed to physically interact in three-dimensional structural data [39], reflecting the power of SEC-PCP-SILAC to resolve physical protein-protein interactions, and not only functional associations (*p* < 0.001, permutation test; Fig. 3k). The enrichment for known correlates of physical interaction observed in the SEC-PCP-SILAC network was broadly comparable to, although slightly lower than, literature-curated interactions from small-scale experiments compiled in the InnateDB database (Additional file 1: Fig. S5A-G) [41]. Despite this enrichment, however, comparison of the IFN interactome to literature-curated protein-protein interactions recorded in eighteen databases revealed that the majority of interactions (13,240 of 16,927, or 78.2%) detected were novel. Intriguingly, the IFN-stimulated network was modestly depleted for known interactions, relative to the unstimulated network (*p* = 0.042, *χ*^2^ test; Fig. 3m), suggesting IFN stimulation specifically induces as-of-yet unmapped protein-protein interactions. Thus, multiple orthogonal lines of evidence support the high quality of our IFN interactome map, despite its recovery independent of any existing biological information.

Given that aberrant IFN signaling has been implicated in a broad range of infectious or autoimmune diseases, we further asked whether the IFN interactome could be used to interpret existing molecular datasets relevant to human pathologies. We drew on a resource of multi-cohort gene expression meta-analyses for 103 diseases [42, 43] to identify genes with reproducible evidence of differential expression in eleven diseases characterized by an elevated IFN transcriptional signature [8], including viral infections, systemic and discoid lupus erythematosus, rheumatoid arthritis, sarcoidosis, and Sjogren’s syndrome. We mapped protein-protein interactions for dozens to hundreds of differentially expressed genes from each disease (Fig. 3n); notably, interactions for many such gene products were identified exclusively in the IFN-stimulated condition. For ten of eleven diseases, genes upregulated at a 1% FDR were significantly over-represented among interactions detected only after IFN stimulation (Additional file 1: Fig. S5H). Moreover, for all eleven diseases, these genes were significantly enriched among IFN-specific interactions compared to literature-curated interactions compiled from eighteen databases (Additional file 1 : Fig. S5H). Thus, the IFN interactome provides a reference to understand the consequences of dysregulated IFN signaling in a diverse range of human pathologies, by placing transcriptional markers of auto-inflammatory disease into a functional context.

### Evolutionary plasticity of the IFN response is mirrored at the interactome level

Comparative genomics approaches have highlighted genes involved in pathogen defense and the innate immune response as rapidly evolving, with divergence in both coding and regulatory sequences across species [44–46]. However, it remains unclear how this evolutionary divergence at the sequence level ultimately manifests at the interactome level. We quantified the degree to which each protein is “rewired” upon IFN stimulation within the human interactome by calculating its autocorrelation between stimulated and unstimulated networks [47] (see the “Methods” section). The tier of proteins with the lowest autocorrelation scores included several proteins with well-established roles in the innate immune response, such as the IFIT proteins and components of the immunoproteasome (Additional file 1: Fig. S6A, Additional file 6). Comparing the IFN-induced autocorrelation of each protein to its evolutionary rate, as quantified by the ratio of non-synonymous to synonymous substitutions (dN/dS), revealed a modest, but statistically significant negative correlation (Spearman’s *ρ* = − 0.11, *p* = 1.9 × 10^−4^; Additional file 1: Fig. S6B). Similarly modest but significant associations were observed in comparisons to the total number of species in which an ortholog of a given gene was present (*ρ* = 0.14, *p* = 5.3 × 10^−7^; Additional file 1: Fig. S6C) [48], or to the pLI score [49], a measure of mutational constraint derived from large-scale human exome sequencing (*ρ* = 0.18, *p* = 5.9 × 10^−5^; Fig. S6D). Moreover, these associations remained consistent when controlling for changes in protein abundance observed in the shotgun proteomics data using the partial Spearman correlation (*p* ≤ 2.6 × 10^−4^), when analyzing the data using linear regression (*p* ≤ 6.2 × 10^−3^; Fig. S6E), and when sampling with replacement from the underlying SEC-PCP-SILAC chromatograms (Fig. S6B-D). Collectively, these results indicate that rapidly evolving proteins are disproportionately rewired in the protein-protein interaction network by IFN signaling, to a degree that cannot be explained by changes in protein abundance alone, suggesting that species-specific differences in the innate immune response may be mediated in part through the effects of protein sequence divergence on protein-protein interactions.

### Functional landscape of IFN signaling

Despite the high quality of our IFN interactome, high-throughput maps of protein-protein interactions are unavoidably characterized by both false positives and false negatives [50]. We therefore sought to characterize the impact of IFN signaling on the human protein-protein interaction network more broadly, by developing a statistical framework for differential network analysis at the functional level (Additional file 1: Fig. S7A). Briefly, our approach first calculates the number, *n*_*PPI*_, of protein-protein interactions in both the stimulated and unstimulated networks involving proteins associated with a functional category of interest, and the difference between them, ∆*n*_*PPI*_. To assess statistical significance, the observed ∆*n*_*PPI*_ is compared to a randomized distribution obtained from the *n*_*PPI*_ values of 1000 randomly rewired networks. The network rewiring procedure controls for biases stemming from network topology that may be independent of functionally relevant patterns [23, 24, 51].

**Fig. 4:**
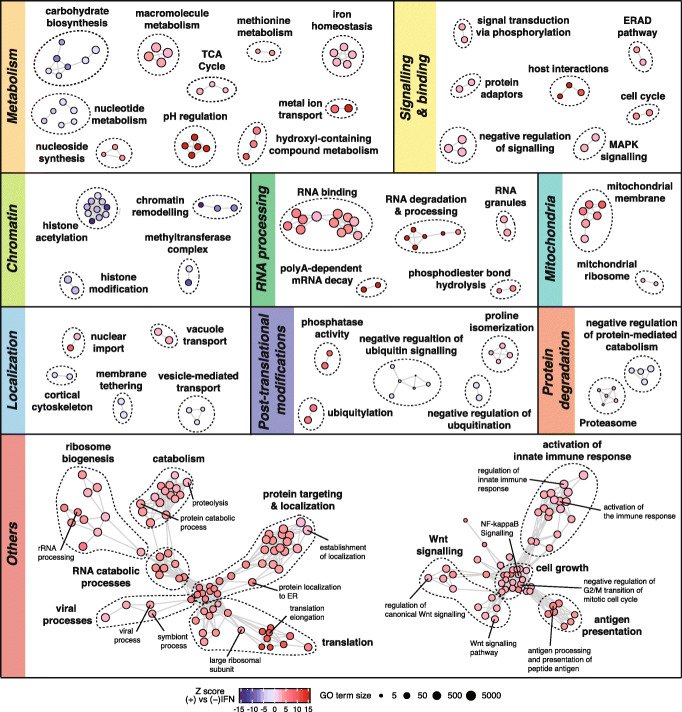
Differential network analysis of IFN-stimulated and unstimulated interactomes. Enrichment map of GO terms significantly enriched (red) or depleted (blue) in the IFN-stimulated interactome relative to the unstimulated interactome, at 20% FDR. Edges are defined between GO terms annotated to a common number of genes corresponding to a proteome-wide Jaccard index ≥ 0.33. Nodes clustered together in space therefore represent groups of functionally related GO terms and are grouped by major biological processes or pathways

A total of 341 GO terms were significantly enriched in either stimulated or unstimulated networks at 20% FDR and were visualized as an enrichment map [52] (Fig. 4b, Additional file 7). As expected, we observed a significant enrichment for interactions between proteins involved in the innate immune response in the IFN-stimulated network, including GO terms such as “antigen processing and presentation” (Fig. 4, Additional file 1: Fig. S7B). The Wnt signaling pathway was likewise enriched in the IFN-stimulated network, consistent with its link to type I IFN signaling [53, 54] (Additional file 1: Fig. S7B). Intriguingly, the stimulated network was also enriched for terms relating to RNA processing and splicing, suggesting a role for physical interactions in the regulation of alternative splicing during the host response to viral infection [1, 55]. Conversely, the stimulated network was depleted for interactions involving chromatin remodeling and histone-modifying machinery, as well as interactions involved in negative regulation of ubiquitination. Surprisingly, we observed a significant enrichment for interactions related to translation and ribosome biogenesis upon IFN stimulation (Fig. 4, Additional file 1: Fig. S7B), potentially accounting for the apparent lag observed in our shotgun proteomics experiment between ISG transcription and translation. To test whether these enrichments specifically reflected IFN-induced changes in interactome structure, as opposed to differences in the protein content of the stimulated and unstimulated networks, we performed a separate functional enrichment analysis of the proteins found in either network, but found little overlap between GO terms identified as enriched by interaction rewiring-based and protein content-based methods (Additional file 1: Fig. S7C). Collectively, these observations reflect wide-ranging functional changes within the human interactome induced by IFN signaling.

### Interactions between evolutionarily conserved ISGs modulate the type I IFN response

**Fig. 5:**
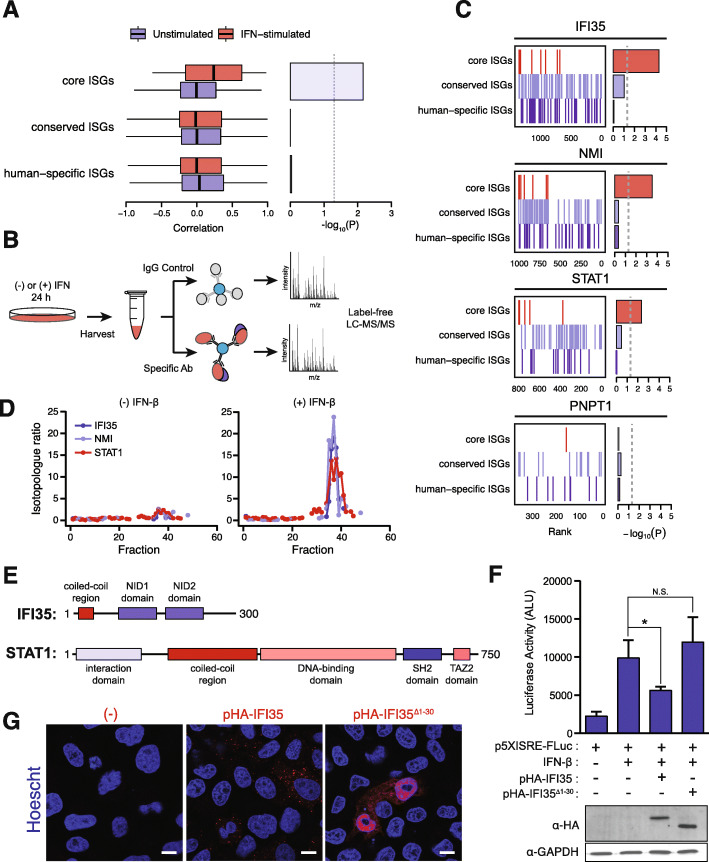
Interactions between evolutionarily conserved ISGs modulate the transcriptional response to IFN stimulation. **a** Left, distribution of correlations between pairs of proteins from core, conserved, and human-specific ISG sets in PCP chromatograms. Right, negative base-10 logarithm of *p* values from Brunner–Munzel tests of the difference in medians. **b** Schematic overview of the affinity purification–mass spectrometry experiments of specific core ISGs in IFN-stimulated or unstimulated cells. **c** Gene set enrichment analysis barcode plots [56], showing ranks of core, conserved, and human-specific ISG products in comparisons of immunoprecipitations of IFI35 (left), NMI (middle), and STAT1 (right) from IFN-stimulated or unstimulated cells, alongside negative base-10 logarithms of *p* values for each ISG set. **d** PCP chromatograms from a representative replicate of IFI35, NMI, and STAT1 in IFN-stimulated and unstimulated cells. **e** Protein domain content of IFI35 (top) and STAT1 (bottom). **f** Top, luciferase activities from cells transfected with a 5 × ISRE-Fluc reporter construct to monitor STAT1 transcriptional activity (± SD). Cells were transfected with equal amounts of DNA in each case. Bottom, western blots of HA-tagged IFI35 expression from lysates transfected with reporter constructs. *p < 0.05. **g** Immunofluorescence micrographs of cells mock transfected or transfected with HA-tagged IFI35 expression constructs. Shown is a representative image. Scale bar = 10 μm

Large-scale transcriptomic studies of the innate immune response across species and cell types have contrasted extensive heterogeneity in species- or cell type-specific transcriptional programs with a core module of universally upregulated genes, anchoring the transcriptional response across evolutionary and cellular contexts [7–9]. We sought to characterize the properties of evolutionarily conserved and species-specific ISGs at the interactome level. Using data from a comparative transcriptomic study of ten vertebrates [7], we defined sets of genes upregulated by IFN in all species (“core ISGs”), in human and at least one other species (“conserved ISGs”), or in humans only (“human-specific ISGs”). We then asked whether our SEC-PCP-SILAC data supported the hypothesis that ISGs in each category are induced to interact physically with one another in response to IFN stimulation. To address this hypothesis, we calculated the correlation between all pairs of ISGs in each condition and tested for a significant increase in the median correlation. Strikingly, only the core set of evolutionarily conserved ISGs displayed a significant shift in chromatogram correlation upon IFN stimulation (*p* = 6.9 × 10^−3^, Brunner–Munzel test; Fig. 5a), suggesting these ISGs enter physical interactions or protein complexes during the IFN response, at least within HeLa cells. Similar results were obtained when using the approach described in Additional file 1: Fig. S7A to test for network rewiring, with significantly more interactions between core ISGs in the IFN-stimulated network (*p* = 0.0072), but not conserved or human-specific ISGs (*p* ≥ 0.36). As expected, despite their evolutionarily conserved induction in response to IFN stimulation, these core ISGs are also rapidly evolving at the protein sequence level, consistent with the data presented in Additional file 1: Fig. S3 (*p* = 2.0 × 10^−8^, Brunner–Munzel test; Additional file 1: Fig. S8A).

To experimentally validate this observation, we performed co-immunoprecipitations of four core ISGs in unstimulated or IFN-stimulated cells (Fig. 5b, Additional file 8). In three of four co-immunoprecipitations, core ISGs were significantly and selectively enriched among interactors after IFN stimulation, relative to conserved or human-specific ISGs (gene set enrichment analysis, *p* ≤ 5.1 × 10^−3^; Fig. 5c). Moreover, this enrichment was robust to filtering potential non-specific interactors from the CRAPome database (Additional file 1: Fig. S8B) [57].

To shed light on the functional consequences of physical interactions between core ISGs, we focused on the interaction between IFI35 and STAT1, which was detected in IFN-stimulated cells by both SEC-PCP-SILAC (Fig. 5d, Additional file 4) and co-immunoprecipitation of STAT1 (Additional file 1: Fig. S8C). Notably, we could not recover STAT1 in immunoprecipitations of IFI35, although we could recover its known binding partner NMI [58, 59]. Immunoprecipitations of NMI, which has previously been shown to interact with STAT1 during IFNγ treatment [60], likewise recovered IFI35, but did not reproducibly recover STAT1 (Additional file 1: Fig. S8C). These observations suggest that the interaction between STAT1 and IFI35 may be substoichiometric, whereby not all cellular IFI35 is bound to STAT1, or may potentially occur in an IFNβ-specific manner. Furthermore, the interaction of IFI35 and STAT1 was also detected in a monocyte-derived cell line, THP-1 (Additional file 1: Fig. S8D), suggesting that this interaction is not limited to HeLa cells.

Given the role of IFI35 in repression of the IFN response through other mechanisms such as promoting degradation of RIG-I and decreasing IFNβ production [61, 62], we hypothesized that the interaction between IFI35 and STAT1 functioned to reduce STAT1 transcriptional activity as a potential mechanism to downregulate the IFN response. To investigate this hypothesis experimentally, we made use of a reporter construct containing 5× Interferon-Sensitive Response Elements (ISREs) fused to a firefly luciferase gene to monitor STAT1 transcriptional activity. Cells were transfected with the reporter construct, followed by transfection with a construct overexpressing hemagglutinin (HA)-tagged IFI35 and subsequent IFN stimulation. Control cells displayed low levels of luciferase activity that increased substantially upon IFN stimulation, as expected (Fig. 5f). Expression of an N-terminal HA-tagged IFI35 resulted in a significant decrease in luciferase activity, suggesting that STAT1 transcription is impaired (Fig. 5f). To further understand the mechanism underlying STAT1 transcriptional repression, we hypothesized that the IFI35–STAT1 interaction may be mediated by the N-terminal coiled-coil domain of IFI35, with the internal coiled-coil domain of STAT1 its potential interaction partner (Fig. 5e). Consistent with this hypothesis, truncation of the coiled-coil domain from IFI35 recovered luciferase activity in transfected cells, despite expression of the truncated protein at similar levels as the full length (Fig. 5f). Of note, unlike previous work on RIG-I, [61], we did not observe a decrease in STAT1 protein levels upon overexpression of IFI35, suggesting substantial degradation is likely not occurring (Additional file 1: Fig. S8E). To determine if truncating the coiled-coil region perturbs IFI35 function, we examined the localization of wild-type HA-IFI35 or its truncated form upon IFN stimulation. Immunofluorescent staining revealed that wild-type HA-IFI35 formed punctate cytoplasmic granules with IFN stimulation, as previously observed (Fig. 5g) [63, 64]. Strikingly, truncation of the first 30 N-terminal amino acid residues of IFI35 dramatically shifted the localization, to diffuse cytoplasmic and nuclear staining (Fig. 5g). This data suggests that disruption of the IFI35 N-terminal region may alleviate inhibition of STAT1 activity by redistributing IFI35 localization in the cytoplasm.

Taken together, these data support a model whereby IFI35 interacts with STAT1, sequestering it to cytoplasmic granules to fine-tune STAT-1-mediated transcription in response to IFN stimulation.

### Ribosomal incorporation of RPL28 buffers ISG protein synthesis

Motivated by the observation of significant enrichment for interactions involved in translation and ribosome biogenesis in the IFN-stimulated interactome (Fig. 4, Additional file 1: Fig. S7B), we investigated the relationship between the mRNA and protein levels of ISGs in greater detail. Examining the expression of ISGs at the mRNA level in a densely sampled time-course transcriptomic experiment [8], we found proteins that were differentially expressed after 4 h of IFN stimulation reached peak transcriptional levels between 0.5 and 3.5 h (Additional file 1: Fig. S8A). Surprisingly, however, a large proportion of proteins differentially expressed at 24 h had peak mRNA expression at similar timepoints (Additional file 1: Fig. S8B), suggesting a lag in their translation. Based on these observations, we hypothesized that a specialized translational program, potentially mediated by changes in ribosome composition [65], may be involved in establishing the IFN response.

**Fig. 6:**
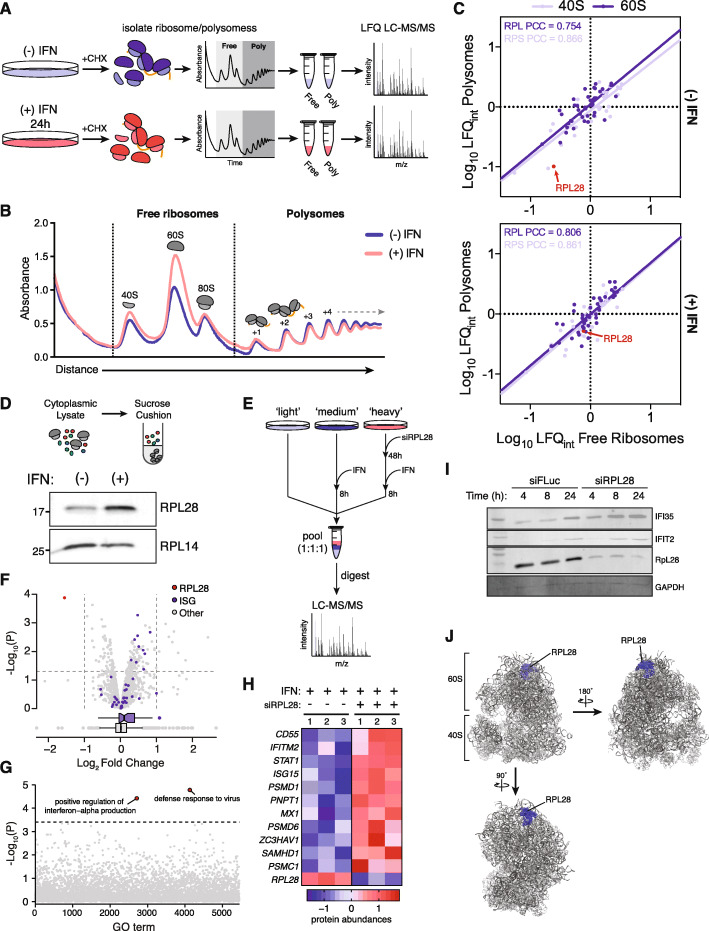
IFN stimulation induces changes in ribosome composition to regulate ISG synthesis. **a** Schematic overview of the sucrose gradient experiments to determine changes in composition of free (40S, 60S, and 80S) and actively translating ribosomes (polysomes). **b** Representative traces of sucrose density gradients from cells stimulated with IFN for 24 h or unstimulated cells. **c** Median protein abundance across three replicates from pooled free ribosome or polysome fractions by label-free quantification (LFQ) in IFN-stimulated or unstimulated cells. PCC, Pearson’s correlation coefficient. **d** Western blot of ribosomes isolated from IFN-stimulated or unstimulated cells by sucrose cushion. **e** Experimental workflow for shotgun proteomics analysis on RPL28-depleted cells after IFN stimulation. **f** Volcano plot showing differential protein abundance in cells stimulated with IFN for 8 h and treated with siRPL28, relative to controls. **g***p* values from gene set enrichment analysis (GSEA) of 5453 GO terms in a comparison of siRPL28-treated and IFN-stimulated cells compared to untreated controls. Dotted line shows the statistical significance of ISGs by GSEA (Fig. S9E). Points in red represent GO terms more significantly enriched than ISGs. **h** Heatmap showing abundance of select well-studied ISGs in siRPL28-treated and IFN-stimulated cells compared to untreated controls. **i** Western blots of select ISGs in cells treated with siRPL28 or control siRNA after 4 h, 8 h, and 24 h of IFN stimulation. **j** Crystal structure of the human 80S ribosome (PDB: 4UG0), with RPL28 highlighted in blue

To address this hypothesis, we performed sucrose density gradients to isolate free (40S, 60S, and 80S) and actively translating ribosomes (polysomes) in IFN-stimulated or unstimulated cells (Fig. 6a). Polysome traces revealed an increase in free ribosomes upon IFN stimulation, apparent from the heightened 40S and 60S peaks, but no detectable changes in polysome levels (Fig. 6b). To investigate changes in ribosome composition, fractions containing free ribosomes and polysomes, respectively, were pooled and subjected to quantitative mass spectrometry. Most ribosomal proteins were incorporated at similar levels in IFN-stimulated and unstimulated samples (Fig. 6c, Additional file 9). However, we observed a substantial and selective increase in RPL28 incorporation upon IFN stimulation (Fig. 6c). We confirmed this observation by western blots of isolated ribosomes from IFN-stimulated or unstimulated cells. A substantial increase in RPL28 was observed compared to a control ribosomal protein, RPL14, suggesting RPL28 is selectively incorporated into ribosomes during the IFN response (Fig. 6d).

We next sought to characterize the functional consequences of RPL28 incorporation during the IFN response. Over the past decade, it has become clear that ribosomes may not exist as a single homogenous population, but instead undergo dynamic changes in composition, with incorporation of individual ribosomal components forming “specialized ribosomes” that promote the translation of specific mRNA classes [66–69]. Based on this body of evidence, and the fact that RPL28 lies in a solvent-accessible portion of the ribosome (Fig. 6j), we hypothesized that IFN-dependent RPL28 incorporation facilitates translation of mRNAs related to the type I IFN response. In support of this hypothesis, examination of the Human Protein Atlas [70] and FANTOM5 database [71] indicated RPL28 is expressed at the highest levels in human immune system tissues, such as the thymus and lymph nodes (Additional file 1: Fig. S8C–D), consistent with a potential immunological role.

To determine the functional role of RPL28, we used siRNAs to knockdown RPL28 followed in the context of IFN stimulation to precisely quantify changes in protein synthesis. Cells labeled with heavy isotopes were treated with siRPL28 for 48 h, after which both medium- and heavy-labeled cells were exposed to IFN for 8 h, with unstimulated light-labeled cells serving as a baseline control (Fig. 6e). A total of 1940 proteins were identified, of which 1421 were quantified in at least two of three replicates (Additional file 10). To our surprise, comparison of siRPL28-treated cells to controls revealed an increase in ISG abundance upon RPL28 knockdown (Fig. 6f, h), an effect which was highly significant by gene set enrichment analysis (*p* = 2.9× 10^−4^; Fig. 6g). An unbiased enrichment analysis of the RPL28 knockdown data identified only two GO terms with a more statistically significant effect (Fig. 6g, Additional file 11), both of which (“positive regulation of interferon-alpha production” and “defense response to virus”) overlapped substantially with proteins having known roles in the IFN response. We further confirmed this finding by western blot, observing increases in ISG abundance over time in the RPL28 knockdown compared to an siRNA control (Fig. 6i). Importantly, to rule out the possibility that RPL28 leads to impaired ribosome biogenesis and global downregulation of translation rates [72], we monitored protein synthesis by metabolic labeling with [^35^S]-Met/Cys, finding that RPL28 depletion did not alter global protein levels in either our SILAC experiment (Fig. 6f) or when assessed with metabolic labeling (Additional file 1: Fig. S9E), suggesting that the ribosome remains functionally competent.

We next asked whether this phenomenon is specifically associated with knockdown of RPL28,or whether it occurs in response to perturbation of ribosomal proteins more generally. To this end, we compared the proteome of IFN-stimulated cells after treatment with siRNAs targeting RPL28, RPS26, or RPS28 using label-free quantitation (Additional file 12). Following RPL28 knockdown, we reproduced the results from our first experiment, observing a significant increase in ISG abundance upon RPL28 depletion (*p* = 7.1 × 10^−4^; Additional file 1: Fig. S10A). However, this effect was not observed after depletion of RPS26 or RPS28 (*p* = 0.69 and 0.18, respectively; Additional file 1: Fig. S10B-C), indicating that the increase in ISG abundance is specific to perturbation of RPL28.

Finally, it is possible that the changes observed in ISG abundance are merely due to an increase in mRNA levels. To further rule out the possibility that the changes in ISG protein abundance are mediated primarily by an increase in mRNA levels, we performed RT-qPCR, which did not reveal a significant difference between siRPL28- or control-treated cells (with the exception of NMI; Additional file 1: Fig. S9F). Taken together, these results suggest that RPL28 is specifically incorporated into ribosomes upon IFN stimulation, where it acts to selectively downregulate ISG protein synthesis.

## Discussion

The pleiotropic effects of type I IFN stimulation on transcriptional regulation have been appreciated for over two decades [2]. In turn, the maturation of increasingly sensitive technologies for transcriptome profiling, complemented by functional assays, has led to the identification of hundreds of ISGs [6]. Yet, with relatively few exceptions, the functional roles of these effectors in the antiviral response remain incompletely delineated. In particular, little is known about how existing cellular networks are influenced by IFN stimulation and how newly synthesized ISGs engage these complex networks.

Here, we have used protein correlation profiling to construct a differential network map of the human protein-protein interactome in response to type I IFN signaling. This map identifies specific interactions for known ISGs under homeostatic conditions and reveals patterns of interaction rewiring induced by IFN stimulation. The IFN interactome thus places ISGs into a functional context, providing a platform for further mechanistic dissection of their roles in the innate immune response. For example, we find that IFI35 binds to STAT1 to potentially fine-tune its transcriptional activity, further defining the role of IFI35 as a direct negative regulator of the IFN response. Given the dysregulation of IFN signaling in both monogenic diseases [5], as well as a broader spectrum of autoimmune and neuropsychiatric disorders [4], our work provides a framework to develop a deeper understanding of the mechanisms that protect against inappropriate immune activation.

Our functional analysis of the IFN-induced differential interactome implicated rewiring of interactions involved in protein translation and ribosome biogenesis in the IFN signaling cascade, leading us to uncover a novel regulatory mechanism involved in the innate immune response. We find that increased incorporation of RPL28 into the ribosome upon IFN stimulation represses synthesis of ISGs, whereas global translation remains unaffected. This effect was not observed when we perturbed other components of the ribosome, suggesting specificity to RPL28. Given the need for tight regulation of the IFN response to avoid aberrant overactivation, RPL28 may act as a buffer on excessive ISG translation, in effect imposing a secondary layer of regulation beyond mRNA transcription. Overall, this finding adds to a growing body of literature demonstrating how modulation of ribosome composition may facilitate translational control of specific mRNA classes [67–69].

After 24 h of IFN stimulation, we observed dynamic rearrangements of the cellular protein-protein interaction network, affecting a broad spectrum of cellular pathways. At this relatively late timepoint, the observed interactome rewiring likely reflects not only the direct effects of IFN signaling, but also the downstream effects of IFN stimulation on cellular processes such as chromatin remodeling and metabolism, as observed in our shotgun proteomics dataset (Fig. 1). These changes in protein abundance and network connectivity may reflect the establishment of an antiviral state in uninfected “bystander” cells exposed to circulating IFN, similar to that observed during viral infections [73–75].

Our differential network map utilized HeLa cells to delineate the impact of IFN stimulation on the human interactome. HeLa cells have been widely used as a model to understand viral infections and the immune response, and express core type I IFN pathway components common to most cell types, including the ubiquitously expressed type I IFN-α/β receptor (IFNAR) [76] and downstream signaling components, such as JAK1, STAT proteins, and IRF9. Our experimental validation of the IFN-dependent interaction between STAT1 and IFI35 in a second cell type, the monocyte-derived cell line THP-1, suggests that at least a subset of the interactions reported here may be conserved across cell types. However, in view of the variable strength and specificity of the IFN transcriptional response across different cell types [8, 9], it is likely that a subset of interactions are also cell type-specific, a possibility that warrants further investigation. Moreover, in this work, we directly stimulated cells with IFN, thus bypassing the need for cytoplasmic or endosomal sensors to trigger an IFN response. While this provides a direct basis to understand IFN-dependent interactome remodeling, future studies making use of different activating ligands, such dsRNA, lipopolysaccharide, other IFNs, or viral infection, will be required in order to define how the interactome of human cells is rewired to establish selective responses to distinct stimuli.

Charting macromolecular interaction networks in a physiologically relevant and differential context, particularly at the proteome scale, represents a long-standing challenge [16]. Our results highlight the unique power of protein correlation profiling, in combination with SILAC labeling and size exclusion chromatography, to systematically resolve interaction dynamics in the innate immune response, or in response to cellular perturbations more broadly [17, 77]. Widely used methods for interactome mapping, such as yeast two-hybrid or affinity purification-mass spectrometry, rely on heterologous expression of fusion proteins or the introduction of a protein tag; the former removes proteins completely from their endogenous cellular context, whereas the latter can disrupt the native interactions or subcellular localization of the tagged protein [78]. Thermal proximity co-aggregation (TPCA) has demonstrated promise for interrogating protein interaction networks in vivo, or across distinct cellular states [79–81], but to date has been limited to monitoring the dynamics of known interactions or protein complexes, rather than enabling de novo network inference. In contrast, the primary disadvantage associated with SEC-PCP-SILAC is its moderate bias towards proteins of greater cellular abundance [82]. In combination with the computational tools for differential network analysis described here, SEC-PCP-SILAC represents a powerful, untargeted approach to define rearrangements in the human interactome in response to cellular stimuli.

On the other hand, several limitations of SEC-PCP-SILAC, and co-fractionation approaches more generally, should be noted. Although SEC-PCP-SILAC provides a basis for inference of co-complex membership based on correlated protein abundance across conditions designed to separate protein complexes based on their size, it does not provide direct evidence of physical protein-protein interactions as such. Moreover, some authors have drawn a distinction between methods that detect “binary” interactions, which ostensibly represent direct biophysical contacts between protein pairs, and those that detect “co-complex” interactions, which represent all pairs of proteins in the same protein complex as physically interacting [83, 84]. Whether one type of interaction is more biologically meaningful than another has been a matter of some debate, and it is noteworthy that many literature-curated interaction databases and standardized formats for molecular interaction data do not draw such a distinction [85–87]. Nonetheless, the fact that not all of the interacting protein pairs reported by PrInCE will be in direct biophysical contact can also be viewed as a limitation of the resource presented here, although some evidence exists that direct contacts can also be recovered from the underlying protein correlation profiles using more bespoke methods [88]. A more general limitation is that our study employed a relatively lenient precision threshold of 70%. While the results presented here indicate the IFN interactome has value as a resource for discovery, and for systems-level interrogation of the innate immune response, any of the individual putatively novel interactions detected here requires independent confirmation by an orthogonal experimental method to be considered reliable, as is indeed the case for any high-throughput interactome mapping technique.

To facilitate exploration of the complete dataset, we have developed an interactive web application, available at https://ifn-interactome.msl.ubc.ca, that allows users to visualize both the IFN interactome network as well as the underlying PCP chromatograms. The network view further allows users to optionally restrict the network either to specific genes that are of interest, or to entire sets of genes called as differentially expressed in transcriptomic meta-analyses of 103 different diseases, including the IFN-related diseases depicted in Fig. 3i [42, 43].

## Conclusions

In sum, the map of the IFN-induced interactome presented here systematically expands our understanding on the organization of the innate immune response while complementing previous functional and systems-level studies, providing a rich resource to inform hypothesis-driven experiments. Our data reveals a surprisingly broad spectrum of rewiring in cellular pathways induced by IFN and uncovers novel regulatory mechanisms at the levels of transcription and translation that modulate the IFN response. Intersecting this network map with data from genome-wide association or exome sequencing studies could prove an effective strategy to further understand how these pathways are perturbed by common or rare variants in the context of autoimmune or neuropsychiatric diseases [89, 90]. More broadly, our work establishes a proteomic and bioinformatic platform to delineate the complex networks of regulatory pathways activated in response to physiological or pathophysiological stimuli.

## Methods

### Cell culture and IFNβ stimulation

HeLa cells were cultured in Dulbecco’s modified Eagle’s medium (DMEM) supplemented with 10% fetal bovine serum (FBS), 1× penicillin-streptomycin (Pen-Strep), and 2 mM l-glutamine at 37 °C. THP-1 cells were cultured in RPMI-1640 medium supplemented with 10% FBS, 0.05 mM 2-mercaptoethanol, and 1× Pen-Strep at 37 °C. For IFNβ stimulation, cells were seeded and incubated overnight at 37 °C. The following day, cells were washed with 1× phosphate-buffered saline (PBS) and stimulated with 1000 U/mL of human recombinant IFNβ (R&D Systems) in DMEM for the designated length of time. For SILAC experiments, cells were stimulated in the appropriate SILAC-formulated media.

### Plasmids and transfections

HA-tagged IFI35 was generated as follows. First, total RNA was isolated from IFNβ-stimulated (24 h) HeLa cells via TRIzol extraction (Thermo Fisher) followed by RT-PCR using an oligo dT primer. Desired sequences were amplified from cDNA using primers IFI35-F (5′–TAGGGTACCATGTCAGCCCCACTGGATGCCG–3′) and IFI35-R (5′–TAGCTCGAGCTAGCCTGACTCAGAGGTGAAGACTGC–3′). Amplicons were digested, followed by ligation into pcDNA3.1. Subsequently, a 3× HA tag was N-terminally fused onto IFI35, respectively, by amplifying the cloned sequences with primers that incorporated the tags and subcloning into the respective constructs. Constructs were confirmed through sequencing. The ISRE-Luc reporter construct was a gracious gift from Dr. Curt Horvath (Northwestern University) and contains 5× ISG54 ISRE elements upstream of a TATA box and a firefly luciferase open reading frame.

Transfections were done as follows: briefly, 3.0 × 10^5^ HeLa cells were seeded into 6-well plate and incubated for 24 h at 37 °C. Plasmids (2 μg) and transfection reagent (5 μL of Lipofectamine 2000; Invitrogen) was added to 125 μL of OptiMEM serum-free media (Thermo Fisher) in separate tubes and incubated for 5 min. Tubes were combined and incubated for 15 min. Media were aspirated from cells, and the 250-μL transfection mix was added to cells dropwise. Complete media were added to each well, and cells were incubated at 37 °C. For RPL28 and control siRNA experiments, cells were transfected as per manufacturer’s protocol at a final concentration of 25 nM using Dharmafect I transfection reagents (Dharmacon).

### Luciferase assays

Luciferase assays were carried out using a Luciferase Assay System Kit (Promega). Briefly, cells transfected in a 6-well plate as described above were harvested and washed with 1× PBS. Cells were then lysed using 1× Passive Lysis Buffer as per manufacturer’s protocol (Promega). Protein concentration was then determined via Bradford assay. Equal amounts of protein (30 μg) were added to a Costar Flat White 96-well plate for each condition, and samples were brought to equal volume with 1× Passive Lysis Buffer. Following this, 50 μL of luciferase reagent was added to each well and luminescence was recorded on an Infinite M200 microplate reader (Tecan).

### Western blots

Equal amounts of protein were resolved on a 12% SDS-PAGE gel and then transferred to a polyvinylidene difluoride Immobilon-FL membrane (PVDF; Millipore). Membranes were blocked for 30 min at room temperature with 5% skim milk in TBST (50 mM Tris, 150 mM NaCl, 1% Tween-20, pH 7.4). Blots were incubated for 24 h at 4 °C with the following antibodies: mouse anti-GAPDH (1:1000; AbLab), rabbit anti-HA (1:1000; Cell Signalling—C29F4), rabbit anti-RPL14 (1:1000; Bethyl Laboratories—A305-052A), rabbit anti-RPL28 (1:1000; AbCam—ab138125), mouse anti-IFIT2 (1:1000; Santa Cruz—sc-390,724), mouse anti-IFI35 (1:1000; Santa Cruz—sc-100,769), or rabbit anti-NMI (1:1000; AbCam—ab183724). Membranes were washed 3 times with TBST and incubated with either IRDye 800CW goat anti-mouse (1:5000; Li-Cor Biosciences) or IRDye 800CW goat anti-rabbit (1:5000; Li-Cor Biosciences) for 1 h at room temperature. Membranes were then washed 3 more times with TBST before imaging on an Odyssey imager (Li-Cor Biosciences).

### Metabolic labeling

HeLa cells were transfected with siFluc or siRPL28 for 48 h before being stimulated with IFNβ for 4 or 8 h and labeled with 250 μCi [^35^S]-Met/Cys for 30 min. Cells were washed twice with 1 mL PBS and harvested with 100 μL RIPA buffer. Equal amounts of lysates were loaded on 12% SDS-PAGE gels. Gels were dried and radioactive bands were analyzed using a phosphorimager (GE Amersham Typhoon). To quantify incorporated [^35^S]-Met/Cys, 20 μg of protein was precipitated with 25% trichloroacetic acid (TCA) before being filtered through a glass fiber filter. Subsequently, the filter was washed three times with 5% TCA followed by 100% acetone. The filter was suspended in scintillation fluid and analyzed on a liquid scintillation counter (Perkin Elmer).

### Immunofluorescence

Cells were seeded onto coverslips in 6-well plates and allowed to adhere overnight at 37 °C. The following day, cells were transfected with the respective constructs and incubated for 24 h at 37 °C. Subsequently, cells were washed twice with 1× PBS and fixed with 3% paraformaldehyde for 15 min. Cells were washed with PBS and cells were permeabilized with 0.2% Triton X-100 in PBS for 30 min. Next, cells were blocked for 30 min with Blocking Solution (3% BSA and 0.2% Triton X-100 in PBS), followed by incubation with primary antibody in Blocking Solution for 1 h at room temperature. Cells were washed three times with 1× PBS and incubated with secondary antibodies in 2% BSA plus PBS. Following this, cells were washed three times with 1× PBS and incubated with Hoechst dye (1:20,000 in PBS) for 15 min. Finally, coverslips were washed with 1× PBS and mounted onto a slide for imaging. Slides were imaged on a Leica SP5 confocal microscope with a × 63 oil objective lens and a × 2 digital zoom. Primary antibodies used are rabbit anti-HA (1:1000, Cell Signalling—C29F4). Secondary antibodies used are anti-rabbit Texas Red (1:20,000).

### Polysome and ribosome isolation

HeLa cells (1.0 × 10^7^) were seeded in 150 mM tissue culture plates for each condition and incubated for 24 h at 37 °C. Media were aspirated and replaced with either control media or media containing 1000 U/mL IFNβ, and cells were incubated for 24 h at 37 °C before being subjected to polysome or ribosome isolation. For whole ribosome isolation, cells were lysed with Ribosome Lysis Buffer (300 mM NaCl, 15 mM Tris-HCl, 6 mM MgCl_2_, 1% Triton X-100, 1 mg/mL heparin, pH 7.5). Lysates were clarified by centrifugation at 20,000 r.c.f. for 10 min at 4 °C. Clarified lysates were layered over a sucrose cushion at a 1:1 (v/v) ratio (2 M sucrose in Ribosome Lysis Buffer) followed by centrifugation at 100,000 r.c.f. for 24 h at 4 °C. Pelleted ribosomes were resuspended in RIPA buffer (50 mM Tris, 150 mM NaCl, 0.1% SDS, 0.5% sodium deoxycholate, 1% Triton X-100, 0.5 mM EDTA), and protein concentration was quantified by Bradford assay (BioRad). Equal amounts of protein were used for western blot analysis.

For polysome analysis, after treatment with IFNβ, cycloheximide (100 μg/mL) was added to the media and cells were incubated for 5 min. Cells were washed 3 times with 1× PBS plus cycloheximide (100 μg/mL) before being lysed in 400 μL of Polysome Lysis Buffer (300 mM NaCl, 15 mM Tris-HCl, 15 mM MgCl_2_, 100 μg/mL cycloheximide, 1 mg/mL heparin). Lysates were clarified by serial centrifugation at 800 r.c.f. for 5 min at 4 °C, then 13,000 r.c.f. for 10 min at 4 °C. RNA was quantified via NanoDrop, and equal amounts of RNA (500 μg) were loaded onto a linear 10–50% sucrose gradient made in Polysome Lysis Buffer. Gradients were centrifuged in a SW41 Ti Rotor (Beckman) for 2.5 h at 40,000 RPM at 4 °C. Fractions were collected on a Gradient Station IP Fractionator (BioComp). Fractions (750 μL) corresponding to free ribosomes (40S, 60S, and 80S monosomes) and polysomes were pooled, and proteins were precipitated using trichloroacetic acid. Resulting protein pellets were reduced, alkylated, and subjected to trypsin digestion before being analyzed by LC-MS/MS.

### SILAC labeling and shotgun mass spectrometry

HeLa cells were cultured in ­DMEM (Lys/Arg^−/−^) supplemented with 10% dialyzed FBS (Invitrogen), 1× Pen-Strep, and combinations of the following lysine and arginine isotopologues: for “light” (“L”)-labeled cells, l-arginine (84 mg/L) and l-lysine (146 mg/L) (Sigma-Aldrich); for “medium” (“M”)-labeled cells, ^13^C_6_-l-arginine (87 mg/L) and D_4_-l-lysine (150 mg/L); and for “heavy” (“H”)-labeled cells, ^13^C_6_^15^N_4_-l-arginine (89 mg/L) and ^13^C_6_^15^N_2_-l-lysine (154 mg/L) (Cambridge Isotope Laboratories). Cells were split into each SILAC formulation and passaged six times to allow for complete incorporation of amino acid isotopologues.

For shotgun proteomic analysis, ~ 1.0 × 10^7^ cells were harvested from control (light), 4 h IFNβ stimulation (medium), and 24 h IFNβ stimulation (heavy). Cells were lysed in Lysis Buffer (4% SDS, 10 mM DTT, 100 mM Tris-HCl, pH 8.8) and heated at 95 °C for 5 min. Samples were then centrifuged for 10 min at 16,000 r.c.f. at 4 °C, and the supernatant was collected. Protein concentrations were then measured via BCA assay (Thermo Fisher). One hundred micrograms of protein from each sample (light, medium, and heavy) was combined and subjected to acetone precipitation. Protein pellets were resuspended in a 6 M/2 M urea/thiourea mixture. Samples were reduced and alkylated by adding 6 μg of DTT and 15 μg of iodoacetamide and incubating at room temperature in the dark for 30 min and 20 min, respectively. Three micrograms of LysC was added to each sample and incubated for 3 h at room temperature. Subsequently, samples were diluted with 4 volumes of Digestion Buffer (50 mM NH_4_HCO_3_) and trypsin (Promega) was added at a ratio of 1:50. Samples were incubated shaking overnight at room temperature. The resulting peptide supernatant was acidified to pH < 2.5 and purified using homemade Stop-and-go-extraction tips (StageTips) composed of C18 Empore material (3 M) packed in to 200 μL pipette tips [91]. StageTips were conditioned with methanol and equilibrated with 1% trifluoroacetic acid (TFA; loading buffer). Peptide supernatants were loaded onto the columns and washed with two bed volumes of buffer A (0.5% formic acid). Peptides were eluted with buffer B (80% MeCN, 0.5% formic acid), dried down. Peptides from each biological replicate were then subjected to high pH reverse-phase (RP) fractionation on an Agilent 1100 HPLC system with an Agilent Zorbax Extend column (1.0 × 50 mm, 3.5 μm particles, flow rate of 50 μL/min). Dried peptides were resuspended in RP buffer A (5 mM NH_4_HCO_2_, 2% MeCN, pH 10), injected, and eluted from the column over a 60-min gradient: 0 to 5 min 6% RP buffer B (5 mM NH_4_HCO_2_, 90% MeCN), 5–7 min 8% RP buffer B, 7–45 min 27% RP buffer B, 45–49 min 31% RP buffer B, 49–53 min 39% RP buffer B, and 53–60 min 60% RP buffer B. The column was washed by running 100% RP buffer B for 5 min. Fractions were collected every 40 s for 60 min. Every eighth fraction was then concatenated, dried, and resuspended in buffer A for mass spectrometry analysis.

Purified peptides were analyzed using an Easy nano LC 1000 nanoflow HPLC (Thermo Fisher) on-line coupled to a Q-Exactive mass spectrometer (Thermo Fisher). The LC was operated in a trapping mode (two column system) using a 4-cm-long, 100-μm-inner-diameter fused silica trap column. The analytical column was from 75-μm-inner-diameter fused silica capillary, and it was either with an integrated spray tip, or it was fritted and attached to a 20-μm-inner-diameter fused silica gold coated spray tip. Columns with spray tip and spray tips for fritted columns were pulled on a P-2000 laser puller from Sutter Instruments to 6-μm-diameter opening. Added spray tips were coated on EM SCD005 Super Cool Sputtering Device (Leica). The trap column was packed with 5-μm-diameter Aqua C-18 beads (Phenomenex) to 2 cm, while the analytical column was packed with 3.0-μm-diameter Reprosil-Pur C-18-AQ beads (Dr. Maisch). The trap column was conditioned with 20 μL buffer A, and the analytical column was conditioned with 4 μL of the same buffer. Samples were loaded with 20 μL of buffer A. The analysis was performed at 250 nL/min over 180 min with a gradient from 0 to 40% buffer B over 180 min, then from 40 to 100% over 2 min and held at 100% B over 10 min. The LC autosampler thermostat was set at 7 °C. The Q-Exactive was operated in a data-dependent mode using Xcalibur v.2.2 (Thermo Fisher) and set to acquire a full-range scan at 70,000 resolution from 350 to 2000 Th (AGC target 3E6) and to fragment the top ten multiply charged ions above 5% underfill ratio by HCD (resolution 17,500, AGC target 1E5, maximum injection time 60 ms, NCE 28) in each cycle. Parent ions were then excluded from MS/MS for the next 25 s. Error of mass measurement is typically within 5 ppm and was not allowed to exceed 10 ppm.

### SEC-PCP-SILAC sample preparation

Cell lysis and size exclusion chromatography were performed as previously described [17, 77], with minor modifications. Briefly, after 24 h treatment with IFNβ, cells were immediately harvested by centrifugation at 200 r.c.f. for 5 min at 4 °C and washed three times with ice-cold 1× PBS. Cells of the same SILAC label were pooled and resuspended in 3 mL of ice-cold size-exclusion chromatography (SEC) buffer [50 mM KCl, 50 mM NaCH_3_COO, 50 mM Tris, pH 7.2, containing 1× EDTA-free HALT protease & phosphatase inhibitor cocktail (Thermo Fisher)]. Cells were lysed via Dounce homogenization for 2.5 min, and insoluble material was removed by ultracentrifugation at 100,000 r.c.f. for 15 min at 4 °C. Subsequently, the supernatants were concentrated over a 100-kDa molecular weight cutoff spin column (Sartoris Stedim, Goettingen, Germany). Equal amounts of protein from heavy-labeled and medium-labeled lysates were combined and immediately injected into a chromatography systems with two 300 × 7.8 mm BioSep4000 Columns (Phenomenex) equilibrated with SEC buffer. Samples were collected into 80 fractions by a 1200 Series analytical HPLC (Agilent Technologies) at a flow rate of 0.5 mL/min at 8 °C. To avoid aggregated proteins and small monomers, only fractions 6–65, corresponding to molecular weights ~ 2 mDa–65 kDa as approximated by the use of common standards thyroglobulin, apoferritin, and bovine serum albumin (Sigma-Aldrich), were submitted for LC-MS/MS analysis and utilized for PCP. The light-labeled SILAC lysates consisted of unstimulated and IFN-stimulated cells to serve as an internal standard. These samples were independently separated by SEC from the medium/heavy samples. To generate the light reference mixture, fractions 6–65 were pooled and spiked equally into each of the corresponding medium/heavy fractions at a volume of 1:0.75 (medium/heavy to light). A urea/thiourea mix was added to protein fractions to create a final concentration of 6 M/2 M urea/thiourea. Samples were reduced and alkylated by adding 6 μg of DTT and 15 μg of iodoacetamide and incubating at room temperature in the dark for 30 min and 20 min, respectively. Three micrograms of LysC was added to each sample and incubated for 3 h at room temperature. Subsequently, samples were diluted with 4 volumes of Digestion Buffer (50 mM NH_4_HCO_3_) and trypsin (Promega) was added at a ratio of 1:50. Samples were incubated shaking overnight at room temperature. The resulting peptides were purified via STAGE tips.

### Immunoprecipitation mass spectrometry sample preparation

HeLa cell lysates subjected to IP-MS analysis were prepared similarly as described for SEC-PCP-SILAC, apart from isotopologue labeling. In short, control and IFN-stimulated HeLa were harvested via centrifugation at 200 r.c.f. for 5 min at 4 °C, then washed three times with ice-cold PBS. Cells were then lysed via Dounce homogenization in SEC buffer followed by centrifugation at 100,000 r.c.f. Supernatants were concentrated over a 100-kDa molecular weight cutoff spin column (Sartoris Stedim, Goettingen, Germany). Protein concentration was determined by NanoDrop (Thermo Fisher). Two hundred fifty micrograms of protein from control or IFN-stimulated samples was diluted to 500 μL in SEC buffer and incubated with the desired antibody or IgG as a control overnight at 4 °C. Antibody concentrations were used as follows: mouse anti-IFI35 (1:50; Santa Cruz—sc-100769), rabbit anti-NMI (1:50; AbCam—ab183724), mouse anti-STAT1 (1:50; AbCam—ab3987), and mouse anti-PNPT1 (2 μg; Santa Cruz—ab-271,479). Protein A/G Magnetic beads (25 μL; Thermo Fisher) pre-washed with SEC buffer were added to the samples and incubated 1 h at room temperature. Beads were washed three times with 20× bed volume of SEC buffer. Samples were then subjected to an on-bead in-solution trypsin digestion. Peptides were purified by STAGE tips.

### Tandem liquid chromatography mass spectrometry of SEC-PCP-SILAC and LFQ samples

Purified peptides were analyzed using a quadrupole time of flight mass spectrometer (Impact II; Bruker Daltonics) on-line coupled to an Easy nano LC 1000 HPLC (Thermo Fisher) using nanoBooster with methanol and a Captive spray nanospray ionization source (Bruker Daltonics) including a 2-cm-long, 100-μm-inner-diameter fused silica fritted trap column, and 40-cm-long, 75-μm-inner-diameter fused silica analytical column with an integrated spray tip (6–8-μm-diameter opening, pulled on a P-2000 laser puller from Sutter Instruments). The trap column was packed with 5 μm Aqua C-18 beads (Phenomenex) while the analytical column was packed with 1.9-μm-diameter Reprosil-Pur C-18-AQ beads (Dr. Maisch). Buffer A consisted of 0.1% aqueous formic acid, and buffer B consisted of 0.1% formic acid in acetonitrile. Samples were resuspended in buffer A and loaded with the same buffer. Standard 90-min gradients were from 0% B to 35% B over 90 min, then to 100% B over 2 min, held at 100% B for 15 min. Before each run, the trap column was conditioned with 20 μL buffer A, the analytical with 4 μL of the same buffer, and the sample loading was set at 20 μL (for samples up to 13 μL volume). The LC thermostat temperature was set at 7 °C. The Captive Spray Tip holder was modified similarly to an already described procedure [92]. The fused silica spray capillary was removed (together with the tubing which holds it) to reduce the dead volume, and the analytical column tip was fitted in the Bruker spray tip holder using a piece of 1/16 in × 0.015 PEEK tubing (IDEX), an 1/16 in metal two-way connector, and a 16-004 Vespel ferrule. The sample was loaded on the trap column at 850 Bar, and the analysis was performed at 0.25 μL/min flow rate. The Impact II was set to acquire in a data-dependent auto-MS/MS mode with inactive focus fragmenting the 20 most abundant ions (one at a time at 18 Hz rate) after each full-range scan from m/z 200 Th to m/z 2000 Th (at 5 Hz rate). The isolation window for MS/MS was 2 to 3 Th depending on parent ion mass to charge ratio, and the collision energy ranged from 23 to 65 eV depending on ion mass and charge [92]. Parent ions were then excluded from MS/MS for the next 0.4 min and reconsidered if their intensity increased more than 5 times. Singly charged ions were excluded since in ESI mode peptides usually carry multiple charges. Strict active exclusion was applied. Error of mass measurement is typically within 5 ppm and was not allowed to exceed 10 ppm. The nano ESI source was operated at 1900 V capillary voltage, 0.20 Bar nanoBooster pressure, 3 L/min drying gas, and 150 °C drying temperature. The cross connector between the trap column, waste out capillary, and analytical column was grounded via a 0.4-mm platinum wire to prevent electrical corrosion of the LC S valve.

Mass spectrometry of HeLa cells after siRNA knockdown of RPL28, RPS26, or RPS28 was conducted as follows: peptide samples were purified by solid phase extraction on C-18 stage tips.

Purified peptides were analyzed using a timsTOF trapped ion mobility quadrupole time of flight mass spectrometer (timsTOF Pro; Bruker Daltonics) on-line coupled to an Easy nano LC 1000 HPLC (Thermo Fisher Scientific) using a Captive spray nanospray ionization source (Bruker Daltonics) including a 75-μm-inner-diameter, 40-cm-long fused silica analytical column with an integrated spray tip (6–8-μm-diameter opening, pulled on a P-2000 laser puller from Sutter Instruments). The analytical column was packed with 1.9-μm-diameter Reprosil-Pur C-18-AQ beads (Dr. Maisch), and it was heated to 50 °C using tape heater (SRMU020124, Omega) and in house built temperature controller with a temperature sensor (SA1-RTD-80, Omega) and a microprocessor controller (CN7500, Omega). Buffer A consisted of 0.1% aqueous formic acid and 2% acetonitrile in water, and buffer B consisted of 0.1% formic acid in 90% acetonitrile in water. Samples were resuspended in buffer A and loaded with the same buffer. The gradient was from 5% B to 15% B over 60 min, then to 37% B from 60 to 120 min, then to 90% B over 2 min, held at 90% B for 13 min. Before each run, the analytical column was conditioned with 4 μL of buffer A and the sample loading was set at 10 μL (for samples up to 3 μL volume). The LC thermostat temperature was set at 7 °C. The Captive Spray Tip holder was modified similarly to a previously described procedure [93]: the fused silica spray capillary was removed, together with the tubing which holds it, to reduce the dead volume, and the analytical column tip was fitted in the Bruker spray tip holder using a piece of 1/16 in × 0.015 PEEK tubing (gray PEEK, IDEX), an 1/16 in metal two way connector, and a 16-004 Vespel ferrule (Trajan Scientific). The sample was loaded on the trap column at 950 Bar, and the analysis was performed at 0.35 μL/min flow rate. timsTOF was run with OTOF Control v. 5.1 (Bruker). LC and MS were controlled with HyStar 4.1 (4.1.21.1, Bruker). The timsTOF was set to acquire in a data-dependent PASEF mode with fragmenting the 10 most abundant ions (one at the time at 18 Hz rate) after each full-range scan from m/z 100 Th to m/z 1700 Th. The collision energy was 42 eV. Parent ions were then excluded from MS/MS for the next 0.4 min and reconsidered if their intensity increased more than 5 times. Error of mass measurement was not allowed to exceed 10 ppm. The nano ESI source was operated at 1900 V capillary voltage, 3 L/min drying gas, and 180 °C drying temperature. Funnel 1 was set at 300 V, funnel 2 at 200 V, multipole RF at 200 V, deflection delta at 70 V, quadrupole ion energy at 5 eV, low mass at 200 Th, collision cell energy at 10 eV, collision RF at 1500 V, transfer time at 60 μs, and pre-pulse storage at 12 μs. PASEF was on with 10 PASEF scans for charges 0 to 4, target intensity 20,000 and intensity threshold 2500. Singly charged ions were filtered off using tims filtering polygon set on the timsTOF heatmap. Isolation width was 2 Th for m/z ≤ 700 Th and 3 Th for m/z ≥ 800 Th; collision energy was 42.0 eV. MS/MS repetition was 1 for intensity > 20,000, 2 for intensity 14,142 to 20,000, 3 for intensity 11,547 to 14,142, 4 for intensity 10,000 to 11,547, 5 for intensity 8944 to 10,000, 6 for intensity 8164 to 8944, 7 for intensity 7559 to 8164, 8 for intensity 7071 to 7559, 9 for intensity 6666 to 7071, and 10 for lower intensity. Exclusion window had mass width 0.015 Th and 1/K0 width 0.015 V.s/cm^2^.

### Protein identification and quantification

Protein identification and quantification was performed using MaxQuant version 1.5.3.30 [94, 95]. The data were searched against the *Homo sapiens* UniProt database [96]. For shotgun SILAC experiments, the following parameters were used: peptide mass accuracy, 20 ppm (ppm) for first search, 10 ppm for second search; trypsin enzyme specificity, fixed modifications, carbamidomethyl; variable modifications, methionine oxidation, deamidation (NQ), and N-acetylation (protein N-terminus); and all other parameters as preset. For SEC-PCP-SILAC experiments, the following parameters were used: peptide mass accuracy, 10 ppm; fragment mass accuracy, 0.05 Da; trypsin enzyme specificity; fixed modifications, carbamidomethyl; and variable modifications, methionine oxidation, deamidation (NQ), and N-acetylation (protein N-terminus). For shotgun SILAC and SEC-PCP-SILAC experiments, both the requantify and match between run options were enabled. For SEC-PCP-SILAC experiments, SILAC labels with a minimum ratio count of one were included. For immunoprecipitation experiments, label-free quantitation (LFQ) was enabled, with a minimum ratio count of two. For analysis of purified ribosome composition, LFQ was enabled with a minimum ratio count of one, due to the small size of ribosomal proteins. Only those peptides exceeding the individually calculated 99% confidence limit (as opposed to the average limit for the whole experiment) were considered as accurately identified. In all analyses, contaminants and reverse hits were removed using Perseus version 1.6.1.1 [97].

For timsTOF data, analysis was performed using MaxQuant 1.6.10.43. The search was performed against a database comprised of the protein sequences from the source organism plus common contaminants using the following parameters: peptide mass accuracy 10 ppm, fragment mass accuracy 0.05 Da, trypsin enzyme specificity, fixed modifications—carbamidomethyl, and variable modifications—methionine oxidation and N-acetyl peptides. Only those peptides exceeding the individually calculated 99% confidence limit (as opposed to the average limit for the whole experiment) were considered as accurately identified.

### Data analysis for shotgun proteomics of the IFN response

Differentially expressed proteins were identified using the one-sample moderated *t* tests implemented in limma [98], followed by Benjamini–Hochberg correction. Enriched Gene Ontology terms were identified for proteins with significant differential expression between conditions at 5% FDR using the conditional hypergeometric test [99] implemented in the GOstats R package [100], with terms from each branch of the ontology analyzed separately. Enrichment for protein-protein interactions between differentially expressed proteins was assessed using the InBioMap database [19]. The likelihood of the observed number of interactions between differentially expressed proteins was evaluated by randomly rewiring the InBioMap interactome 100 times using a degree-preserving algorithm [23] in order to maintain network topology, with the number of iterations for the edge rewiring algorithm set to 6.9 × the number of edges in each network [101]. Network analyses were implemented in the R package “igraph.”

### Removal of high-magnitude errors in protein quantitation

Errors in quantitation of either the heavy or light channel can lead to the introduction of spurious outliers into PCP-SILAC chromatograms. To minimize the impact of these outliers on downstream analysis of the PCP chromatogram matrices, we applied a recently developed algorithm, “MODERN” (Model-free Outlier detection for Robust Networks), to remove outliers prior to network reconstruction (Skinnider et al., unpublished data). The motivating assumption that underlies MODERN is that a single observation should not globally rewire the interaction profile of a protein across the entire network. The interaction profile of each protein is quantified in MODERN as the vector of Pearson’s correlation coefficients between that protein and all other proteins in the network. Each point in the chromatogram is removed in turn, and the interaction profile is recalculated upon removal of each point. The correlation between the original interaction profile and the interaction profile with a single point removed is defined as the autocorrelation. Autocorrelation statistics are converted to *z* scores, and outliers are defined as observations associated with an autocorrelation *z* score less than the normal distribution *z* score corresponding to a two-tailed family-wise error rate of 0.05, given the total number of points observed. Application of MODERN to the six PCP chromatogram matrices led to the removal of 1308 of 245,841 protein quantifications (0.53%). MODERN is available as an R package from https://github.com/fosterlab/modern.

### Protein-protein interaction network reconstruction

High-confidence protein-protein interaction networks were reconstructed from raw SEC-PCP-SILAC profiles using PrInCE [32], our open-source pipeline for co-fractionation data analysis, available at https://github.com/FosterLab/PrInCE. PrInCE first implements basic data cleaning and filtering functionality to restrict analysis to high-quality chromatograms. Briefly, single missing values are imputed as the mean of the two neighboring points, proteins with fewer than five observations are removed, and a sliding average with a width of five fractions is used to smooth the chromatogram. PrInCE then fits a mixture of one to five Gaussians to the smoothed chromatograms, performs model selection using the bias-corrected Akaike information criterion [102], and discards chromatograms that could not be fit by a mixture of Gaussians. A machine-learning procedure is then used to assign an interaction score to each pair of co-fractionation profiles using a naive Bayes classifier. Features used by the classifier include the Pearson correlation of the raw chromatograms and its corresponding *p* value, the Euclidean distance between the raw chromatograms, the Pearson correlation of the smoothed chromatograms, the number of fractions separating the maximal values of each chromatogram, and the smallest Euclidean distance between any pair of fitted Gaussians. PrInCE concatenates features from each replicate but processes each isotope channel separately, providing a total of eighteen features to two naive Bayes classifiers. Importantly, unlike other published approaches, all features provided to the classifier are derived solely from the data and do not incorporate prior biological knowledge, providing greater power for novel interaction discovery [38].

In addition to the features calculated from the co-fractionation data, the naive Bayes classifier requires sets of true positive (TP) and false positive (FP) interactions as training data. Previously, we have observed that many known protein-protein interactions cataloged in literature-curated databases are highly assay- or context-specific, and developed a “universal gold standard” subset of the CORUM database [28] tailored to predicting interactions from co-fractionation data [40]. This subset was used to train the classifier, with intra-complex interactions considered true positives and inter-complex interactions considered true negatives, using tenfold cross-validation and taking the medium of all folds as the final interaction score. The naive Bayes classifier assigns an interaction probability to every pair and returns a ranked list with putative interactions ordered by their interaction probability. The precision of the network was calculated at each point in the ranked list as the ratio of true positives to true positives and true negatives among interactions at that probability or higher, and we retained control and stimulated networks at 70% precision.

### Validation of the biological relevance of the IFN interactome

To validate the biological relevance of the protein-protein interaction networks recovered by PrInCE, we assembled an aggregate network including unique interactions found in either the medium or heavy isotopologue channels at 70% precision and compared the aggregate network to randomly rewired networks using a degree-preserving algorithm as described above. Human Gene Ontology annotations [103] were obtained from the UniProt-GOA database [104] and processed with the R package “ontologyIndex” [105]. Annotations with the evidence codes “IPI” (inferred from physical interaction), “IEA” (inferred from electronic annotation), “NAS” (non-traceable author statement), or “ND” (no biological data), or the qualifier “NOT,” were removed, and annotations were propagated up the GO hierarchy. Very broad GO terms (annotated to more than 100 proteins) were removed, and the total proportion of interacting protein pairs sharing at least one GO term in each ontological category (biological process, cellular compartment, molecular function) was calculated for both rewired and observed networks. The distribution of interacting pairs sharing at least one GO term in each category among randomized networks was used to calculate an empirical *p* value for the observed enrichment. The same procedure was followed to evaluate the tendency of interacting protein pairs to be associated with the same disease, using disease genes obtained from the Mouse Genome Database [106] and mapped to their human orthologs using InParanoid [48]. The tendency of interacting protein pairs to contain domains known to physically interact in a high-resolution three-dimensional structure was similarly assessed using domain-domain interactions obtained from the 3did database [39], with Pfam domain annotations obtained from UniProt [96]. Co-expression of protein pairs was calculated using the tissue proteome dataset described by [107]. Phylogenetic profiles were constructed using the InParanoid database ([48], with the similarity in phylogenetic profile of a protein pair defined as the Pearson correlation between the binary presence/absence vectors of each protein across all species [108]. To quantify the proportion of novel interactions in the stimulated vs. unstimulated conditions, known interactions were compiled from eighteen databases, including BIND [109], BioGRID [110], CORUM [28], DIP [111], HINT [112], HIPPIE [113], HPRD [114], IID [115], InBioMap [19], InnateDB [41], MatrixDB [116], Mentha [117], MINT [118], MPPI [119], NetPath [120], PINA [121], Reactome [122], and WikiPathways [123]. Only human interactions were retained, and self-interactions were excluded. From pathway databases and InnateDB, only the subset of information cataloging physical PPIs was retained. Gene and protein identifiers used to catalog interactions in these databases were mapped to UniProt accessions using identifier mapping files distributed by UniProt. Differential expression results from meta-analyses of 103 diseases were obtained from the MetaSignature web application (http://metasignature.stanford.edu/) [42, 43]*.* To specifically evaluate the relevance of IFN stimulation to mapping interactions for genes dysregulated at the transcriptional level in these diseases, we compared the likelihood of genes that were upregulated at a 1% FDR participating in interactions detected only after IFN stimulation as compared to all other interactions detected by SEC-PCP-SILAC, and to the complete set of literature-curated human interactions, and assessed statistical significance using a *z* test of the log-odds ratio.

### Functional analysis of the IFN interactome

To quantify functional differences between the IFN-stimulated and unstimulated interactomes, we developed a permutation-based statistical test at the network level (Fig. S7A). Briefly, for each term in the Gene Ontology, we identified all proteins annotated with that term, then calculated the total number of interactions in the stimulated and unstimulated networks between these proteins, and the difference between the two networks. We then randomly rewired both control and stimulated networks 1000 times using a degree-preserving algorithm and calculated the difference in randomized networks. The null distribution of the randomized networks was used to calculate a *z* score, which was subsequently converted to a probability and adjusted for multiple hypothesis testing using the method of Benjamini and Hochberg. To compare the enriched GO terms identified by this approach to those based on the protein content of the stimulated and unstimulated networks, we additionally calculated, for each GO term, the odds of proteins in each network being annotated with that term, and tested for enrichment using the *z* score of the log-odds ratio. GO terms with statistically significant differences between stimulated and unstimulated networks at 10% FDR were visualized as an enrichment map [52] using the “ggnetwork” package [124] and the Fruchterman–Reingold layout algorithm [125], as implemented in “igraph.” Edges were drawn between GO terms on the basis of the complete set of proteins each was annotated to if the Jaccard index was greater than 0.33. For select terms, we additionally plotted the distribution of differences in number of interactions in randomized networks as a histogram.

Autocorrelations were calculated as the Pearson correlation between pairs of stimulated and unstimulated chromatograms, normalized to *z* scores, and aggregated across all three replicates using Stouffer’s method. dN/dS ratios between human and mouse genes were obtained from Ensembl BioMart [126]. The total proportion of species in which an ortholog of each gene was present was quantified using the InParanoid database [48]. pLI scores were obtained from ExAC [49] and quantile normalized. Partial Spearman’s correlations between autocorrelation and dN/dS, phylogenetic breadth, and pLI, controlling for log_2_-fold change as observed in the shotgun proteomics dataset, were calculated using the R package “ppcor” [127]. To assess whether the observed rank correlations were sensitive to outliers at the level of the SEC-PCP-SILAC chromatograms, we additionally calculated the Spearman correlation after sampling with replacement from the raw chromatograms 1000 times, and recalculating the autocorrelation *z* score between stimulated and unstimulated protein correlation profiles.

### Evolutionary analysis of IFN-stimulated genes

Core, conserved, and species-specific ISGs were obtained from Shaw et al. [7], on the basis of upregulation in all vertebrates studied, in human and at least one other species, and in human only, respectively. The statistical significance of the difference between distributions of Pearson’s correlations of raw PCP chromatograms in the unstimulated and stimulated conditions was assessed using a Brunner–Munzel test; correlations based on fewer than five pairwise observations were excluded from analysis. Co-immunoprecipitations from stimulated and unstimulated cells were compared using limma [98], and enrichment for different classes ISGs in immunoprecipitations of stimulated cells was assessed by gene set enrichment analysis [128], as implemented in the R package “fgsea” [129].

### Analysis of siRPL28 knockdown data

Time-course transcriptome profiles of mouse ISGs in CD19^+^ B lymphocytes were obtained from [8]. These profiles were normalized by maximum expression across all timepoints, as in the original analysis; mapped to their human orthologs using InParanoid [48]; and matched to proteins that were differentially expressed after 4 h or 24 h of IFN stimulation in our shotgun proteomics data. Analysis of the siRPL28 SILAC proteomics dataset required proteins to be quantified in both the heavy and medium channels in at least two of three replicates. Protein ratios were log-transformed and compared between siRPL28-treated and untreated, IFN-stimulated cells using the moderated *t* test implemented in limma [98]. A consensus set of ISGs was defined as those upregulated in human and at least four other species (five of ten species total) in the Shaw et al. dataset, although the observed enrichment was insensitive to the precise number of species used to define this set. The enrichment for ISGs among up- or downregulated proteins was assessed by gene set enrichment analysis [128], as implemented in the R package “fgsea” [129]. The abundance profiles of selected ISGs were plotted after normalization by subtraction of mean abundance and division by the standard deviation.

## Supplementary information

**Additional file 1.** Supplementary figures S1-S10.

**Additional file 2.** Shotgun proteomic SILAC ratios of IFN-stimulated cells at 4 h or 24 h IFN treatment.

**Additional file 3.** GO term enrichment on significantly changed SILAC ratios of IFN-stimulated cells at 4 h or 24 h IFN treatment.

**Additional file 4.** SEC-PCP-SILAC interactomes of unstimulated and IFN-stimulated HeLa cells at 70% precision.

**Additional file 5.** Representation of known CORUM protein complexes in the IFN interactome.

**Additional file 6.** Autocorrelation z scores between pairs of stimulated and unstimulated chromatograms.

**Additional file 7.** Gene Ontology terms differentially enriched between unstimulated and IFN-stimulated interactomes.

**Additional file 8.** Immunoprecipitation-mass spectrometry of four core ISGs (IFI35, STAT1, NMI, and PNPT1) in IFN-stimulated and unstimulated cells.

**Additional file 9.** Label-free quantitation intensities of ribosomal proteins in monosomes and polysomes.

**Additional file 10.** Shotgun proteomic SILAC ratios of siRPL28-treated cells plus IFN for 8 h.

**Additional file 11.** Gene set enrichment analysis of SILAC ratios in siRPL28-treated and IFN-stimulated cells compared to untreated controls.

**Additional file 12.** Label-free proteomics of IFN-stimulated cells treated with siRNAs targeting RPL28, RPS26, RPS28, or control siRNAs.

**Additional file 13.** Review History.

## Data Availability

The mass spectrometry proteomics data have been deposited to the ProteomeXchange Consortium [130] via the PRIDE partner repository [131] with the dataset identifier PXD013809 [132]. In addition, processed chromatograms for each replicate have been deposited to the EMBL–EBI BioStudies database [133], with accession S-BSST254 [134].
