# Supplementary material for: Dynamic rewiring of the human interactome by interferon signaling
Source: Genome Biol. 2020 Jun 15;21:140. doi: 10.1186/s13059-020-02050-y (PMC7294662; doi:10.1186/s13059-020-02050-y)
Supplement: Supplementary file 1 — Additional file 1. Supplementary figures S1-S10. [file 13059_2020_2050_MOESM1_ESM.pdf]

Supplementary figures

Dynamic rewiring of the human interactome by interferon signaling

Craig H. Kerr[1,2,3,4], Michael A. Skinnider[1,3], Daniel D.T. Andrews[2], Angel M. Madero[1], Queenie W.T. Chan[1], R. Greg Stacey[1], Nikolay Stoynov[1], Eric Jan[2], Leonard J. Foster[1,2]*

[1] Michael Smith Laboratories, University of British Columbia, Vancouver BC, V6T 1Z4

[2] Department of Biochemistry and Molecular Biology, University of British Columbia, Vancouver BC, V6T 1Z3

[3] These authors contributed equally

[4] Current address: Department of Genetics, Stanford University, Stanford, CA 94305, USA

* Correspondence: foster@msl.ubc.ca

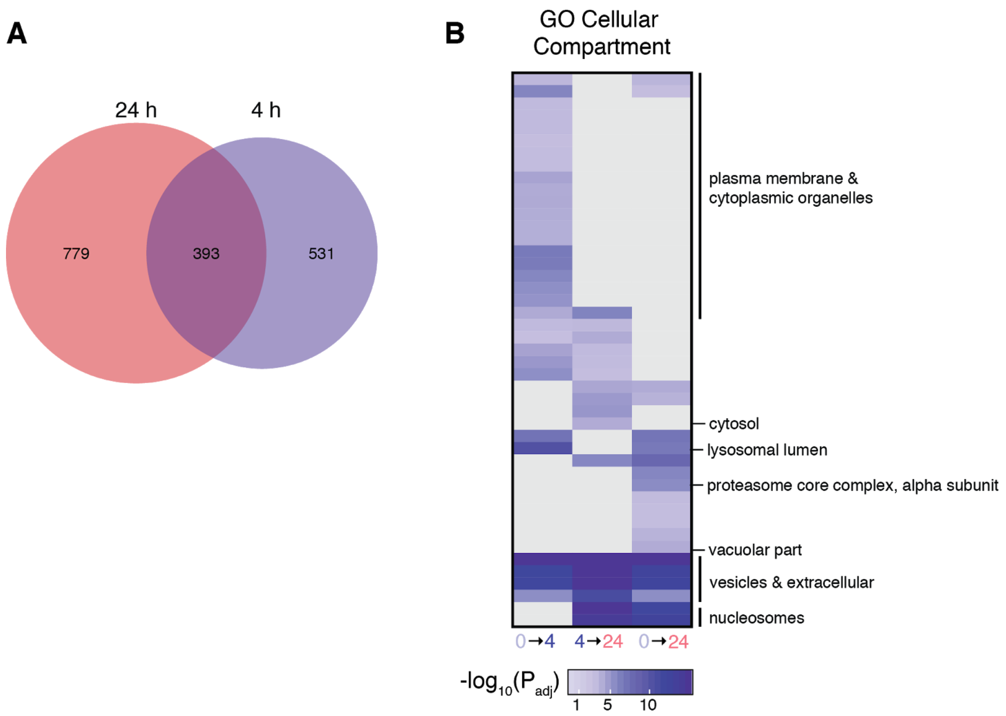

Fig S1. Proteomic characterization of the type I IFN response.

(A) Venn diagram of overlap between differentially expressed proteins after 4 h and 24 h of IFNβ stimulation, relative to unstimulated cells.

(B) Gene Ontology (GO) terms for cellular compartments significantly enriched among differentially expressed proteins after 4 h or 24 h of IFNβ stimulation.

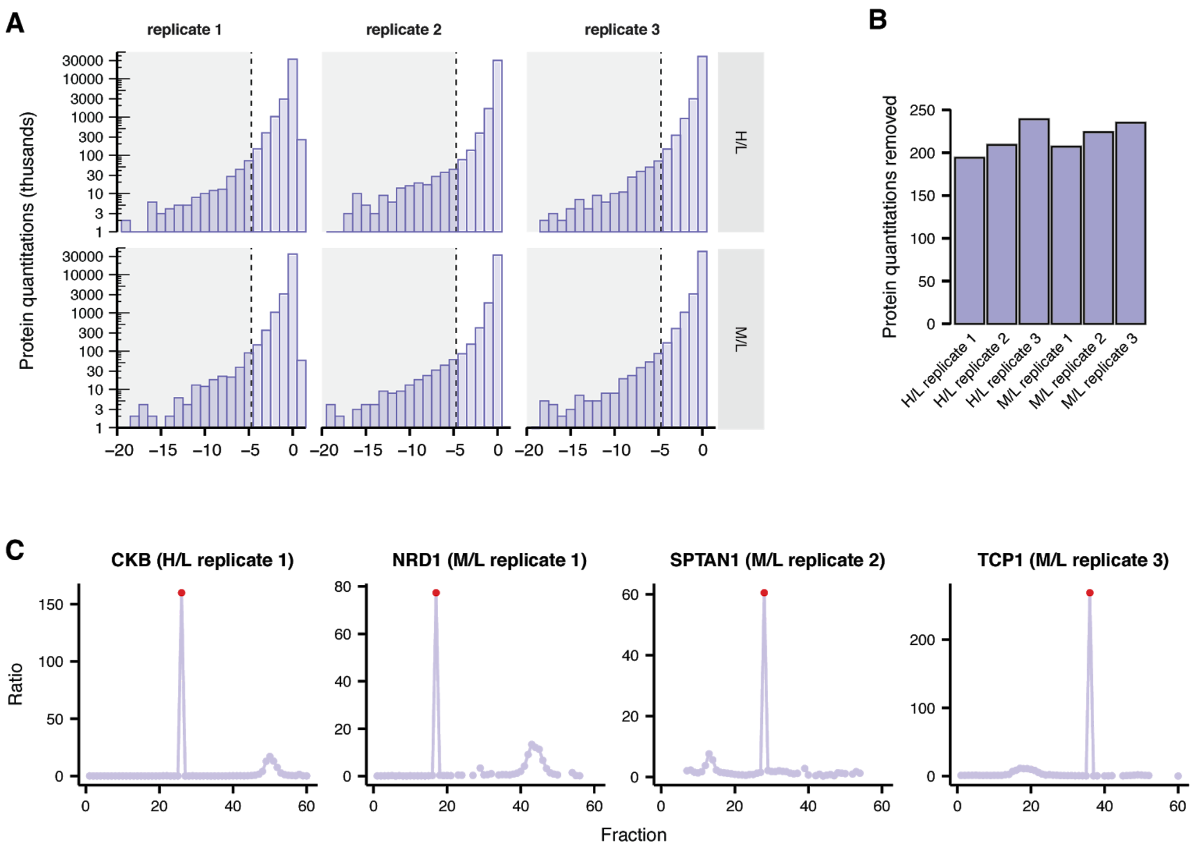

Fig S2. Removal of high-magnitude errors in protein quantitation prior to protein-protein interaction network inference by MODERN.

(A) Distribution of z scores for 245,841 total protein quantifications across three biological replicates, as determined by MODERN (Methods). Dashed lines and shaded areas show z score thresholds corresponding to a two-tailed family-wise error rate of 0.05 in each replicate, given the total number of points observed.

(B) Total number of protein quantitations censored in each biological replicate, based on an autocorrelation z score corresponding to a two-tailed family-wise error rate of 0.05, given the total number of points observed.

(C) Examples of high-magnitude errors in protein quantitation correctly detected by MODERN.

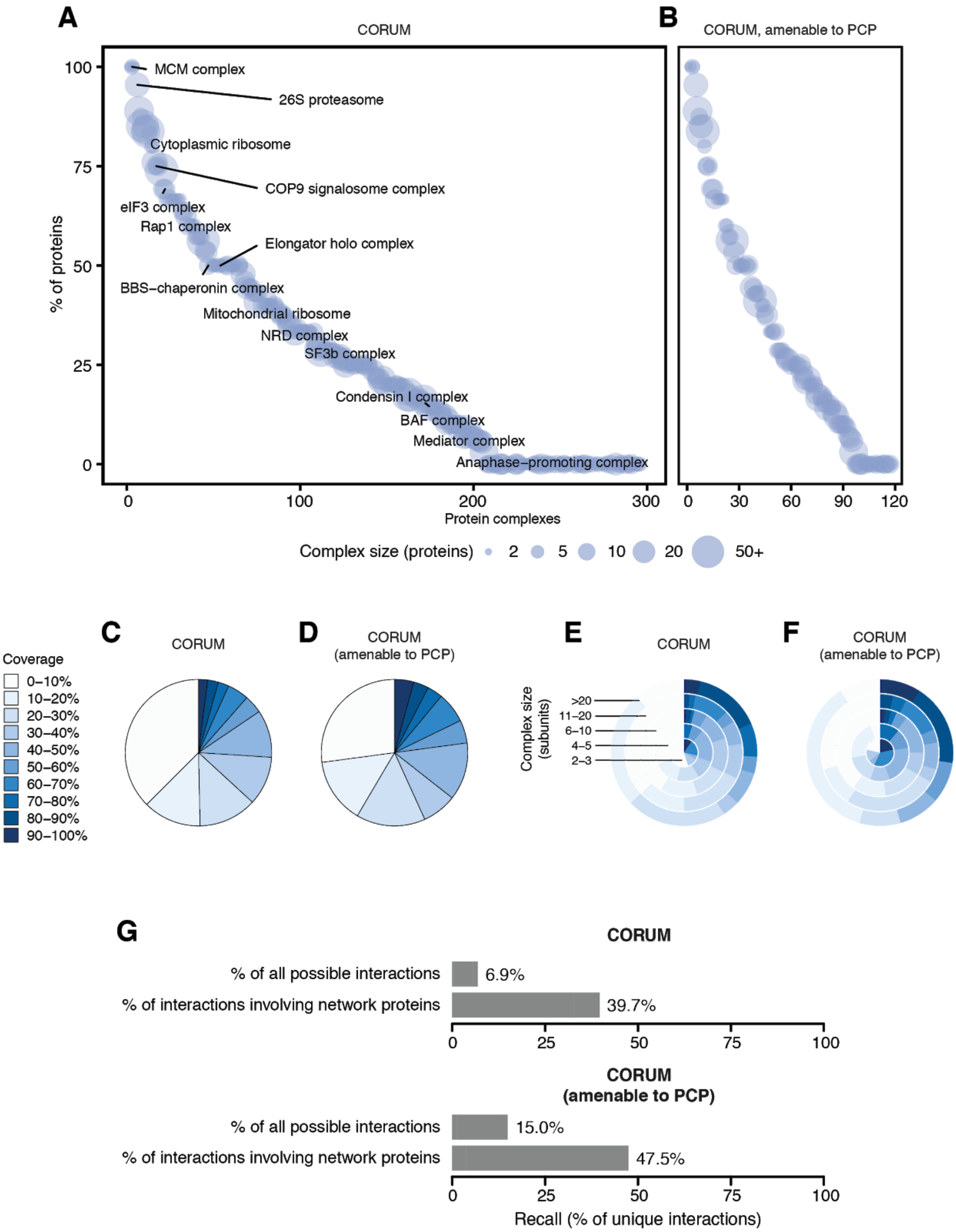

Fig S3. Recovery of known protein complexes in the IFN interactome.

(A-B) Proportion of subunits from individual CORUM protein complexes represented in the IFN interactome, for all of CORUM (A), with select complexes highlighted, or the subset previously found to be amenable to detection by PCP [38], (B).

(C-D) Proportion of CORUM protein complex subunits detected for all CORUM complexes, (C), or the subset

amenable to detection by PCP, (D).

(E-F) As in (C-D), but stratified by protein complex size (number of subunits).

(G) Recall of co-complex interactions between CORUM complex subunits, between all known subunits or the subset of subunits detected in the network, for either all of CORUM, top, or the subset amenable to detection by PCP, bottom.

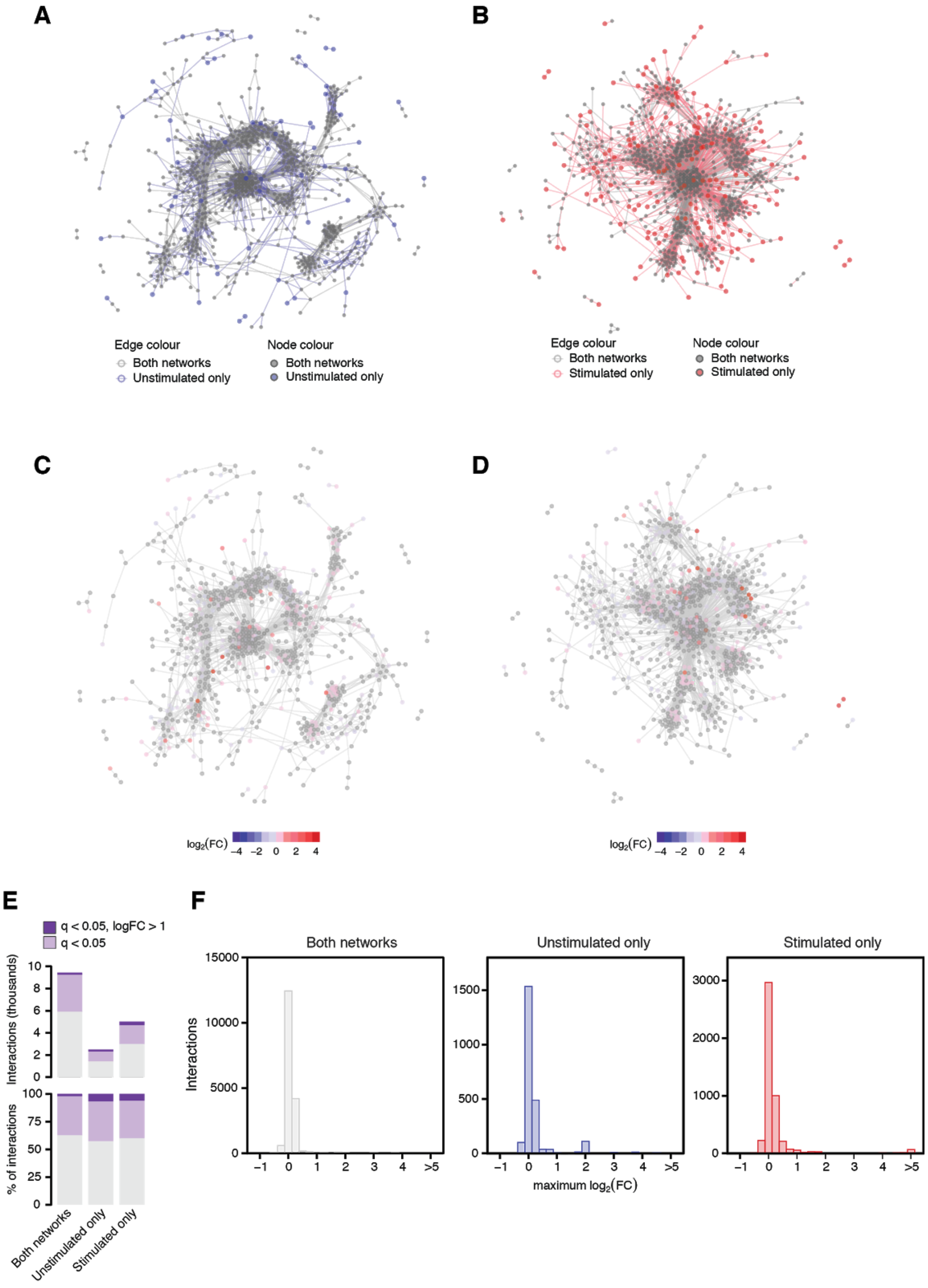

Fig S4. Visualization of the unstimulated and IFN-stimulated interactomes.

(A-B) Network diagrams of the protein-protein interaction networks detected by SEC-PCP-SILAC in unstimulated (A) and IFN-stimulated (B) cells.

(C-D) As in (A-B), but with proteins colored by their log_2-fold change at 24 h, as detected by SILAC shotgun

proteomics.

(E) Top, number of interactions in the unstimulated and IFN-stimulated network involving a protein for which a statistically significant change in abundance was detected at 24 h by SILAC shotgun proteomics at 5% FDR, and the subset of these for which a twofold or greater change in abundance was detected, as in Fig 3E. Bottom, as in top, but plotting the proportion of interactions in each category instead of the absolute number.

(F) Distribution of maximum $\log_2$-fold change values among interacting protein pairs detected in both networks, unstimulated cells only, or IFN-stimulated cells only.

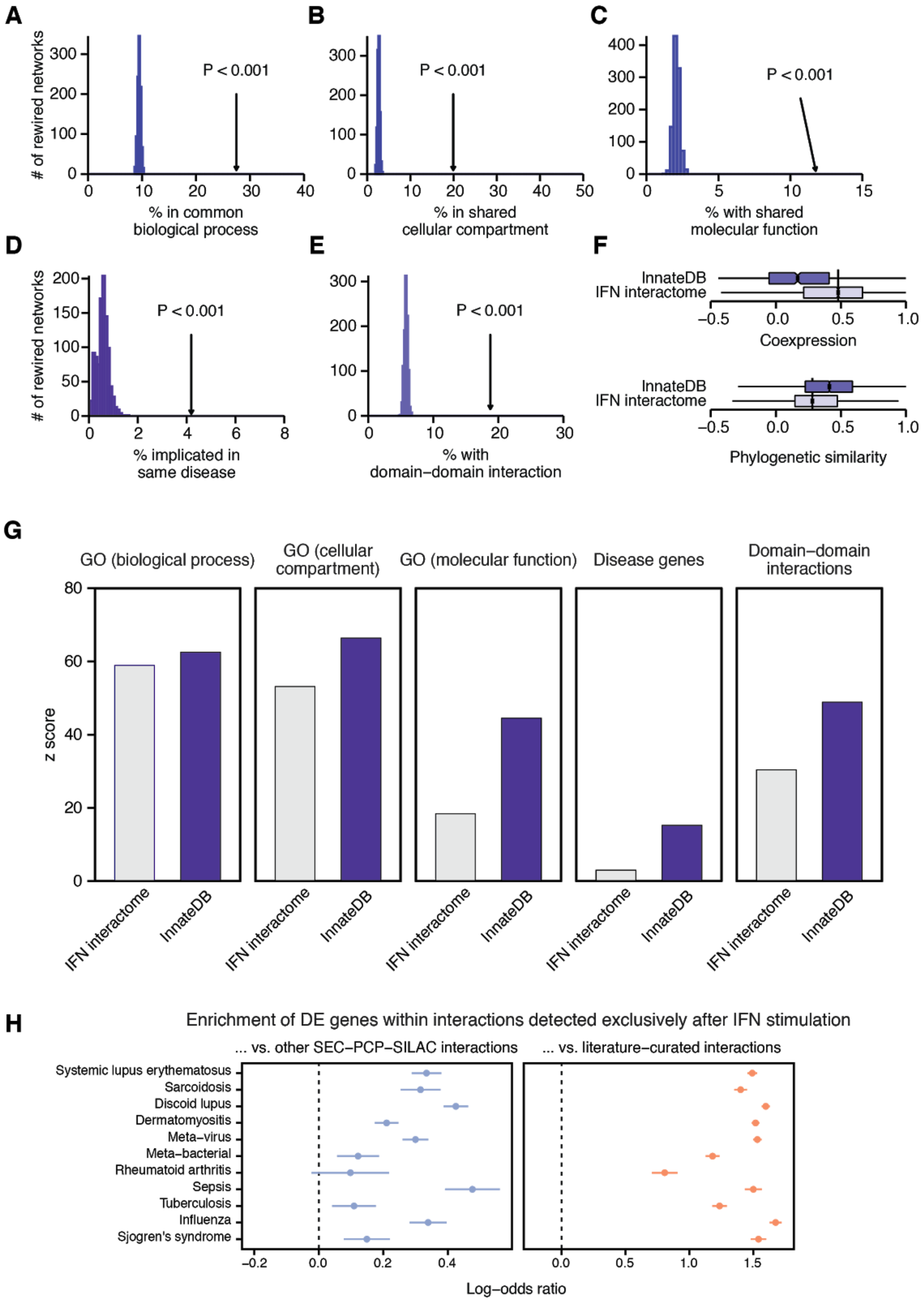

Fig S5. Comparison of the IFN interactome to literature-curated interactions associated with the innate immune response.

(A-C) Proportion of interacting protein pairs sharing at least one biological process (A), cellular compartment (B), or molecular function (C) Gene Ontology term in the InnateDB database, arrow, or 1,000 randomly rewired networks, histogram.

(D) Proportion of interacting protein pairs implicated in the same disease in the InnateDB database, arrow, or 1,000

randomly rewired networks, histogram.

(E) Proportion of interacting protein pairs supported by a domain-domain interaction in the InnateDB database, arrow, or 1,000 randomly rewired networks, histogram.

(F) Pearson correlations reflecting protein abundance and phylogenetic profile similarity between interacting protein pairs in the IFN interactome or the InnateDB database.

(G) Enrichment for shared Gene Ontology terms, disease gene annotations, or domain-domain interactions in the IFN interactome and the InnateDB database, shown as z scores relative to 1,000 randomly rewired networks.

(H) Odds ratio of IFN-specific interactions involving a gene product upregulated in eleven diseases characterized by an elevated IFN transcriptional signature, relative to all other interactions detected by SEC-PCP-SILAC, left, or literature-curated interactions recorded in eighteen databases, right.

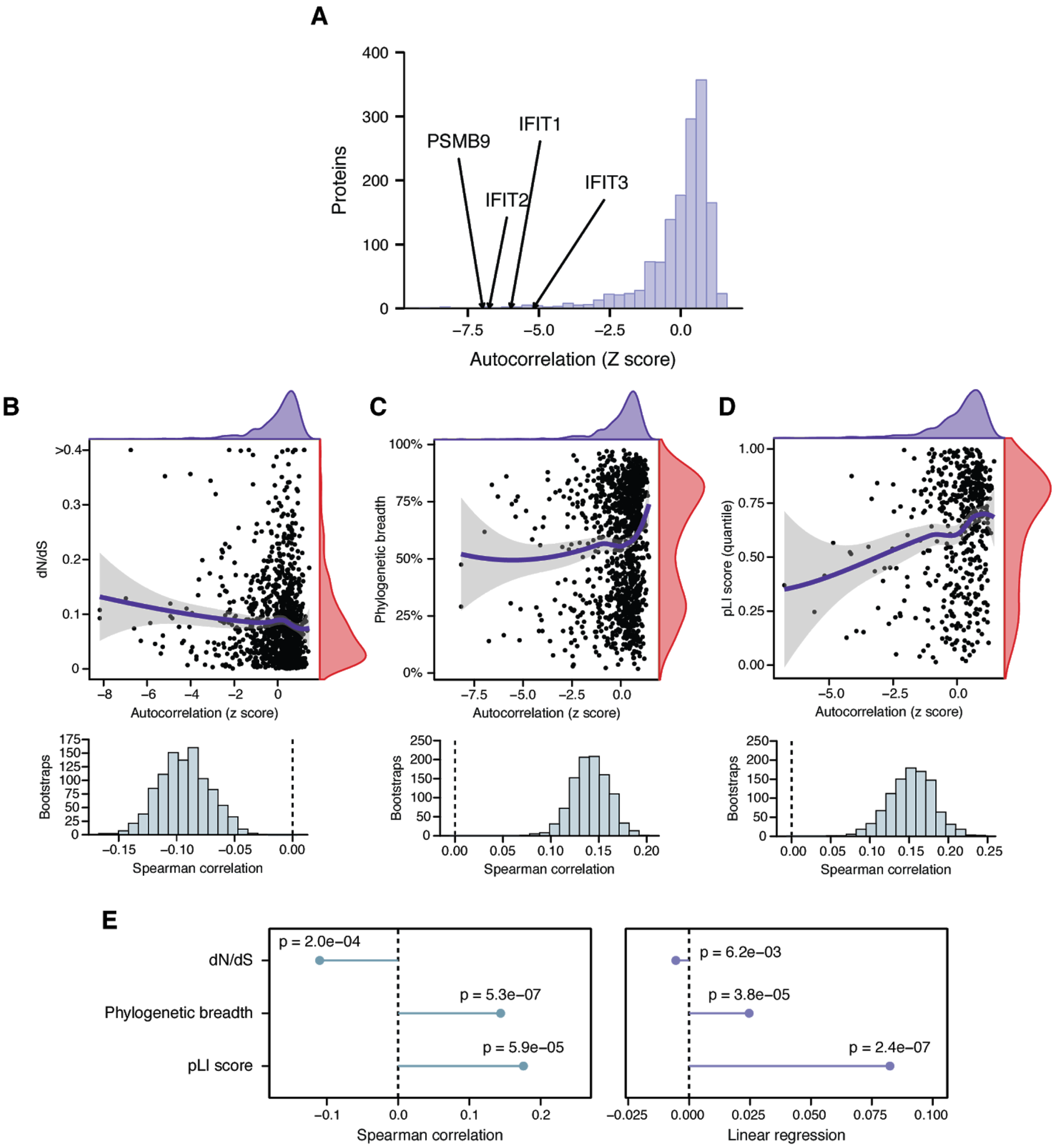

Fig S6. Evolutionary plasticity of the interactome response to IFN stimulation.

(A) Distribution of autocorrelation z scores between IFN-stimulated and unstimulated protein correlation profiles, derived from Stouffer integration across all three replicates. Selected highly rewired proteins are highlighted.

(B) Top, relationship between autocorrelation z score and ratio of nonsynonymous to synonymous substitutions (dN/dS; (Spearman $\rho = -0.11$, $p = 1.9 \times 10^{-4}$). Bottom, distribution of Spearman correlation coefficients when sampling with replacement from SEC-PCP-SILAC chromatograms and re-calculating the autocorrelation z score between IFN-stimulated and unstimulated protein correlation profiles.

(C) Top, relationship between autocorrelation z score and proportion of genomes in the InParanoid database [47] in which an ortholog of the protein was present $\rho = 0.14$, $p = 5.3 \times 10^{-7}$). Bottom, distribution of Spearman correlation coefficients when sampling with replacement from SEC-PCP-SILAC chromatograms and re-calculating the

autocorrelation z score between IFN-stimulated and unstimulated protein correlation profiles.

(D) Relationship between autocorrelation z score and pLI score quantile (Spearman $\rho = 0.18$, $p = 5.9 \times 10^{-5}$). Bottom, distribution of Spearman correlation coefficients when sampling with replacement from SEC-PCP-SILAC chromatograms and re-calculating the autocorrelation z score between IFN-stimulated and unstimulated protein correlation profiles.

(E) Spearman correlation coefficients, left, and linear regression coefficients, right, between the autocorrelation z score and ratio of nonsynonymous to synonymous substitutions (dN/dS), the proportion of genomes in the InParanoid database in which an ortholog of the protein was present (phylogenetic breadth), and the pLI score quantile (pLI).

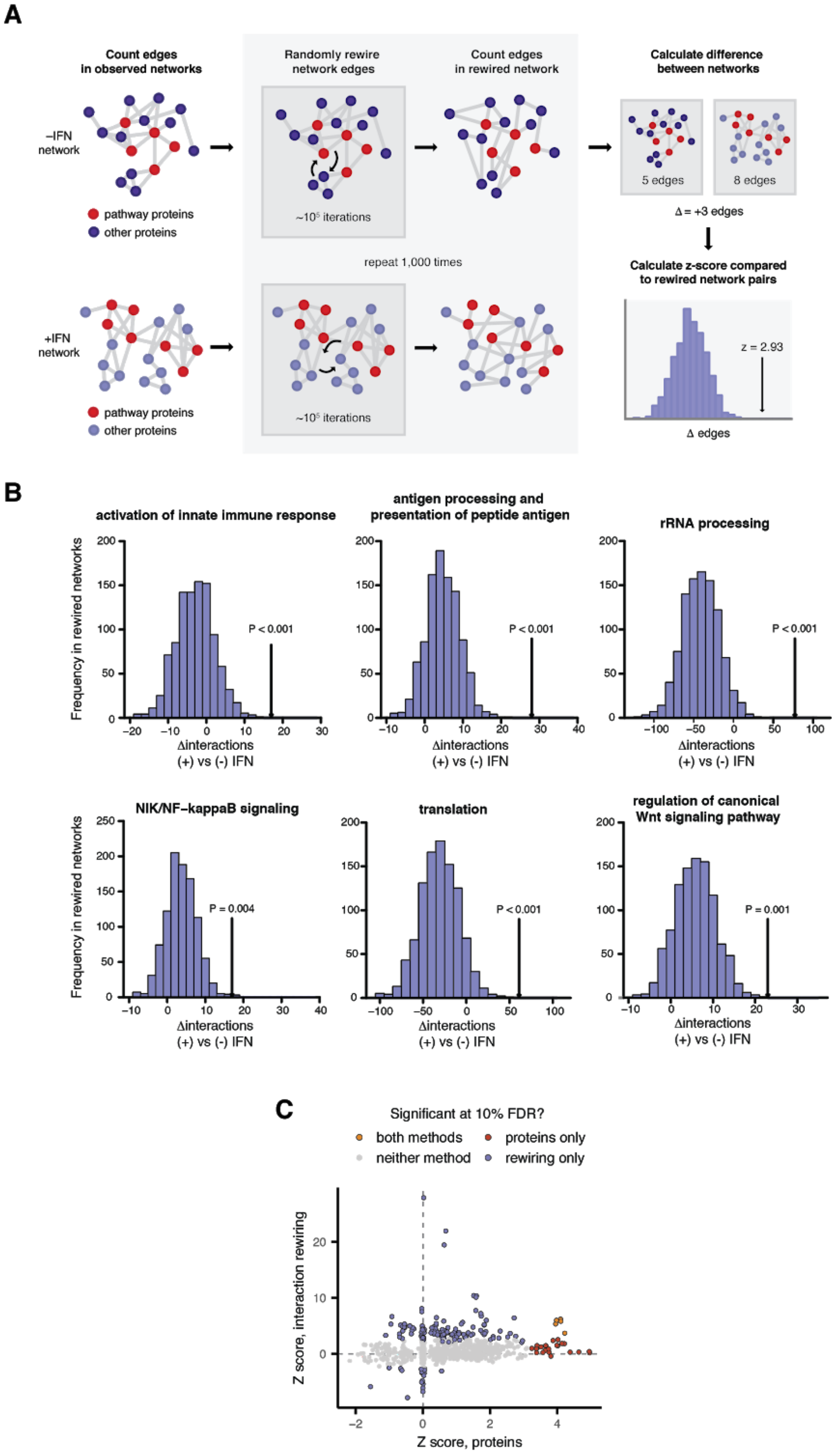

Fig S7. A statistical framework for differential network analysis at the functional level.

(A) Schematic overview of the workflow for functional differential network analysis (Methods). For a given Gene Ontology (GO), the total number of interactions between proteins annotated with that term was calculated in

stimulated and unstimulated networks. The difference in the number of interactions involving that term between conditions was then compared to the difference between 1,000 randomly rewired versions of the same networks.
(B) Examples of GO terms significantly enriched in the IFN-stimulated network. Histograms show the difference in the number of interactions between 1,000 randomly rewired versions of the IFN-stimulated and unstimulated networks; arrows show the observed difference in the number of interactions.
(C) Comparison of z scores obtained through the differential network analysis method, as shown in panel (A), and the z score from an odds ratio test on protein membership in each network.

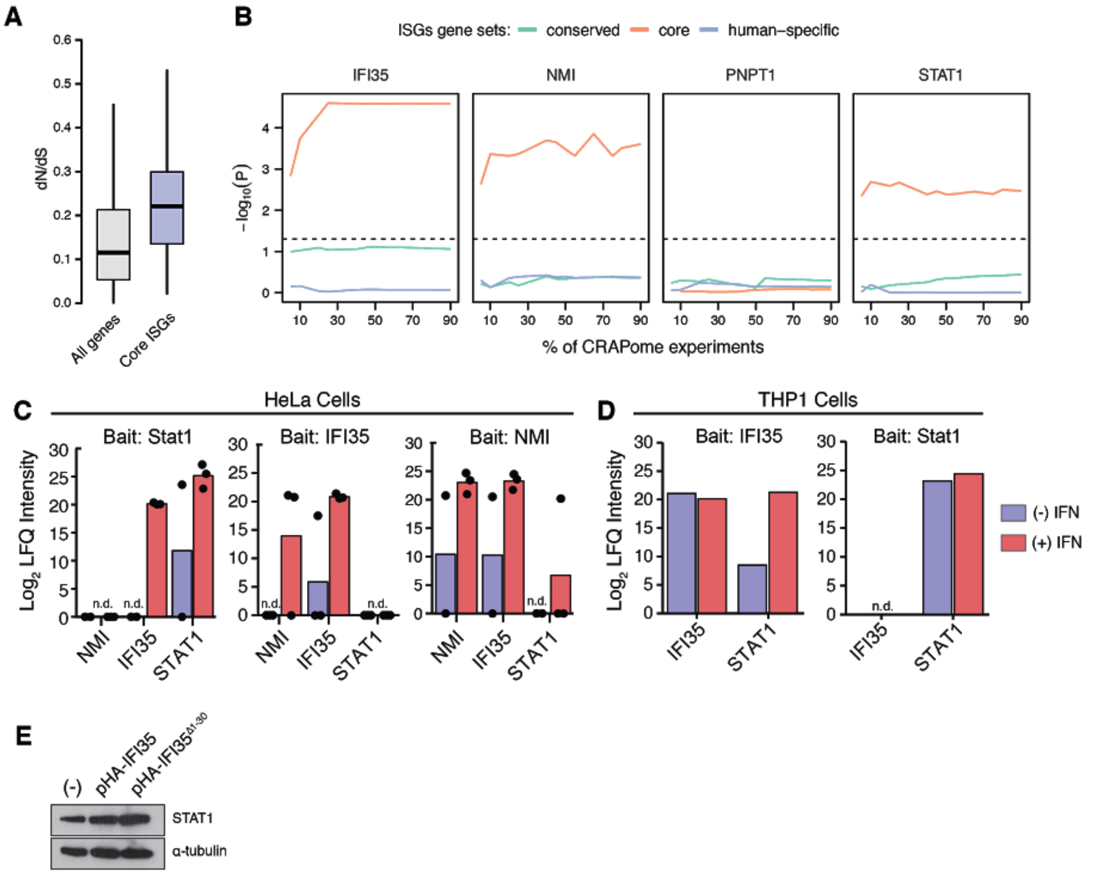

Fig S8. IFN induces interactions between rapidly evolving genes with a conserved role in the IFN response ("core ISGs").

(A) Ratio of nonsynonymous to synonymous substitutions (dN/dS) between human and mouse for 'core ISGs', induced in response to IFN stimulation in ten vertebrate species, and all other human genes.

(B) Gene set enrichment analysis p-values for core, conserved, and human-specific ISG products in comparisons of immunoprecipitations of IFI35, NMI, PNPT1, and STAT1 from IFN-stimulated or unstimulated cells, after removal of potential nonspecific interactors found in between 5% and 95% of human pulldowns in the CRAPome database.

(C) Abundance of co-immunoprecipitated proteins and baits (STAT1, IFI35, or NMI), normalized relative to an IgG control, in IFN-stimulated or unstimulated HeLa cells by label-free quantification (LFQ). N = 3 for (+) IFN, N = 2 for (-) IFN, n.d. = not detected.

(D) Abundance of co-immunoprecipitated proteins and baits (STAT1, IFI35, or NMI), normalized to an IgG control, in IFN-stimulated or unstimulated THP1 cells by label-free quantification (LFQ). N = 2 for both conditions, n.d. = not detected.

(E) Western blot of endogenous STAT1 upon IFN stimulation alongside overexpression of wildtype or truncated IFI35.

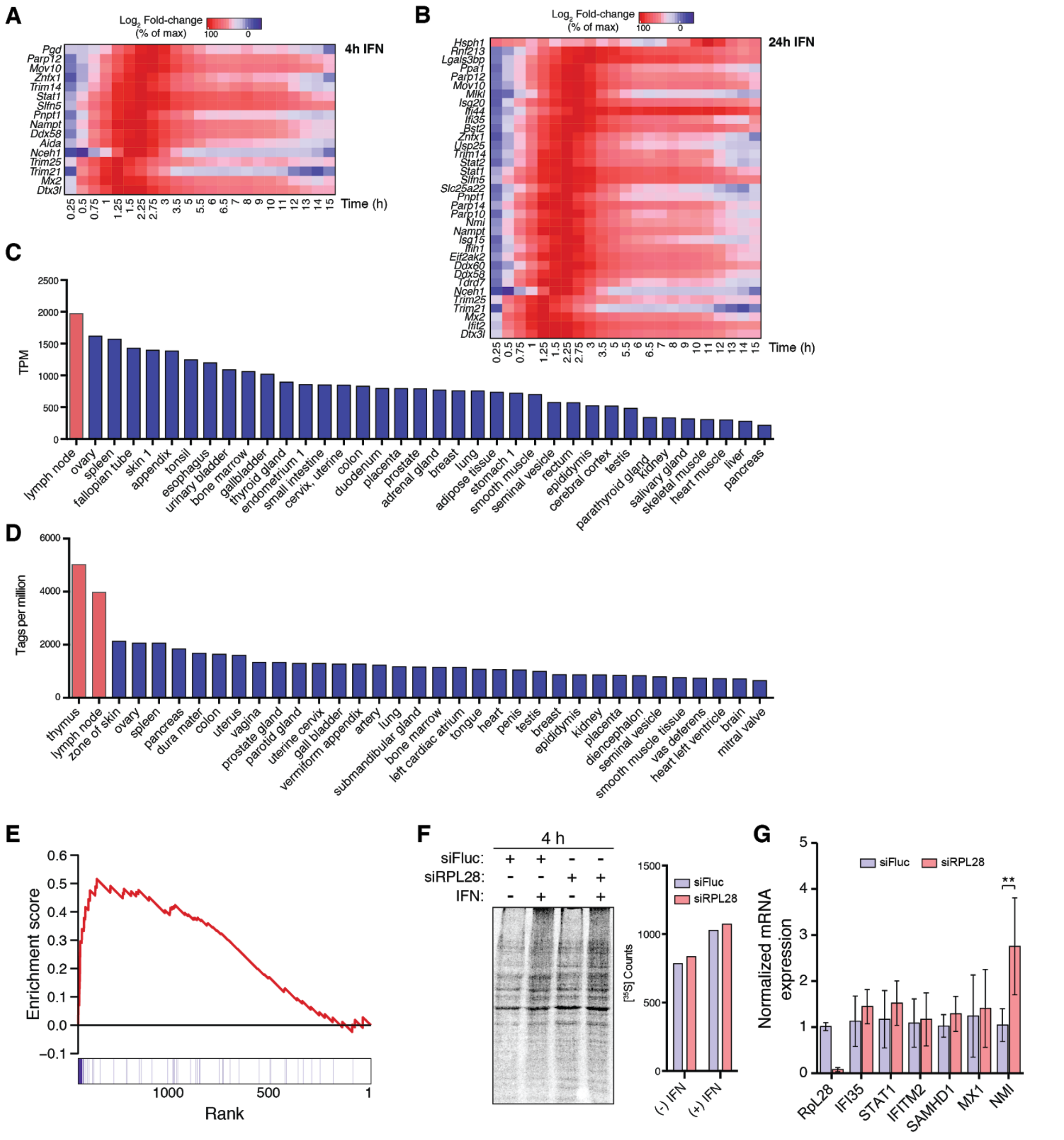

Fig S9. RPL28 buffers the translation of interferon-stimulated genes and is not essential for ribosome function.

(A–B) mRNA expression profiles of proteins that were significantly upregulated at the proteomic level after 4 h (A) or 24 h (B) of IFN stimulation in fine-grained time-course data [8].

(C) RPL28 mRNA expression in human tissues from the Human Protein Atlas [68].

(D) RPL28 mRNA expression in top 35 human tissues from the FANTOM5 database.

(E) Gene set enrichment analysis enrichment score, top, and barcode plot [132], bottom, showing ranks of ISGs in a comparison of siRPL28-treated and IFN-stimulated cells compared to untreated controls.

(F) Metabolic labeling of HeLa cells by [^{35}S]-Met/Cys incorporation after siRNA knockdown of RPL28 for 48 h followed by IFN stimulation. Left: representative gel of [^{35}S]-Met/Cys labeled proteins at 4 h post-IFN treatment. Right: average quantitation of [^{35}S] counts in precipitated protein from two independent experiments at 8 h post-IFN treatment.
(G) qRT-PCR of various ISGs from cells treated with siRPL28 or control siRNA for 48 h followed by IFN treatment for 8 h. Expression levels were normalized to *TUBB* then to control. ** $P < 0.005$.

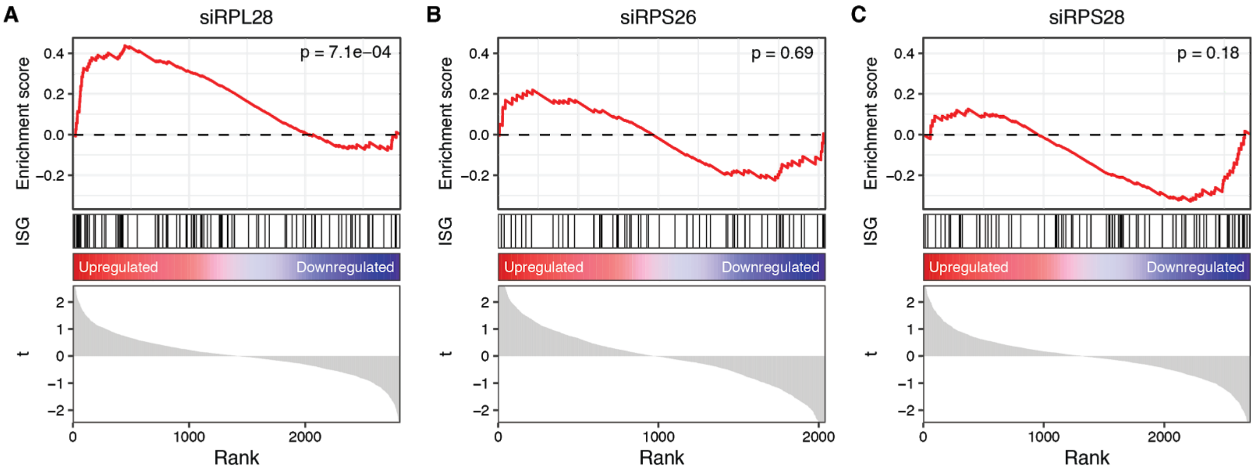

Fig S10. Increased abundance of interferon-stimulated genes is specific to RPL28 knockdown.
Gene set enrichment analysis barcode plots showing ranks of ISGs in a comparison of cells treated with IFN-stimulated cells and siRNAs targeting RPL28 (A), RPS26 (B), and RPS28 (C), compared to cells treated with control siRNA.
