# Supplementary material for: Dynamic rewiring of the human interactome by interferon signaling
Source: Genome Biol. 2020 Jun 15;21:140. doi: 10.1186/s13059-020-02050-y (PMC7294662; doi:10.1186/s13059-020-02050-y)
Supplement: Supplementary file 13 — Additional file 13. Review History. [file 13059_2020_2050_MOESM13_ESM.docx]

**Review History**

**Reviewer 1:**

Major Comments:

This is an interesting, relatively thorough and well-written paper in which the authors have used protein correlation profiling (PCP) coupled with SILAC to construct a map of the interactome in HeLa cells in response to stimulation with IFNB. Very few studies to date have investigated network dynamics so this study is an important advance for the field.

The finding that RPL28 regulates the protein expression of ISGs was also particularly interesting and is an important finding.

As discussed by the authors PCP has a number of advantages over more traditional approaches to map PPIs (AP-MS/Y2H), most notably its higher throughput which makes assessment of interactome dynamics more feasible. That said, the method has its limitations and my most serious concern is that authors should temper their language (particularly in the title and abstract) wrt the fact that PPIs can only be inferred using this approach. I discussed the methodology used with several colleagues who are experts in PPI mapping and all agreed that this method could infer co-complex membership but is insufficient evidence to support that these are direct PPIs. No doubt, many of the PPIs are direct and the authors do a good job in showing that their inferred interactions are enriched for direct PPIs. Many of the interactions however are likely indirect associations in the same complex. Some more careful use of language (e.g. using terms like "inferred PPIs") throughout the manuscript should resolve this concern.

My other major comment is that I was surprised given the many different analyses conducted by the authors that actual comparisons of the IFN- and IFN+ networks was relatively limited. It would be valuable to compare these networks in more ways (visually, topologically, etc) and to assess what are the likely drivers of the "rewiring". For example, is rewiring predominantly driven by proteins that were not expressed or lowly expressed in unstimulated cells that are now highly expressed following stimulation or is something more sophisticated going on? Are there specific nodes in the network that are particularly rewired? How many node/edges are shared vs rewired in the two networks? Can only present/absent rewiring be assessed or can more quantitative changes also be considered (e.g. a node that is present in both networks but expressed at significantly different levels). Can the protein expression data be overlayed on the networks. What does this reveal? Can enrichment analysis be performed simply on the list of proteins present in the IFN- network and compared to the IFN+ network? How does this compare to the more complicated approach presented in Fig S4?

More Minor (but important) Comments:
Please more fully discuss the use of HeLa cells in these experiments, particularly given that HeLa cells are deficient in RIG-I responsiveness. How applicable are the findings likely to be to other cell types.
Pg 4 Line 42: Please state in the main text what IFN was used to stimulate cells. Different IFNs would presumably have very different effects.
Pg 5 Line 9. It is mentioned that IFN signalling induces changes to cellular metabolism. Can you be more specific - cellular metabolism is very broad. Also many other processes were also significantly enriched, please make it clearer that it was not just cellular metabolism. Similarly for the results of the data at 24hrs.
The analysis presented in Figure 1F-G is unclear to me. The methods state that "the likelihood of the observed number of interactions between differentially expressed proteins was evaluated by randomly rewiring the InBioMap interactome 100 times using a degree-preserving algorithm". I follow how the observed # of interactions is calculated but not how this is compared to random expectation? It seems that some critical detail is not explained. What if the same number of proteins (as were DE) were randomly selected from InBioMap 100 times to assess if number of interactions for DE proteins is surprising or not?
There are more DE proteins at 24hr than 4hrs. Isn't it then surprising that there are fewer observed interactions at 24hrs?
Since the modern algorithm is currently unpublished and this is critical to the analysis can this software please be made available.
Figure 2E A more thorough comparison of how the data compares to Corum would be valuable. What proportion of Corum complexes are found? Are all the components of recovered Corum complexes found - if not what proportion tends to be found in the data.
Pg 7 Line 34. Can you be more specific wrt how many interactions were novel and clarify in the main text how novelty was assessed?
Pg 8 the correlations between autocorrelation and evolutionary rates such as dn/ds are far from convincing. I was not convinced that this analysis added much to the paper.
Pg 10 Given that STAT1 does not pull down IFI35, isn't it just as possible that the coil-coiled domain of IFI35 is required for a direct interaction with another protein (possibly NMI) and the influence on STAT1 activity is indirectly mediated by an interaction with one or more proteins other than STAT1?
Table S1 the 4th sheet in the file is labelled "Statistical Analysis - 8h +IFN" - I assume this should be "Statistical Analysis - 24h +IFN". Hopefully this is just a labelling error?
Table S1. In the 3rd sheet many of the adjusted P-values have exactly the same value (to 9 decimal places). This is probably because a rounded value for the unadjusted P-values were used in the correction algorithm. This is unlikely to change anything - it just looks unusual.

**Reviewer 2:**

In the paper "Dynamic rewiring of the human interactomes by interferon signalling" authors employ size exclusion chromatography coupled with MS to study the changes in protein-protein interactions 24 hours after type I Interferon stimulation. By applying PrInCE method, the authors predict 27694 binary interactions at 70% precision.
The paper is nicely written and the figures are of high quality. The rich interaction dataset, such as the one presented here, would be of high relevance for understanding the molecular mechanisms of immunity given the interactions are true and reliable. Many observations derived from the data are quite speculative and not sufficiently substantiated by proper controls or validation experiments at this point. However, the manuscript definitely holds a lot of promise and could be very interesting to the broader field of biologists.

Major points:
1.     The measurement of IFN-induced changes to SEC-profiles takes 24h after the treatment. Even though I do understand that 4h (very earls) IFN treatment does not elicit major protein abundance changes 24h time point is a quite late time point. It is expected that, in addition to immediate changes to IFN-signalling, protein abundance and interactome changes will propagate to multiple downstream pathways. This notion is supported by the broad range of GO terms enriched at 24h post treatment. While also interesting, these changes are not necessarily specific to immune response. In particular, at this time point it's expected to observe downregulation of a some proteins, disassembly of corresponding protein complexes and negative regulatory processes - thus the data becomes very difficult to interpret given that multiple processes are operating in parallel. The authors should clearly comment on that in the text.

2.     The authors use the term "interaction rewiring" or "remodeling" without clearly defining it. Given the significant changes to the protein abundance, it's expected that the abundance of the related protein complexes would be also changing. E. g. IFN-induced changes to immunoproteasome SEC-profile shown on Figure 2B (what proteins were used?) could be explained solely by the regulation of the proteasome subunits abundance, whereas the interaction affinities (which the term "rewiring" implies) may stay unchanged (the same peaks observed in stimulated and unstimulated conditions). Did the authors regarded such cases as IFN-specific interactions or required that the SEC peak positions and shapes were significantly changed? In some extreme cases (e.g. protein is exclusively expressed upon interferon stimulation) SEC profile may only be available in one condition. While both affinity- and abundance-caused changes are interesting and biologically relevant, they imply different mechanisms and affect downstream analysis (e.g. Figures 3H, 3I, S3, Figure 4), therefore should clearly be deciphered by the type of change and be annotated in Table S3.

3.     Surprisingly, the central part of the study, the SEC-PCP-MS dataset itself and the derived interactome, are not sufficiently represented in the figures and supplementary data. It would be very beneficial to provide some visualization of the network, so that its global topology, as well as the topology of the proposed IFN-specific subnetwork, could be observed by the readers (Figure 1E doesn't allow that). Similarly, the network data provided in Table S3 is too scarce, since it only provides the final precision scores. To facilitate the use of their dataset in follow-up bioinformatic studies, the authors should at least provide the table with all the features used by their naive Bayes classifier for each protein group, as well as the autocorrelation z-score, which seem to correlate with interaction rewiring upon IFN stimulation.

4.     When assessing the quality of their IFN interactome (Page 6), the authors only discuss the precision component. They should also provide the percentage of known interactions they recall at 70% precision level. This is critical information since it gives the reader confidence in the data. It would be particularly interesting to check precision for their IFN-stimulated network. Adding InnateDB (https://www.innatedb.com) to the broad list of public interaction databases they are already considering should make this comparison more relevant - particularly since this database contains interaction data in stimulated or infected conditions. Naturally, when calculating the precision of binary interactions, the estimates are biased by big complexes (since the number of interactions grows as n2, where n is the number of proteins in a complex). To overcome this, the authors should weight the interactions by the sizes of molecular complexes (e.g. using 1/M as the weight of each binary interaction, where M is the molecular weight of the putative protein complex deduced from the corresponding peak of the SEC-PCP profile) or providing separate statistics for complexes of different sizes.

5.     When commenting on the relevance of the IFN interactome, the authors show that it's represented among the genes implicated in IFN-related diseases (Page 7, Figure 3I). While this observation suggests that the interactome is not random, it doesn't necessary prove that it extends our understanding of molecular mechanisms over the existing knowledge. To show that, the authors would need to compare the enrichment of novel interactions over the already published ones, and/or show that this enrichment is specific to immunity-related diseases over the unrelated ones. Likewise, showing the estimates for the already known global interactome on Figures 3B-3F, as well as adding its coexpression and phylogenetic similarity profiles to Figure 3G, should provide an important reference for assessing the IFN interactome results.

6.     The authors apply regression analysis to show that SEC-profile changes upon type I IFN stimulation seem to positively correlate with the rate of protein evolution (Page 8, Figures S3B-S3D). It's not clear from the methods whether the SEC-profile autocorrelation z-score was corrected for protein abundance changes to exclude that the observed effect is merely due to the upregulation of interferon-stimulated genes (ISGs). One way to address this is to show that the observations still hold when the regression analysis is limited to ISGs only. The authors highlight IFIT proteins as one of the top rewired proteins. Indeed, it's published that IFITs form the heterodimer complex upon IFN stimulation, so it is very interesting whether, on top of the dramatic upregulation, the authors could see the corresponding peak shift in their SEC-MS profiles, or whether it's so low-abundant in non-stimulated conditions that they don't detect any peaks at all.

7.     The reviewer had difficulties assessing the relevance of the hypothesis that evolutionary conserved ISGs ("core ISGs") are more engaging in physical interactions than the less conserved ones (page 9). The authors didn't provide the list of the proteins which they considered for each of those classes, neither they provided the underlying data for Figure 5C (validation of "core ISGs" hypothesis by AP-MS experiments with STAT1, IFI35 and NMI baits). In any case, so far this hypothesis is supported only by the data obtained from HeLa cells, which represent <25% of proteins (2590 protein groups) of one particular (and severely altered) human tissue. It's known that the repertoire of immunity-related proteins and their regulation vary dramatically across different cell types, so "human-only" and "conserved" ISGs may be more engaged in interactions in the other cell types. The authors also have to rule out the alternative explanations for the enrichment they observe (Figure 5A), e.g. whether "core ISGs" coincide with the particular biological process (GO BP), molecular function etc. Does the hypothesis still hold when, instead of profile correlations, the authors test for the number of ISG-specific interactions they predict? If Figure 5C shows the ranks of enrichment in IFN+ vs IFN- IPs of IFI35, NMI and STAT1, is it corrected for protein abundance changes of corresponding genes upon IFN stimulation? Does the figure only show the specific interactors of the corresponding baits?

8.     Essentially, the authors put forward two different evolutionary hypotheses: one that evolutionary plasticity results in more rewiring, the other one is that rewiring is more observed for evolutionary conserved ISGs. Perhaps, the authors should propose one united hypothesis, since combining these two together seems nontrivial.

9.     Negative regulation of STAT1 by IFI35 was shown by Shirai et al in astrocytes (https://www.ncbi.nlm.nih.gov/pubmed/28109979/). IFI35 mediated degradation of RIG-I results in reduced IFNb cytokine expression. The reporter assay shown here indicates that treatment with recombinant IFNb affects gene expression under control of ISRE. This is surprising since it implies functions downstream of IFNb signaling. If the authors claim the same mechanism of action (degradation), they should show degradation of STATs through IFI35. The STAT1-dependent transcription experiment (Figure 5G) misses controls. The authors should show that the removal of 1-30 residues of IFI35 doesn't change its localization, and perform co-IP experiments to validate the interaction of the coiled-coil domain of IFI35 and coiled-coil domain of STAT1. The authors suggest that the interaction of STAT1 and IFI35 may be substoichiometric, does that mean that their SEC-PCP-MS can predict transient interactions? The peaks of IFI35, NMI and STAT1 in IFN-stimulated condition seem to be quite abundant (Figure 5B).

10.     The authors show that the knock-down of RPL28 leads to upregulation of ISGs. However, the observed effects are minor and most of the observed changes are not statistically significant, raising the question of biological relevance of the observed knock-down effect. Further experiments are required to confirm the hypothesis of RPL28 being the selective negative regulator of ISGs translation, as it's still possible that the observed abundance changes might be a by-product of another process, or be downstream of e.g. STAT1 regulation. Perhaps, the authors should explore their proteome data for the alternative explanations (e.g. GO term enrichment analysis) as the volcano plot suggests there is a number of proteins that are up- and down-regulated upon siRPL28 treatment.

Minor points:
1.     Page 5. Line 34 (Figures 1F,1G): It's not clear why protein interaction network randomization was used to test whether IFN-affected proteins are enriched with pairwise interactions: essentially it tests a weaker hypothesis, i.e. whether human interactome and the set of IFN-affected genes are both non-random. It looks like the appropriate test for the original hypothesis would be to keep the interactome intact and randomize just the set of proteins. Even more relevant would be to check whether the observed enrichment in interactions is specific to IFN stimulation in comparison to the other stimuli. To check this the authors may consider MSigDB C2 CGP sets (http://software.broadinstitute.org/gsea/msigdb/genesets.jsp?collection=CGP).

2.     Figure 2D. Adding secondary X axis with the approximate molecular weight would be beneficial.

3.     Page 6. L12. The text refers to 2590 protein groups identified, whereas Figure 2D mentions 1520 as the "complete set" of PCP profiles. What is the reason for this discrepancy?

4.     Methods. Page 23 L19. The authors integrate quite a number of protein interaction databases. However, InnateDB (https://www.innatedb.com/), which would be particularly relevant for estimating the number of novel IFN-specific interactions is not included.

5.     Page 23. L60 Shawl et al (2017) Reference number missing (should be [7]).

6.     Page 29. Figure 5A. Is "Control" the same as "Unstimulated"? If so, it should be called so for consistency. Also, the colors should be used consistently (Figure 3I uses reversed palette)

7.     Page 29. Figure 5D. Does the middle panel show the same data as the lower one, just without STAT1 profile? Is so, perhaps it should be removed to avoid confusion. Are the shown profiles the average of 3 replicates?

8.     Page 31. Figure 6E Legend. "yea"?

9.     Page 34. Figure S2: Y axis should read: "Protein quantifications (thousands)". Using Log10 scale for Y axis would make the plot more informative. What does the dashed line denote?

10.     Page 35. Figures S3B-S3D: It's not clear which method was used to obtain the trend line and confidence intervals. The marginal density plots suggests that the distributions are not normal (including the autocorrelation z-score). In such cases applying linear regression is problematic, since the results could be biased by the outliers. The authors may consider transforming the metrics (e.g. by log-transform), so that the distribution of the transformed data is more appropriate for linear regression.

11.     References to the "modern" method (e.g. Figure S2A, S2C legend). The term "modern method" is confusing as it might be not clear that it refers to the new version of PrInCE package. Perhaps the authors may consider using the more specific name for the method or, at least, using captital letter and quotes ("Modern" method).

12.     Table S2: It would be nice to have a column listing the names or accession codes of the enriched genes. E.g. 17 genes from the "viral entry into the host cell" are upregulated at 4h, so it would be nice to know their identities.

13.     Table S4: Column with the names of enriched genes would be helpful as well as the column with ontology (BP/MF/CC).

**Authors’ response to reviews:**

**Reviewer #1**

This is an interesting, relatively thorough and well-written paper in which the authors have used

protein correlation profiling (PCP) coupled with SILAC to construct a map of the interactome in HeLa

cells in response to stimulation with IFNB. Very few studies to date have investigated network

dynamics so this study is an important advance for the field.

The finding that RPL28 regulates the protein expression of ISGs was also particularly interesting and

is an important finding.

We thank the reviewer for their positive comments on our work.

As discussed by the authors PCP has a number of advantages over more traditional approaches to

map PPIs (AP-MS/Y2H), most notably its higher throughput which makes assessment of interactome

dynamics more feasible. That said, the method has its limitations and my most serious concern is that

authors should temper their language (particularly in the and abstract) wrt the fact that PPIs can only

be inferred using this approach. I discussed the methodology used with several colleagues who are

experts in PPI mapping and all agreed that this method could infer co-complex membership but is

insufficient evidence to support that these are direct PPIs. No doubt, many of the PPIs are direct and

the authors do a good job in showing that their inferred interactions are enriched for direct PPIs. Many

of the interactions however are likely indirect associations in the same complex. Some more careful

use of language (e.g. using terms like "inferred PPIs") throughout the manuscript should resolve this

concern.

The reviewer brings up two important points in this comment. The first is regarding the type of

interactions detected by PCP. Some authors have drawn a distinction between so-called ‘binary’ and

‘co-complex’ interactions. The former, detected by techniques such as yeast two-hybrid (Y2H) or

protein complementation assay (PCA), ostensibly represent direct biophysical contacts. In contrast,

the latter type of interactions are detected by methods such as AP-MS or the protein correlation

profiling method employed here, and represent all pairs of proteins in the same protein complex as

physically interacting. For this reason, experimental methods that detect co-complex interactions tend

to produce much more densely connected interaction networks. Because the presence of all subunits

of a protein complex is typically required for assembly and function, as evidenced by the findings that

protein complex subunits are enriched among dosage-sensitive genes and rarely undergo gene

duplication (Papp et al., *Nature* 2003), we would argue that the co-complex model of interactions is, at

least, no *less* biologically relevant than the binary model. Nonetheless, we agree with the reviewer

that it is important to be clear on the type of interactions detected in this study and the potential

limitations of the co-fractionation family of methods for interactome mapping, and have revised the

Discussion section accordingly. Furthermore, we recognize that we were imprecise with our use of the

term ‘binary interaction’ in the original manuscript, and have removed improper usage of this

terminology.

A second point raised in this comment is that the protein-protein interactions detected in our study are

‘inferred’ and, as such, require further independent confirmation. We fully agree with this point. Our

study employed a relatively lenient precision cutoff (70%), and we believe our results show the

resulting networks can represent a useful resource to the community for discovery, as well as for

systems-level investigation. However, we absolutely agree that all of the putatively novel interactions

detected here will need to be confirmed by an orthogonal method. This is not a unique limitation of

SEC-PCP-SILAC, or co-fractionation approaches more broadly, though: past experience indicates this

is also true for large-scale AP-MS or Y2H studies, and indeed any high-throughput interactome

mapping method.

We added the following paragraph to the Discussion section to address these two points:

On the other hand, several limitations of SEC-PCP-SILAC, and co-fractionation approaches more

generally, must be noted. Although SEC-PCP-SILAC provides a basis for inference of co-complex

membership based on correlated protein abundance across conditions designed to separate protein

complexes based on their size, it does not provide direct evidence of physical protein-protein interactions

as such. Moreover, many authors have drawn a distinction between methods that detect ‘binary’

interactions, which ostensibly represent direct biophysical contacts between protein pairs, and those that

detect ‘co-complex’ interactions, which represent all pairs of proteins in the same protein complex as

physically interacting [81, 82]. Whether one type of interaction is more biologically meaningful than

another has been a matter of some debate, and it is noteworthy that many literature-curated interaction

databases and standardized formats for molecular interaction data do not draw such a distinction [83–85].

Nonetheless, the fact that not all of the interacting protein pairs reported by PrInCE will be in direct

biophysical contact can also be viewed as a limitation of the resource presented here, although some

evidence exists that direct contacts can also be recovered from the underlying protein correlation profiles

using more bespoke methods [86]. A more general limitation is that our study employed a relatively lenient

precision threshold of 70%. While the results presented here indicate the IFN interactome has value as a

resource for discovery, and for systems-level interrogation of the innate immune response, any of the

individual putatively novel interactions detected here requires independent confirmation by an orthogonal

experimental method to be considered reliable, as is the case for any high-throughput interactome

mapping technique.

My other major comment is that I was surprised given the many different analyses conducted by the

authors that actual comparisons of the IFN- and IFN+ networks was relatively limited. It would be

valuable to compare these networks in more ways (visually, topologically, etc) and to assess what are

the likely drivers of the "rewiring". For example, is rewiring predominantly driven by proteins that were

not expressed or lowly expressed in unstimulated cells that are now highly expressed following

stimulation or is something more sophisticated going on? Are there specific nodes in the network that

are particularly rewired? How many node/edges are shared vs rewired in the two networks? Can only

present/absent rewiring be assessed or can more quantitative changes also be considered (e.g. a

node that is present in both networks but expressed at significantly different levels). Can the protein

expression data be overlayed on the networks. What does this reveal? Can enrichment analysis be

performed simply on the list of proteins present in the IFN- network and compared to the IFN+

network? How does this compare to the more complicated approach presented in Fig S4?

Both reviewers expressed a desire to visualize the networks, appreciate the overall degree of

interactome rewiring more quantitatively, and understand the factors that likely drive rewiring. To

address the questions the reviewer raises, we have added a new row to Figure 3, and a new

supplementary figure (Figure S4) to the revised manuscript. Briefly, we find that between 21–35% of

interactions, but only 7–16% of proteins, are specific to one of the two networks. Although >6,500

interactions involve a differentially expressed protein, the vast majority of these proteins undergo only

subtle (that is, less than two-fold) changes in abundance by 24 h. A much smaller subset of

interactions ( *n* = 652) involve at least one protein with a larger (that is, twofold or greater) difference in

abundance in IFN-stimulated cells; these interactions are significantly overrepresented among the

condition-specific interactions detected in our study. We interpret these results to suggest that while

subtle changes in protein abundance may act to ‘fine-tune’ interactions, e.g. through competition for a

binding interface, abundance alone cannot fully account for the observed interactome rewiring,

suggesting a role for other cellular mechanisms.

The Results section has been revised to detail these new analyses as follows:

Among the 26,361 protein-protein interactions detected overall, 11,924 were identified in

unstimulated cells and 14,437 in IFN-stimulated cells, with 9,434 detected under both conditions (Figure

3B and Figure S4A-B). Thus, 71.6% of all detected interactions were identified in both networks. This

rewiring could not be attributed solely to the variable protein composition of the two networks, as a smaller

proportion of protein nodes themselves were specific to one of the two networks (Figure 3C). To

investigate the degree to which changes in protein abundance in response to IFN stimulation could

underlie the observed rewiring, we overlaid our shotgun proteomics data (at the 24 h timepoint; Figure 1C)

onto the IFN-stimulated and unstimulated networks (Figure S4C-D and Table S3). A total of 6,562 unique

interactions involved at least one protein with a statistically significant difference in abundance upon IFN

stimulation, but most of these proteins exhibited relatively subtle changes in expression (Figure 3D). In

contrast, fewer interactions involved proteins with more dramatic IFN-induced changes in abundance (i.e.

two-fold or greater). Moreover, whereas interactions involving differentially expressed proteins were

equally likely to be observed in either or both of the IFN-stimulated or unstimulated networks (p = 0.20, χ2

test), interactions involving proteins with twofold or greater differences in abundance were significantly

enriched among the set of condition-specific interactions (p < 10–15, χ2 test; Figure S4E). To further

assess this trend, we calculated, for all interactions, the maximum log2-fold change within the interacting

protein pair, and confirmed that only a small fraction of interactions involve proteins with dramatic changes

in abundance (Figure 3E and Figure S4F). Overall, these results suggest that only a small number of

condition-specific interactions involve proteins with pronounced IFN-induced changes in abundance. More

subtle changes in expression affect a larger number of interactions, and might act to ‘fine-tune’

interactions. However, the large number of interactions between proteins that are not differentially

expressed in response to IFN stimulation suggests a role for other mechanisms, such as post-translational

modification, subcellular localization, or protein degradation, in IFN-dependent interactome remodeling.

**Figure 3. Biological relevance of the IFN interactome** *[...]*

(B) Proportion of all detected interactions observed both networks or in one condition only.

(C) Number of interactions in the unstimulated and IFN-stimulated network detected in both networks or in

one condition only.

(D) Number of proteins for which at least one interaction was detected in the unstimulated and

IFN-stimulated network or in one condition only.

(E) Number of interactions in the unstimulated and IFN-stimulated network involving a protein for which a

statistically significant change in abundance was detected at 24 h by SILAC shotgun proteomics at 5%

FDR, and the subset of these for which a twofold or greater change in abundance was detected.

(F) Maximum log 2 -fold change among interacting protein pairs detected in both networks, the unstimulated

network only, or the IFN-stimulated network only.

**Figure S4. Visualization of the unstimulated and IFN-stimulated interactomes.**

(A-B) Network diagrams of the protein-protein interaction networks detected by SEC-PCP-SILAC in

unstimulated (A) and IFN-stimulated (B) cells.

(C-D) As in (A-B), but with proteins colored by their log 2 -fold change at 24 h, as detected by SILAC

shotgun proteomics.

(E) Top, number of interactions in the unstimulated and IFN-stimulated network involving a protein for

which a statistically significant change in abundance was detected at 24 h by SILAC shotgun proteomics

at 5% FDR, and the subset of these for which a twofold or greater change in abundance was detected, as

in Figure 3E. Bottom, as in top, but plotting the proportion of interactions in each category instead of the

absolute number.

(F) Distribution of maximum log 2 -fold change values among interacting protein pairs detected in both

networks, unstimulated cells only, or IFN-stimulated cells only.

The reviewer also expressed a desire to visually compare the two networks. We include a new

supplementary figure, Figure S4, with diagrams of both the stimulated and unstimulated networks

(see above). Furthermore, as requested, we have overlaid the protein expression data onto these

plots, in the form of log 2 -fold changes after IFN stimulation. Unfortunately, these diagrams reflect the

well-known limitation that complex networks with thousands of nodes and edges are difficult to

interpret visually (and consequently are often referred to as “hairballs”, e.g., Flint and Ideker, *PLoS*

*Genet* 2019). To provide a superior resource that will allow users to explore the IFN-induced

interactome network, we have developed an feature-rich web application, available at

http://ifn-interactome.msl.ubc.ca/, that allows users to interactively browse these network diagrams

themselves, as well as to explore the underlying PCP chromatograms. The network view further

allows users to optionally focus either on specific genes of interest, or the sets of genes that are

differentially expressed in meta-analyses of >100 different diseases, including the IFN-related

diseases depicted in Figure 3I. We have added a brief paragraph to the revised Discussion section

introducing this web application:

To facilitate exploration of the complete dataset, we have developed an interactive web application,

available at https://ifn-interactome.msl.ubc.ca, that allows users to visualize both the IFN interactome

network as well as the underlying PCP chromatograms. The network view further allows users to

optionally restrict the network either to specific genes that are of interest, or to entire sets of genes called

as differentially expressed in transcriptomic meta-analyses of 103 different diseases, including the

IFN-related diseases depicted in Figure 3I [41, 42].

During the course of web application development, we updated the PPI networks using a newer

version of the PrInCE algorithm, and found the networks are essentially identical. Nonetheless, we

have regenerated the figures to reflect small changes in the networks (i.e., Figures 2E, 3A-I, 4, and

S7B), as well as the associated supplementary tables.

Finally, as requested by the reviewer, we compared the results of our proposed method for differential

network analysis at the functional level to a simpler enrichment analysis based on proteins

present/absent in each network. Briefly, we calculated an odds ratio for each GO term by comparing

the likelihood that proteins annotated with that term were found in the unstimulated and

IFN-stimulated networks, and compared the z scores of the odds ratios to those obtained from our

rewiring based procedure. The two approaches display little overlap, with only five GO terms called as

statistically significant by both ("nucleic acid binding", "RNA binding", "protein-containing complex",

"organic cyclic compound binding", and "heterocyclic compound binding"). This discordance reflects

the fact that the two procedures test very different null hypotheses. Importantly, by using a

degree-preserving randomization approach (detailed further below), our method explicitly *controls for*

the protein content of each network, since every protein participates in the same number of

interactions in each of the 1,000 randomly permuted networks. This allows our network

rewiring-based approach to specifically test for functional differences in protein-protein interaction

content.

**Figure S7C.** Z scores for GO term functional enrichment between IFN-stimulated and unstimulated

networks using the network rewiring-based approach, y axis, and the protein content of each network, x

axis. A small amount of jitter was added to prevent over-plotting.

We have revised the relevant paragraph of the manuscript as follows:

A total of 341 GO terms were significantly enriched in either stimulated or unstimulated networks at 20 %

FDR, and were visualized as an enrichment map [48] (Figure 4 and Table S 5 ). As expected, we observed

a significant enrichment for interactions between proteins involved in the innate immune response in the

IFN-stimulated network, including GO terms such as ‘antigen processing and presentation’ (Figure 4 and

Figure S7B). The Wnt signalling pathway was likewise enriched in the IFN-stimulated network, consistent

with its link to type I IFN signalling [49, 50] (Figure S7B). Intriguingly, the stimulated network was also

enriched for terms relating to RNA processing and splicing, suggesting a role for physical interactions in

the regulation of alternative splicing during the host response to viral infection [1, 51] . Conversely, the

stimulated network was depleted for interactions involving chromatin remodelling and histone-modifying

machinery, as well as interactions involved in negative regulation of ubiquitination. Surprisingly, we

observed a significant enrichment for interactions related to translation and ribosome biogenesis upon IFN

stimulation (Figure 4 and Figure S7B), potentially accounting for the apparent lag observed in our shotgun

proteomics experiment between ISG transcription and translation. To test whether these enrichments

specifically reflected IFN-induced changes in interactome structure, as opposed to differences in the

protein content of the stimulated and unstimulated networks, we performed a separate functional

enrichment analysis of the proteins found in either network, but found little overlap between GO terms

identified as enriched by interaction rewiring-based and protein content-based methods (Figure S7C).

Collectively, these data reflect wide-ranging functional changes within the human interactome induced by

IFN signalling.

More Minor (but important) Comments:

Please more fully discuss the use of HeLa cells in these experiments, particularly given that HeLa

cells are deficient in RIG-I responsiveness. How applicable are the findings likely to be to other cell

types.

We have included a section in the revised manuscript discussing the use of HeLa cells and how the

findings may relate to other cell types. In particular, we now include additional data demonstrating that

at least one of the interactions detected in HeLa cells is also present in a monocyte-derived cell line,

THP-1, upon IFN stimulation ( Figure S8D ):

**Figure S8.** (D) Abundance of co-immunoprecipitated proteins and baits (STAT1, IFI35, or NMI),

normalized to an IgG control, in IFN-stimulated or unstimulated cells by label-free quantification (LFQ).

Shown is the average of 2 replicates.

We note this in the Results as follows:

Furthermore, the interaction of IFI35 and STAT1 was also detected in a monocyte-derived cell line, THP-1

(Figure S8D), suggesting that this interaction is not limited to HeLa cells.

Regarding the reviewer’s comment on the RIG-I responsiveness of HeLa cells, we are unaware of any

data demonstrating that HeLa cells do not respond to RIG-I agonists. While it is true that Huh7.5 cells

harbour a mutation in RIG-I rendering it defective (Sumpter et al., *J. Virol.* 2005), HeLa cells have

been used in a number of circumstances to study RIG-I signalling in the context of Poly(I:C) treatment

or viral infection (Matsumiya and Stafforini, *Crit. Rev. Immunol.* 2010; Hayakari et al., *PLoS ONE*

2016; Xu et al., *PNAS* 2016; Choi et al., *PNAS* 2009; Yoo et al., *PLoS Pathog.* 2014; Wang et al., *Cell*

*Rep.* 2019). Nonetheless, in our work we treated cells with IFN-β to directly trigger the IFN response,

bypassing the need for signalling through RIG-I. Thus, in the event that the HeLa cells used here are

deficient in RIG-I signalling, it would be unlikely to impact the downstream signalling cascade

triggered by IFN stimulation through the IFN-α/β receptor (IFNAR). We consider these points in the

revised Discussion:

Our differential network map utilized HeLa cells to delineate the impact of IFN stimulation on the human

interactome. HeLa cells have been widely used as a model to understand viral infections and the immune

response, and express core type I IFN pathway components common to most cell types, including the

ubiquitously expressed type I IFN-α/β receptor (IFNAR) [74] and downstream signalling components, such

as JAK1, STAT proteins, and IRF9. Our experimental validation of the IFN-dependent interaction between

STAT1 and IFI35 in a second cell type, the monocyte-derived cell line THP-1, suggests that at least a

subset of the interactions reported here may be conserved across cell types. However, in view of the

variable strength and specificity of the IFN transcriptional response across different cell types [8, 9], it is

likely that a subset of interactions are also cell type-specific, a possibility that warrants further

investigation. Moreover, in this work, we directly stimulated cells with IFN, thus bypassing the need for

cytoplasmic or endosomal sensors to trigger an IFN response. While this provides a direct basis to

understand IFN-dependent interactome remodeling, future studies making use of different activating

ligands, such dsRNA, lipopolysaccharide, other IFNs, or viral infection will be required in order to define

how the interactome of human cells is rewired to establish selective responses to distinct stimuli.

Pg 4 Line 42: Please state in the main text what IFN was used to stimulate cells. Different IFNs would

presumably have very different effects.

We have revised the manuscript to clarify that IFN-β was used in all experiments, both throughout the

introduction and in the first paragraph of the results.

Pg 5 Line 9. It is mentioned that IFN signalling induces changes to cellular metabolism. Can you be

more specific - cellular metabolism is very broad. Also many other processes were also significantly

enriched, please make it clearer that it was not just cellular metabolism. Similarly for the results of the

data at 24hrs.

We have revised the relevant paragraph to (i) include specific examples of enriched specific GO terms

in the main text that were discussed in the original manuscript, and (ii) discuss additional enrichments

relating to more general processes that we observe in our data at both 4 and 24 h, as follows:

Functional enrichment analysis of the proteins that were differentially expressed at 4 h or 24 h, or which

were differentially expressed between the two timepoints, revealed marked temporal differences in the

cellular processes activated by IFN stimulation (Figure 1D–E, Figure S1B and Table S2). Proteins that

were differentially expressed at 4 h were enriched for Gene Ontology (GO) terms related to involvement in

metabolic processes, such as “glycolipid catabolic process” and “regulation of steroid biosynthetic

process”, consistent with the notion that IFN signalling induces changes to cellular metabolism in order to

establish an antiviral state [19, 20] . Other enriched GO terms pointed to a role for cell migration, including

“regulation of macrophage migration” and “cell morphogenesis.” In contrast, the most significantly

enriched GO terms at 24 h were related to chromatin rearrangements : for instance, “DNA conformational

change” and “DNA-replication-dependent nucleosome assembly.” These enrichments are in line with the

finding that IFN stimulation induces chromatin modifications to establish a transcriptional ‘memory’,

resulting in faster and greater transcriptional responses upon restimulation [21] . Enrichment was also

observed for processes such as “posttranscriptional gene silencing,” “gene silencing by RNA,” and “de

novo protein folding,” which may reflect the changing cellular environment after IFN stimulation in

preparation for host defence. Surprisingly, despite the rapid induction of ISG transcription (as early as 30

min post-stimulation; [8] ), we did not observe an enrichment of Gene Ontology (GO) terms related to the

innate immune response until 24 h, reflecting the apparent lag in translation of canonical ISGs (Figure

1B–C).

The analysis presented in Figure 1F-G is unclear to me. The methods state that "the likelihood of the

observed number of interactions between differentially expressed proteins was evaluated by randomly

rewiring the InBioMap interactome 100 times using a degree-preserving algorithm". I follow how the

observed # of interactions is calculated but not how this is compared to random expectation? It seems

that some critical detail is not explained. What if the same number of proteins (as were DE) were

randomly selected from InBioMap 100 times to assess if number of interactions for DE proteins is

surprising or not?

Both reviewers raised questions about the analysis presented in Figure 1F-G, and we apologize that

this was unclear in the original manuscript. We have conducted the suggested analysis, wherein the

number of protein-protein interactions between DE proteins in literature-curated databases is

compared to a null model consisting of random sets of proteins of equal size ( **null 1** ), as opposed to

the null model presented in the original manuscript, which consisted of literature-curated protein

interaction networks rewired using degree preserving randomization ( **null 2** ). Reassuringly, there is an

even more significant enrichment for protein-protein interactions between proteins that are DE in

either comparison.

**Response figure 1.1.** Number of protein-protein interactions in the InBioMap database [22] between

differentially expressed proteins after 24 h (left) of IFNβ stimulation, or between cells stimulated for 4 and

24 h (right), arrows, and between proteins in 100 random sets of proteins of equivalent size, histograms.

However, we have elected not to include this analysis in the revised manuscript. Instead, we have

rewritten the paragraph in question extensively to clarify the motivation underlying the choice of **null**

**2** .

In contemplating this motivation, it is helpful to explicitly state our null hypothesis, which is as follows:

proteins that are differentially expressed in response to IFN stimulation do not interact with one

another any more frequently than we would expect *for a random set of proteins with equivalent*

*topological properties in the known human interactome network* . The importance of controlling for

network topology arises from the fact that in known protein-protein interaction networks, some

proteins participate in hundreds or even thousands of interactions, whereas others participate in only

a handful. Consequently, an arbitrary set of proteins may have an extremely high number of

interactions solely because the set contains a handful of extremely highly connected proteins.

Intuitively, if our sets of DE proteins happened to contain the single most highly connected protein in

the network, a statistically significant result could arise simply because that one protein participates in

many interactions, rather than because the set of DE proteins are highly interconnected with one

another in the protein interaction network as a whole. For this reason, a reasonably large body of

literature has discussed the importance of controlling for network topology, and observed that many

apparently interesting examples of interconnectedness in protein-protein interaction networks can be

attributed solely to the overall number of interactions made by the proteins in question (e.g., Fig. 3 in

the Verleyen *et al.* reference cited in the revised manuscript).

Throughout the manuscript, we were careful to control for this confounding factor, both in our analysis

of the shotgun proteomics data in Figure 1, and in the rewiring-based functional enrichment test

described in Figure S4 (now S7). Notably, the fact that **null 2** is a much more stringent null

hypotheses can be seen by comparing the number of interactions on the x-axis (that is, under the null

hypothesis) in Figure 1F-G and Response figure 1.1 : under **null 1** we expect only ~2,000 interactions

between the proteins DE between 0h and 24 h, but under **null 2** , given their connectivity in the

literature-curated network, we expect to see ~6,500.

To clarify our motivation for the rewired network null, and the procedure used to obtain the null

distribution, we have revised the paragraph in question as follows:

Our attention was drawn to the enrichment for GO terms related to protein-protein interactions after 24 h

of IFN stimulation, including “protein binding,” “protein heterodimerization,” and “protein

heterotetramerization.” We therefore sought to determine whether proteins that were differentially

expressed at 24 h, in comparison to cells stimulated with IFN for 4 h or to unstimulated cells, displayed a

statistically significant tendency to physically interact. To test this hypothesis, we compared the observed

number of protein-protein interactions between differentially expressed proteins in each of the two

comparisons, using the InBio Map database [22] , to the number of interactions observed in networks

rewired using degree-preserving randomization [23]. Importantly, unlike a null model in which an

equivalent number of proteins are drawn at random from the network, this rewiring-based null model

controls for artifactual differences stemming from the degree distribution of the network, whereby a

statistically significant result can reflect the high connectivity of the differentially exposed proteins across

the entire network as opposed to their selective interconnectivity with one another [24, 25]. This analysis

revealed a substantial excess of physical protein-protein interactions between differentially expressed

proteins , relative to random expectation (Figure 1F–G). We compared the strength of this enrichment to

that observed for 3,263 curated gene expression signatures of disparate biological and clinical states

catalogued in the MSigDB “chemical and genetic perturbations” (CGP) collection. Remarkably, proteins

differentially expressed in the 24 h vs. 4 h and 24 h vs. unstimulated comparisons displayed a greater

enrichment for physical interactions than 95% and 97% of CGP signatures, respectively (Figure 1H).

Together, these results led us to hypothesize that, in addition to its effects on ISG transcription, IFN

stimulation induces rewiring of cellular protein-protein interaction networks.

There are more DE proteins at 24hr than 4hrs. Isn't it then surprising that there are fewer observed

interactions at 24hrs?

Although Figure 1F presents known protein-protein interaction between proteins differentially

expressed at 24 h compared to unstimulated (0 h) cells, Figure 1G presents the same statistics

calculated for the 24 h vs. 4 h comparison, not 4 h vs. 0 h. The smaller number of interactions can be

attributed to the fact that fewer proteins are differentially expressed between 24 h/4 h (n = 833

proteins) compared to 24 h/0 h (n = 1,172 proteins). We have revised the paragraph to clarify this

point:

We therefore sought to determine whether proteins that were differentially expressed at 24 h, in

comparison to cells stimulated with IFN for 4 h or to unstimulated cells, displayed a statistically significant

tendency to physically interact.

Since the modern algorithm is currently unpublished and this is critical to the analysis can this

software please be made available.

We apologize for this oversight; the repository had been made public, but the link to the source code

was not included in the original manuscript. The relevant part of the Methods section has been

revised to state:

MODERN is available as an R package from https://github.com/fosterlab/modern.

Figure 2E A more thorough comparison of how the data compares to Corum would be valuable. What

proportion of Corum complexes are found? Are all the components of recovered Corum complexes

found - if not what proportion tends to be found in the data.

Both reviewers requested a more thorough comparison of our networks to known protein complexes

in the CORUM database. We agree that such a comparison would provide useful information to the

reader, and accordingly we have added a new supplementary figure to the revised manuscript ( Figure

S3 ) presenting an extensive analysis. Briefly, we detect at least one subunit for 70% of CORUM

complexes, and at least half of all subunits for 22%. Like all literature-curated protein interaction

databases, CORUM contains a certain fraction of spurious associations, and previously we have

found that only a subset of complexes in the CORUM database are amenable to detection by

co-fractionation-based approaches, for either biological or technical reasons (Stacey et al., *BMC*

*Genomics* 2018). Accordingly, these proportions rise considerably when limiting the analysis to this

subset of complexes, with 81% represented by at least one subunit and 31% by at least half of their

subunits, respectively. We observe a modest bias towards larger protein complexes, but broadly

similar rates of CORUM complex components across complexes of different sizes. Our networks

recall 7% of all co-complex interactions in the CORUM dataset, and 15% within the subset of

complexes amenable to detection by PCP; these numbers rise further to 40% and 48%, respectively,

when considering only CORUM interactions involving proteins that are actually represented in the

networks. We also include a new supplementary table ( Table S4 in the revised manuscript)

cataloguing the proportion of components from each CORUM complex detected in the network, as

well as the recall of each co-complex interaction in CORUM.

The Results section has been revised to incorporate this new analysis as follows:

At a precision of 70%, PrInCE recovered a total of 26,361 protein-protein interactions involving 1,049

proteins (Figure 3A and Table S3). Visualization of the adjacency matrix revealed a sparse network of

physical interactions, and confirmed recovery of well-known cellular protein complexes, such as the 26S

proteasome, the chaperonin containing TCP-1 complex, and RNA polymerase II (Figure 2E). To more

systematically assess the recovery of known protein complexes, we compared the network to the CORUM

database, finding 206 of 296 human protein complexes (69.6%) had at least one subunit represented in

the network (Figure S3A and C and Table S4). This proportion rose to 81.4% (96 of 118) when

considering only those complexes previously found to be amenable to detection by PCP [38] (Figure S3B

and D), and was broadly similar for protein complexes of different sizes (Figure S3E-F), albeit with a

modest bias towards large complexes. Among the 16,927 unique interactions detected in either condition,

4,046 represented co-complex interactions within known CORUM protein complexes, corresponding to a

recall of 6.9% of unique interactions in CORUM, and 15.0% within the subset of complexes amenable to

detection by PCP (Figure S3G).

**Figure S3. Recovery of known protein complexes in the IFN interactome.**

(A-B) Proportion of subunits from individual CORUM protein complexes represented in the IFN

interactome, for all of CORUM (A), with select complexes highlighted, or the subset previously found to be

amenable to detection by PCP [38], (B).

(C-D) Proportion of CORUM protein complex subunits detected for all CORUM complexes, (C), or the

subset amenable to detection by PCP, (D).

(E-F) As in (C-D), but stratified by protein complex size (number of subunits).

(G) Recall of co-complex interactions between CORUM complex subunits, between all known subunits or

the subset of subunits detected in the network, for either all of CORUM, top, or the subset amenable to

detection by PCP, bottom.

Pg 7 Line 34. Can you be more specific wrt how many interactions were novel and clarify in the main

text how novelty was assessed?

The sentence in question has been revised to specify the number and percentage of novel

interactions, and the assessment of novelty. Additionally, we have added InnateDB to the list of

protein-protein interaction databases used to assess novelty.

Despite this enrichment, however, comparison of the IFN interactome to literature-curated protein-protein

interactions recorded in eighteen databases revealed that the majority of interactions (13,240 of 16,927, or

78.2%) detected were novel.

Pg 8 the correlations between autocorrelation and evolutionary rates such as dn/ds are far from

convincing. I was not convinced that this analysis added much to the paper.

The magnitudes of these correlations are, as the reviewer correctly points out, relatively modest. We

have revised the text to reflect the strength of the associations. We have conducted a number of

additional analyses that consistently support the presence of modest, but statistically significant,

associations under a number of different analytical frameworks, suggesting they are indeed robust.

First, we compared the Spearman correlation (a nonparametric, rank-based method) and ordinary

least squares linear regression between the autocorrelation z score and the three different

evolutionary outcomes. We consistently observed statistically significant associations using either

method. This suggests that the associations are not driven by a small number of outliers. We include

a new panel in this figure in the revised manuscript, Figure S6E, showing this analysis (included

below).

Second, we also considered the possibility that our results might be driven by outliers at the SEC

chromatogram level, which could result in artifactually large autocorrelation z scores for a subset of

proteins. To address this possibility, we repeatedly sampled with replacement from the raw

chromatograms, then re-did the autocorrelation analysis, effectively allowing us to sample

autocorrelation values from the empirical distribution of the observed PCP data. If the rank correlation

observed over the entire dataset was an artefact of a relatively small number of unreliable data points

in the raw chromatograms, then we might expect that the Spearman correlation in bootstrapped PCP

datasets would frequently change sign (in other words, we would sometimes observe a positive

correlation instead of a negative one). However, this was not the case. We include the distribution of

Spearman correlation values for dN/dS, phylogenetic breadth, and pLI in the revised Figure S6B-D:

**Figure S 6 . Evolutionary plasticity of the interactome response to IFN stimulation.**

(A) Distribution of autocorrelation z scores between IFN-stimulated and unstimulated protein correlation

profiles, derived from Stouffer integration across all three replicates. Selected highly rewired proteins are

highlighted.

(B) Top, relationship between autocorrelation z score and ratio of nonsynonymous to synonymous

substitutions (dN/dS; (Spearman ρ = –0.11, p = 1.9 × 10 –4 ). Bottom, distribution of Spearman correlation

coefficients when sampling with replacement from SEC-PCP-SILAC chromatograms and re-calculating

the autocorrelation z score between IFN-stimulated and unstimulated protein correlation profiles.

(C) Top, relationship between autocorrelation z score and proportion of genomes in the InParanoid

database [50] in which an ortholog of the protein was present ρ = 0.14, p = 5.3 × 10 –7 ). Bottom, distribution

of Spearman correlation coefficients when sampling with replacement from SEC-PCP-SILAC

chromatograms and re-calculating the autocorrelation z score between IFN-stimulated and unstimulated

protein correlation profiles.

(D) Top, relationship between autocorrelation z score and pLI score quantile (Spearman ρ = 0.18, p = 5.9

× 10 –5 ). Bottom, distribution of Spearman correlation coefficients when sampling with replacement from

SEC-PCP-SILAC chromatograms and re-calculating the autocorrelation z score between IFN-stimulated

and unstimulated protein correlation profiles.

(E) Spearman correlation coefficients, left, and linear regression coefficients, right, between the

autocorrelation z score and ratio of nonsynonymous to synonymous substitutions (dN/dS), the proportion

of genomes in the InParanoid database in which an ortholog of the protein was present (phylogenetic

breadth), and the pLI score quantile (pLI).

Third, we adopted an analytical paradigm in which proteins were grouped into four equally sized bins

based on their autocorrelation between stimulated and unstimulated protein correlation profiles.

Conceptually, this is equivalent to consolidating the continuous spectrum of autocorrelations into four

discrete groups of proteins, that is: “most rewired,” “more rewired, “less rewired”, and “least rewired.”

We then plotted the mean and standard error of each outcome within each bin, and tested the

statistical significance of the association using the Kendall rank correlation, again finding all three to

be statistically significant (p < 0.001).

**Response figure 1.2.** Relationship between protein autocorrelation quartiles, from most to least rewired

in response to IFN stimulation, and dN/dS (left), phylogenetic breadth (middle), and pLI score quantile

(right).

We have opted not to include the analysis in the manuscript, owing to the well-known issues

associated with binning continuous data (e.g., Altman and Royston, *Br. Med. J.* 2006; Austin and

Brunner, *Stat. Med.* 2004). We feel the methods presented in Figure S6 are a more methodologically

appropriate way of depicting and testing for the associations of interest. However, we include this

analysis in the response as a further demonstration of the robustness of the associations between

interaction rewiring (as quantified by the autocorrelation) and evolutionary outcomes to various

different analytical strategies.

Collectively, these results reflect that, although the magnitudes of the observed associations are

modest, they are consistently statistically significant and robust to exactly how the analysis is

performed. We have revised the relevant paragraph of the manuscript to emphasize the modest

magnitude of the associations and refer to the additional analyses presented in the supplementary

figure as follows:

Comparing the IFN-induced autocorrelation of each protein to its evolutionary rate, as quantified by the

ratio of nonsynonymous to synonymous substitutions (dN/dS), revealed a modest, but statistically

significant negative correlation (Spearman’s ρ = –0.11, p = 1.9 × 10 –4 ; Figure S 6 B). Similar ly modest but

significant associations conclusions were observed in comparisons to the total number of species in which

an ortholog of a given gene was present (ρ = 0.14, p = 5.3 × 10 –7 ; Figure S 6 C) [44] , or to the pLI score

[45] , a measure of mutational constraint derived from large-scale human exome sequencing (ρ = 0.18, p =

5.9 × 10 –5 ; Figure S 6 D). Moreover, these associations remained consistent when controlling for changes

in protein abundance observed in the shotgun proteomics data using the partial Spearman correlation (p ≤

2.6 × 10 –4 ), when analyzing the data using linear regression (p ≤ 6.2 10 –3 ; Figure S6E), and when

sampling with replacement from the underlying SEC-PCP-SILAC chromatograms (Figure S6B-D).

Collectively, these results indicate that rapidly evolving proteins are disproportionately rewired in the

protein-protein interaction network by IFN signalling, to a degree that cannot be explained by changes in

protein abundance alone, suggesting that species-specific differences in the innate immune response may

be mediated in part through the effects of protein sequence divergence on protein-protein interactions.

Pg 10 Given that STAT1 does not pull down IFI35, isn't it just as possible that the coil-coiled domain

of IFI35 is required for a direct interaction with another protein (possibly NMI) and the influence on

STAT1 activity is indirectly mediated by an interaction with one or more proteins other than STAT1?

We believe the reviewer may be mixing up the pulldowns conducted. When pulling on STAT1 we

observe IFI35 co-precipitating, but not NMI (Figure 5E; left panel). Doing the reciprocal pulldown (i.e.

pulling down IFI35), we do not see an appreciable enrichment of STAT1, but do see NMI (Figure 5E,

now S8C, middle panel). This led us to hypothesize that while some of STAT1 is engaged in an

interaction with IFI35, not all IFI35 is engaged with STAT1. We apologize if this was not clear in the

main text and have adjusted the figures to better highlight which protein was the bait. Moreover, we

have added additional data demonstrating that we can recover the STAT1-IFI35 interaction in THP-1

cells (Figure S8D) . Regarding the reviewer’s comments on whether the interaction is direct via the

coiled-coil domain of IFI35, we agree that it is possible that another component may be participating in

this complex, however our data suggests that it is not NMI since we did not recover NMI in any of our

data as interacting with STAT1. Furthermore, we provide new data that suggests the coiled-coil region

of IFI35 is in actuality required for correct localization of this protein (Figure 5G):

**Figure 5.** (G) Immunofluorescence micrographs of cells mock transfected or transfected with HA-tagged

IFI35 expression constructs. Shown is a representative image. Scale bar = 10 μm.

We discuss these observations, and have slightly adjusted our conclusions about how IFI35 may be

downregulating STAT1 activity, in the revised Results section as follows:

To determine if truncating the coiled-coil region perturbs IFI35 function, we examined the localization of

wildtype HA-IFI35 or its truncated form upon IFN stimulation. Immunofluorescent staining revealed that

wildtype HA-IFI35 formed punctate cytoplasmic granules with IFN stimulation, as previously observed

(Figure 5G) [61, 62]. Strikingly, truncation of the first 30 N-terminal amino acid residues of IFI35

dramatically shifted the localization, to diffuse cytoplasmic and nuclear staining (Figure 5G). This data

suggests that disruption of the IFI35 N-terminal region may alleviate inhibition of STAT1 activity by

redistributing IFI35 localization in the cytoplasm.

Taken together, these data support a model whereby IFI35 interacts with STAT1, sequestering it to

cytoplasmic granules to fine-tune STAT-1-mediated transcription in response to IFN stimulation.

Table S1 the 4th sheet in the file is labelled "Statistical Analysis - 8h +IFN" - I assume this should be

"Statistical Analysis - 24h +IFN". Hopefully this is just a labelling error?

Yes, the reviewer is correct that this was a labeling error. We apologize and have fixed it.

Table S1. In the 3rd sheet many of the adjusted P-values have exactly the same value (to 9 decimal

places). This is probably because a rounded value for the unadjusted P-values were used in the

correction algorithm. This is unlikely to change anything - it just looks unusual.

The Benjamini-Hochberg correction procedure used to obtain the adjusted P-values reports the lowest

false discovery rate threshold below which the test can be considered significant. In the case of the

third sheet of Table S1, the expected proportion of false discoveries among the rejected null

hypotheses is never lower than 1.22%, and consequently the first 35 p-values all yield an adjusted

p-value (or q-value) of 0.0122, identical to nine decimal places as the reviewer notes. An analogous

situation occurs for many possible false discovery rates, meaning there are fewer distinct q-values

than p-values throughout the dataset (see Response figure 1.3 ):

**Response figure 1.3.** Adjusted p-values (q-values) corresponding to the first 500 p-values in the shotgun

proteomics comparison of cells stimulated with IFN-β for 4 h and unstimulated cells.

**Reviewer #2**

In the paper "Dynamic rewiring of the human interactomes by interferon signalling" authors employ

size exclusion chromatography coupled with MS to study the changes in protein-protein interactions

24 hours after type I Interferon stimulation. By applying PrInCE method, the authors predict 27694

binary interactions at 70% precision.

The paper is nicely written and the figures are of high quality. The rich interaction dataset, such as the

one presented here, would be of high relevance for understanding the molecular mechanisms of

immunity given the interactions are true and reliable. Many observations derived from the data are

quite speculative and not sufficiently substantiated by proper controls or validation experiments at this

point. However, the manuscript definitely holds a lot of promise and could be very interesting to the

broader field of biologists.

We thank the reviewer for their positive comments on our manuscript.

Major points:

1. The measurement of IFN-induced changes to SEC-profiles takes 24h after the treatment. Even

though I do understand that 4h (very earls) IFN treatment does not elicit major protein abundance

changes 24h time point is a quite late time point. It is expected that, in addition to immediate changes

to IFN-signalling, protein abundance and interactome changes will propagate to multiple downstream

pathways. This notion is supported by the broad range of GO terms enriched at 24h post treatment.

While also interesting, these changes are not necessarily specific to immune response. In particular,

at this time point it's expected to observe downregulation of a some proteins, disassembly of

corresponding protein complexes and negative regulatory processes - thus the data becomes very

difficult to interpret given that multiple processes are operating in parallel. The authors should clearly

comment on that in the text.

We agree with the reviewer that the observed changes in protein abundance and interactome

connectivity likely reflect a combination of direct and indirect effects. We include the following new

paragraph in the Discussion, commenting on the time point selected for these experiments and

potential implications:

After 24 h of IFN stimulation, we observed dynamic rearrangements of the cellular protein-protein

interaction network, affecting a broad spectrum of cellular pathways. At this relatively late timepoint, the

observed interactome rewiring likely reflects not only the direct effects of IFN signalling, but also the

downstream effects of IFN stimulation on cellular processes such as chromatin remodelling and

metabolism, as observed in our shotgun proteomics dataset (Figure 1). These changes in protein

abundance and network connectivity may reflect the establishment of an antiviral state in uninfected

‘bystander’ cells exposed to circulating IFN, similar to that observed during viral infections [71–73].

2. The authors use the term "interaction rewiring" or "remodeling" without clearly defining it. Given the

significant changes to the protein abundance, it's expected that the abundance of the related protein

complexes would be also changing. E. g. IFN-induced changes to immunoproteasome SEC-profile

shown on Figure 2B (what proteins were used?) could be explained solely by the regulation of the

proteasome subunits abundance, whereas the interaction affinities (which the term "rewiring" implies)

may stay unchanged (the same peaks observed in stimulated and unstimulated conditions). Did the

authors regarded such cases as IFN-specific interactions or required that the SEC peak positions and

shapes were significantly changed? In some extreme cases (e.g. protein is exclusively expressed

upon interferon stimulation) SEC profile may only be available in one condition. While both affinityand

abundance-caused changes are interesting and biologically relevant, they imply different

mechanisms and affect downstream analysis (e.g. Figures 3H, 3I, S3, Figure 4), therefore should

clearly be deciphered by the type of change and be annotated in Table S3.

Both reviewers expressed a desire to better understand the factors that likely drive the observed

interactome rewiring in response to IFN stimulation. To address the questions raised by the reviewer,

we have added a new row of panels to Figure 3, and a new supplementary figure (Figure S4) to the

revised manuscript. Briefly, we find that between 21–35% of interactions, but only 7–16% of proteins,

are specific to one of the two networks. Although >6,500 interactions involve a differentially expressed

protein, the vast majority of these proteins undergo only subtle (that is, less than twofold) changes in

abundance by 24 h. A much smaller subset of interactions ( *n* = 652) involve at least one protein with a

larger (that is, twofold or greater) difference in abundance in IFN-stimulated cells; these interactions

are significantly overrepresented among the condition-specific interactions detected in our study. We

interpret these results to suggest that while subtle changes in protein abundance may act to ‘fine-tune’

interactions, e.g. through competition for a binding interface, abundance alone cannot fully account for

the observed interactome rewiring, suggesting a role for other cellular mechanisms.

The Results section has been revised to detail these new analyses as follows:

Among the 26,361 protein-protein interactions detected overall, 11,924 were identified in

unstimulated cells and 14,437 in IFN-stimulated cells, with 9,434 detected under both conditions (Figure

3B and Figure S4A-B). Thus, 71.6% of all detected interactions were identified in both networks. This

rewiring could not be attributed solely to the variable protein composition of the two networks, as a smaller

proportion of protein nodes themselves were specific to one of the two networks (Figure 3C). To

investigate the degree to which changes in protein abundance in response to IFN stimulation could

underlie the observed rewiring, we overlaid our shotgun proteomics data (at the 24 h timepoint; Figure 1C)

onto the IFN-stimulated and unstimulated networks (Figure S4C-D and Table S3). A total of 6,562 unique

interactions involved at least one protein with a statistically significant difference in abundance upon IFN

stimulation, but most of these proteins exhibited relatively subtle changes in expression (Figure 3D). In

contrast, fewer interactions involved proteins with more dramatic IFN-induced changes in abundance (i.e.

two-fold or greater). Moreover, whereas interactions involving differentially expressed proteins were

equally likely to be observed in either or both of the IFN-stimulated or unstimulated networks (p = 0.20, χ2

test), interactions involving proteins with twofold or greater differences in abundance were significantly

enriched among the set of condition-specific interactions (p < 10–15, χ2 test; Figure S4E). To further

assess this trend, we calculated, for all interactions, the maximum log2-fold change within the interacting

protein pair, and confirmed that only a small fraction of interactions involve proteins with dramatic changes

in abundance (Figure 3E and Figure S4F). Overall, these results suggest that only a small number of

condition-specific interactions involve proteins with pronounced IFN-induced changes in abundance. More

subtle changes in expression affect a larger number of interactions, and might act to ‘fine-tune’

interactions. However, the large number of interactions between proteins that are not differentially

expressed in response to IFN stimulation suggests a role for other mechanisms, such as post-translational

modification, subcellular localization, or protein degradation, in IFN-dependent interactome remodeling.

**Figure 3. Biological relevance of the IFN interactome** *[...]*

(B) Proportion of all detected interactions observed both networks or in one condition only.

(C) Number of interactions in the unstimulated and IFN-stimulated network detected in both networks or in

one condition only.

(D) Number of proteins for which at least one interaction was detected in the unstimulated and

IFN-stimulated network or in one condition only.

(E) Number of interactions in the unstimulated and IFN-stimulated network involving a protein for which a

statistically significant change in abundance was detected at 24 h by SILAC shotgun proteomics at 5%

FDR, and the subset of these for which a twofold or greater change in abundance was detected.

(F) Maximum log 2 -fold change among interacting protein pairs detected in both networks, the unstimulated

network only, or the IFN-stimulated network only.

**Figure S4. Visualization of the unstimulated and IFN-stimulated interactomes.**

(A-B) Network diagrams of the protein-protein interaction networks detected by SEC-PCP-SILAC in

unstimulated (A) and IFN-stimulated (B) cells.

(C-D) As in (A-B), but with proteins colored by their log 2 -fold change at 24 h, as detected by SILAC

shotgun proteomics.

(E) Top, number of interactions in the unstimulated and IFN-stimulated network involving a protein for

which a statistically significant change in abundance was detected at 24 h by SILAC shotgun proteomics

at 5% FDR, and the subset of these for which a twofold or greater change in abundance was detected, as

in Figure 3E. Bottom, as in top, but plotting the proportion of interactions in each category instead of the

absolute number.

(F) Distribution of maximum log 2 -fold change values among interacting protein pairs detected in both

networks, unstimulated cells only, or IFN-stimulated cells only

Finally, as requested, we have annotated each interaction in Table S3 with the categories presented

in Figure 3D): that is, (i) involving at least one differentially expressed protein; (ii) involving at least

one differentially expressed protein with a twofold or greater change in abundance; and (iii) neither

protein differentially expressed. In addition, we provide the log 2 -fold change and adjusted p-value for

each protein in an interacting pair.

3. Surprisingly, the central part of the study, the SEC-PCP-MS dataset itself and the derived

interactome, are not sufficiently represented in the figures and supplementary data. It would be very

beneficial to provide some visualization of the network, so that its global topology, as well as the

topology of the proposed IFN-specific subnetwork, could be observed by the readers (Figure 1E

doesn't allow that). Similarly, the network data provided in Table S3 is too scarce, since it only

provides the final precision scores. To facilitate the use of their dataset in follow-up bioinformatic

studies, the authors should at least provide the table with all the features used by their naive Bayes

classifier for each protein group, as well as the autocorrelation z score, which seem to correlate with

interaction rewiring upon IFN stimulation.

Both reviewers expressed a desire to visually compare the unstimulated and IFN-stimulated networks.

We include a new supplementary figure, Figure S4, with diagrams of both the stimulated and

unstimulated networks (see above). Furthermore, as requested, we have overlaid the protein

expression data onto these plots, in the form of log 2 -fold changes after IFN stimulation. Unfortunately,

these diagrams reflect the well-known limitation that complex networks with thousands of nodes and

edges are difficult to interpret visually (and consequently are often referred to as “hairballs”, e.g., Flint

and Ideker, *PLoS Genet* 2019). To provide a superior resource that will allow users to explore the

IFN-induced interactome network, we have developed an feature-rich web application, available at

http://ifn-interactome.msl.ubc.ca/, that allows users to interactively browse these network diagrams

themselves, as well as to explore the underlying PCP chromatograms. The network view further

allows users to optionally focus either on specific genes of interest, or the sets of genes that are

differentially expressed in meta-analyses of >100 different diseases, including the IFN-related

diseases depicted in Figure 3I. We have added a brief paragraph to the revised Discussion section

introducing this web application:

To facilitate exploration of the complete dataset, we have developed an interactive web application,

available at https://ifn-interactome.msl.ubc.ca, that allows users to visualize both the IFN interactome

network as well as the underlying PCP chromatograms. The network view further allows users to

optionally restrict the network either to specific genes that are of interest, or to entire sets of genes called

as differentially expressed in transcriptomic meta-analyses of 103 different diseases, including the

IFN-related diseases depicted in Figure 3I [41, 42].

Finally, to address the reviewer’s request for additional information on the features used by PrInCE to

predict condition-specific networks and IFN-induced interaction rewiring, we provide (i) a revised

version of Table S3 including all features used by the naive Bayes classifier for each pairwise

interaction, and (ii) a separate table with autocorrelation z scores (Table S5 in the revised

manuscript), which are calculated for individual proteins rather than protein pairs.

4. When assessing the quality of their IFN interactome (Page 6), the authors only discuss the

precision component. They should also provide the percentage of known interactions they recall at

70% precision level. This is critical information since it gives the reader confidence in the data. It

would be particularly interesting to check precision for their IFN-stimulated network. Adding InnateDB

(https://www.innatedb.com) to the broad list of public interaction databases they are already

considering should make this comparison more relevant - particularly since this database contains

interaction data in stimulated or infected conditions. Naturally, when calculating the precision of binary

interactions, the estimates are biased by big complexes (since the number of interactions grows as

n2, where n is the number of proteins in a complex). To overcome this, the authors should weight the

interactions by the sizes of molecular complexes (e.g. using 1/M as the weight of each binary

interaction, where M is the molecular weight of the putative protein complex deduced from the

corresponding peak of the SEC-PCP profile) or providing separate statistics for complexes of different

sizes.

Both reviewers requested a more thorough investigation of the degree to which our networks recall

known co-complex interactions. We agree that such a comparison would provide useful information to

the reader, and accordingly we have added a new supplementary figure to the revised manuscript

( Figure S3 ) presenting an extensive analysis. Briefly, the combined network recovers 7% of all

co-complex interactions in the CORUM dataset. Like all literature-curated protein interaction

databases, CORUM contains a certain fraction of spurious associations, and previously we have

found that only a subset of complexes in the CORUM database are amenable to detection by

co-fractionation-based approaches, for either biological or technical reasons. We found that when only

these complexes is considered, the recall rises to 15%. Moreover, the recall rises further to 40% and

48%, respectively, when considering only CORUM co-complex interactions involving proteins that are

actually represented in the networks. We include a new supplementary table (Table S4 in the revised

manuscript) cataloguing the recall of each co-complex interaction in CORUM, and the Results section

has been revised to incorporate this new analysis as follows:

At a precision of 70%, PrInCE recovered a total of 26,361 protein-protein interactions involving 1,049

proteins (Figure 3A and Table S3). Visualization of the adjacency matrix revealed a sparse network of

physical interactions, and confirmed recovery of well-known cellular protein complexes, such as the 26S

proteasome, the chaperonin containing TCP-1 complex, and RNA polymerase II (Figure 2E). To more

systematically assess the recovery of known protein complexes, we compared the network to the CORUM

database, finding 206 of 296 human protein complexes (69.6%) had at least one subunit represented in

the network (Figure S3A and C and Table S4). This proportion rose to 81.4% (96 of 118) when

considering only those complexes previously found to be amenable to detection by PCP [38] (Figure S3B

and D), and was broadly similar for protein complexes of different sizes (Figure S3E-F), albeit with a

modest bias towards large complexes. Among the 16,927 unique interactions detected in either condition,

4,046 represented co-complex interactions within known CORUM protein complexes, corresponding to a

recall of 6.9% of unique interactions in CORUM, and 15.0% within the subset of complexes amenable to

detection by PCP (Figure S3G).

**Figure S3. Recovery of known protein complexes in the IFN interactome.**

(A-B) Proportion of subunits from individual CORUM protein complexes represented in the IFN

interactome, for all of CORUM (A), with select complexes highlighted, or the subset previously found to be

amenable to detection by PCP [38], (B).

(C-D) Proportion of CORUM protein complex subunits detected for all CORUM complexes, (C), or the

subset amenable to detection by PCP, (D).

(E-F) As in (C-D), but stratified by protein complex size (number of subunits).

(G) Recall of co-complex interactions between CORUM complex subunits, between all known subunits or

the subset of subunits detected in the network, for either all of CORUM, top, or the subset amenable to

detection by PCP, bottom.

The reviewer also requested that we add InnateDB to the list of interaction databases used to

evaluate novelty. We have revised the paragraph accordingly to both clarify how novelty was

assessed, and specify both the number and proportion of interactions overlapping with eighteen

literature-curated databases, now including InnateDB.

Despite this enrichment, however, comparison of the IFN interactome to literature-curated protein-protein

interactions recorded in eighteen databases revealed that the majority of interactions (13,240 of 16,927, or

78.2%) detected were novel.

Finally, the reviewer suggests that we should weigh the contributions of individual interactions in the

calculation of precision based on the size of known complexes. Precision is a widely used metric that

has a single accepted meaning, the ratio of the number of true positives to the number of true

positives and false positives, and our approach here is consistent with how similar co-fractionation

datasets are typically analyzed within this field (e.g., Havugimana et al., *Cell* 2012; Wan et al., *Nature*

2015; Moutaoufik et al., *iScience* 2019). The reviewer did suggest that as an alternative, we should

provide statistics for complexes of different sizes. Consequently we have incorporated complex size

into several panels of Figure S3 of the revised manuscript, most directly in Figure S3E-F (see above),

where we directly evaluate the proportional representation of protein complex subunits in the networks

as a function of their size.

5. When commenting on the relevance of the IFN interactome, the authors show that it's represented

among the genes implicated in IFN-related diseases (Page 7, Figure 3I). While this observation

suggests that the interactome is not random, it doesn't necessary prove that it extends our

understanding of molecular mechanisms over the existing knowledge. To show that, the authors

would need to compare the enrichment of novel interactions over the already published ones, and/or

show that this enrichment is specific to immunity-related diseases over the unrelated ones. Likewise,

showing the estimates for the already known global interactome on Figures 3B-3F, as well as adding

its coexpression and phylogenetic similarity profiles to Figure 3G, should provide an important

reference for assessing the IFN interactome results.

In our original manuscript, we observed that many protein-protein interactions involving genes that are

differentially expressed in IFN-related diseases were detected only after IFN stimulation. The reviewer

points out that this fact alone does not necessarily extend our mechanistic understanding of the roles

of these genes in the IFN response. This would be the case if either (i) interactions involving DE

genes are equally likely to be observed in the unstimulated cells, or (ii) interactions involving DE

genes are equally likely to be observed in literature-curated databases of published protein-protein

interactions.

To address this comment, we have performed the requested analyses. We first asked whether genes

that are upregulated in IFN-related diseases are more likely to be found in IFN-stimulated interactions

only. For ten of eleven diseases, we indeed found that interactions involving these DE genes are

significantly more likely to be IFN-specific. We next asked whether IFN-related disease genes are

more likely to be found in IFN-stimulated interactions than in literature-curated databases, that is, the

“already known global interactome,” and found for all eleven diseases that these genes were

significantly more likely to be found in IFN-specific interactions. We include these findings in Figure

S5H of the revised manuscript (included below).

We have revised the relevant paragraph of the Results section as follows:

We mapped protein-protein interactions for dozens to hundreds of differentially expressed genes from

each disease (Figure 3 M ); notably, interactions for many such gene products were identified exclusively in

the IFN-stimulated condition. For ten of eleven diseases, genes upregulated at a 1% FDR were

significantly enriched among interactions detected only after IFN stimulation (Figure S5H). Moreover, for

all eleven diseases, these genes were significantly enriched among IFN-specific interactions compared to

literature-curated human interactions compiled from eighteen databases (Figure S5H). Thus, the IFN

interactome provides a reference to understand the consequences of dysregulated IFN signalling in a

diverse range of human pathologies, by placing transcriptional markers of auto-inflammatory disease into

a functional context.

Separately, the reviewer requested we compare the validation experiments presented in Figure 3B-G

to known interactions. We show the same analyses performed on the 9,511 human physical

interactions catalogued in the InnateDB database, as well as a head-to-head comparison between the

IFN interactome and InnateDB, in Figure S5A-G of the revised manuscript. Overall, we found that by

most measures, low-throughput interactions catalogued in InnateDB show a slightly stronger

signature of functional enrichment, although the difference is relatively modest in most cases (see

below):

**Figure S5. Comparison of the IFN interactome to literature-curated interactions associated with the**

**innate immune response.**

(A-C) Proportion of interacting protein pairs sharing at least one biological process (A), cellular

compartment (B), or molecular function (C) Gene Ontology term in the InnateDB database, arrow, or

1,000 randomly rewired networks, histogram.

(D) Proportion of interacting protein pairs implicated in the same disease in the InnateDB database, arrow,

or 1,000 randomly rewired networks, histogram.

(E) Proportion of interacting protein pairs supported by a domain-domain interaction in the InnateDB

database, arrow, or 1,000 randomly rewired networks, histogram.

(F) Pearson correlations reflecting protein abundance and phylogenetic profile similarity between

interacting protein pairs in the IFN interactome or the InnateDB database.

(G) Enrichment for shared Gene Ontology terms, disease gene annotations, or domain-domain

interactions in the IFN interactome and the InnateDB database, shown as z scores relative to 1,000

randomly rewired networks.

(H) Odds ratio of IFN-specific interactions involving a gene product upregulated in eleven diseases

characterized by an elevated IFN transcriptional signature, relative to all other interactions detected by

SEC-PCP-SILAC, left, or literature-curated interactions recorded in eighteen databases, right.

The following sentence has been added to the relevant paragraph of the Results section to note these

analyses:

The enrichment for known correlates of physical interaction observed in the SEC-PCP-SILAC network

was broadly comparable to, although slightly lower than, literature-curated interactions from small-scale

experiments compiled in the InnateDB database (Figure S5A-G) [40].

6. The authors apply regression analysis to show that SEC-profile changes upon type I IFN

stimulation seem to positively correlate with the rate of protein evolution (Page 8, Figures S3B-S3D).

It's not clear from the methods whether the SEC-profile autocorrelation z-score was corrected for

protein abundance changes to exclude that the observed effect is merely due to the upregulation of

interferon-stimulated genes (ISGs). One way to address this is to show that the observations still hold

when the regression analysis is limited to ISGs only. The authors highlight IFIT proteins as one of the

top rewired proteins. Indeed, it's published that IFITs form the heterodimer complex upon IFN

stimulation, so it is very interesting whether, on top of the dramatic upregulation, the authors could

see the corresponding peak shift in their SEC-MS profiles, or whether it's so low-abundant in

non-stimulated conditions that they don't detect any peaks at all.

To address the reviewer’s first comment, that the observed correlations between the rate of protein

evolution and the SEC autocorrelation z score can be explained by the upregulation of ISGs, we

explicitly controlled for changes in abundance observed in the shotgun proteomics data using the

partial Spearman correlation. Specifically, we used the R package ‘ppcor’ to test the partial Spearman

correlation between the SEC autocorrelation (or, equivalently, its z score) and the three evolutionary

outcomes of interest, while controlling for the log 2 -fold change at 24 h in the shotgun proteomics

dataset. We found that all correlation coefficients remained statistically significant, and had very

similar absolute values. Thus, changes in protein abundance do not appear to account for the

observed relationships between IFN-dependent interaction rewiring and protein evolution.

| **Outcome** | **Correlation** | **P-value** |
| --- | --- | --- |
| dN/dS | Spearman correlation: –0.11  Partial Spearman correlation: –0.11 | Spearman correlation: 2.0 × 10 –4  Partial Spearman correlation: 2.6 × 10 –4 |
| Phylogenetic breadth | Spearman correlation: 0.14  Partial Spearman correlation: 0.13 | Spearman correlation: 5.3 × 10 –7  Partial Spearman correlation: 1.2 × 10 –5 |
| pLI | Spearman correlation: 0.18  Partial Spearman correlation: 0.18 | Spearman correlation: 5.9 × 10 –5  Partial Spearman correlation: 5.6 × 10 –5 |

We have revised the paragraph in question as follows:

Comparing the IFN-induced autocorrelation of each protein to its evolutionary rate, as quantified by the

ratio of nonsynonymous to synonymous substitutions (dN/dS), revealed a significant negative correlation

(Spearman’s ρ = –0.11, p = 1.9 × 10 –4 ; Figure S 6 B). Similar conclusions were reached by comparisons to

the total number of species in which an ortholog of a given gene was present (ρ = 0.14, p = 5.3 × 10 –7 ;

Figure S 6 C) [47] , or to the pLI score [48] , a measure of mutational constraint derived from large-scale

human exome sequencing (ρ = 0.18, p = 5.9 × 10 –5 ; Figure S 6 D). Moreover, these associations remained

consistent when controlling for changes in protein abundance observed in the shotgun proteomics data

using the partial Spearman correlation (p ≤ 2.6 × 10–4), when analyzing the data using linear regression

(p ≤ 6.2 10–3; Figure S6E), and when sampling with replacement from the underlying SEC-PCP-SILAC

chromatograms (Figure S6B-D). Collectively, these results indicate that rapidly evolving proteins are

disproportionately rewired in the protein-protein interaction network by IFN signalling, to a degree that

cannot be explained by changes in protein abundance alone, suggesting that species-specific differences

in the innate immune response may be mediated in part through the effects of protein sequence

divergence on protein-protein interactions.

The reviewer also asked whether we observe a peak shift in the SEC chromatograms of the IFIT

proteins. A suggestive shift is indeed observed, particularly for IFIT1 and IFIT3, which appear to form

two peaks: these might correspond to the previously reported heterodimeric and heterotetrameric

complexes the reviewer refers to, although missing values in the quantitation of IFIT2 limit confidence

in this interpretation. Nevertheless, it is also the case that the correlation between these two proteins

increases markedly after stimulation.

**Response figure 2.1.** Top, SEC-PCP-SILAC chromatograms for IFIT1, IFIT2, and IFIT3 in a

representative replicate. Bottom, pairwise correlations between IFIT1 and IFIT3 before and after IFN

stimulation in the same replicate.

More generally, because the autocorrelation quantifies the difference in interactome-wide protein

interaction profiles between two conditions based on the PCP chromatograms, all of the proteins

highlighted in this panel were detected at least at low abundance in the unstimulated condition. This

can likely be attributed at least in part to the sensitivity afforded by the use of a mixed reference,

including both stimulated and unstimulated samples, in the SILAC design.

7. The reviewer had difficulties assessing the relevance of the hypothesis that evolutionary conserved

ISGs ("core ISGs") are more engaging in physical interactions than the less conserved ones (page 9).

The authors didn't provide the list of the proteins which they considered for each of those classes,

neither they provided the underlying data for Figure 5C (validation of "core ISGs" hypothesis by

AP-MS experiments with STAT1, IFI35 and NMI baits). In any case, so far this hypothesis is

supported only by the data obtained from HeLa cells, which represent <25% of proteins (2590 protein

groups) of one particular (and severely altered) human tissue. It's known that the repertoire of

immunity-related proteins and their regulation vary dramatically across different cell types, so

"human-only" and "conserved" ISGs may be more engaged in interactions in the other cell types. The

authors also have to rule out the alternative explanations for the enrichment they observe (Figure 5A),

e.g. whether "core ISGs" coincide with the particular biological process (GO BP), molecular function

etc. Does the hypothesis still hold when, instead of profile correlations, the authors test for the number

of ISG-specific interactions they predict? If Figure 5C shows the ranks of enrichment in IFN+ vs IFNIPs

of IFI35, NMI and STAT1, is it corrected for protein abundance changes of corresponding genes

upon IFN stimulation? Does the figure only show the specific interactors of the corresponding baits?

First, we apologize that the data supporting this analysis did not accompany the original manuscript.

In the revised manuscript, we include a new supplementary table, Table S7, that includes both the

raw AP-MS data and its statistical analysis, including protein memberships in the three categories of

ISGs.

Second, the reviewer points out that this analysis is supported only from data in HeLa cells. This is

certainly true, and we have added a new paragraph to the Discussion to reflect this, included below.

We have also repeated our experimental validation of one such induced interaction between two core

ISGs, i.e. STAT1–IFI35, in a second, monocyte-derived cell line, THP-1 (Figure S8D). Our ability to

reproduce the IFN-dependent interaction between STAT1 and IFI35 in THP-1 cells does suggest that

at least a subset of these interactions may be induced by IFN stimulation across multiple different cell

types or tissues.

Third, the reviewer requested we consider alternative explanations for the observed enrichment by

asking whether the ‘core ISGs’ have other enriched GO terms. We performed the requested analysis,

finding the most enriched terms to be: “defense response to virus,” “negative regulation of viral

process,” “regulation of symbiosis, encompassing mutualism through parasitism,” “response to biotic

stimulus,” “type I interferon signaling pathway,” “regulation of viral life cycle,” and “response to type I

interferon.” This suggests the observed enrichment is unlikely to be explained by a fortuitous overlap

between the “core ISGs” and a different, irrelevant biological process, but indeed reflect genes known

to be activated by IFN signalling and viral infection.

Fourth, the reviewer asks whether the hypothesis holds when we analyze the inferred networks

directly, rather than the raw SEC-PCP-SILAC chromatograms. Indeed, when we use the same

network rewiring-based enrichment test described in Figures 4 and S4 to test for a relative excess of

interactions between each set of ISGs between the stimulated and unstimulated interactomes, we

obtain the same results, with a significant enrichment for interactions between core ISGs (p = 0.0072),

but not conserved (p = 0.45) or human-specific (p = 0.35) ISGs.

Finally, the reviewer asks whether Figure 5C shows specific interactors only. Our

immunoprecipitations pulled down hundreds of proteins, which are unlikely to all be bona fide

interactors. However, non-specific interactors should have changes randomly distributed around zero,

which motivated our analytical approach using gene-set enrichment analysis (GSEA). To empirically

test the effect of non-specific interactors, we removed baits found in between 5% and 95% of

pulldowns from the CRAPome resource (Mellacheruvu et al., *Nat. Methods* 2013). We found that the

observed enrichment for core ISGs remained robust and specific in all the same three of four

pulldowns as in the original analysis. This analysis is included in Figure S8B of the revised

manuscript:

**Figure S8B.** Gene set enrichment analysis p-values for core, conserved, and human-specific ISG

products in comparisons of immunoprecipitations of IFI35, NMI, PNPT1, and STAT1 from IFN-stimulated

or unstimulated cells, after removal of potential nonspecific interactors found in between 5% and 95% of

human pulldowns in the CRAPome database.

8. Essentially, the authors put forward two different evolutionary hypotheses: one that evolutionary

plasticity results in more rewiring, the other one is that rewiring is more observed for evolutionary

conserved ISGs. Perhaps, the authors should propose one united hypothesis, since combining these

two together seems nontrivial.

We don’t believe these two hypotheses are as conflicting as they might seem. To clarify:

1. Hypothesis #1 (data in Figure S6 of the revised manuscript) is that proteins that diverge more

rapidly at the amino acid sequence level also exhibit a greater degree of ‘interaction rewiring’

upon IFN stimulation: in other words, these proteins are particularly likely to form new

interactions, or lose existing ones, during the IFN response.

2. Hypothesis #2 (data in Figure 5) is that a subset of ISGs, whose upregulation in response to

IFN stimulation is conserved over evolutionary time, are induced to engage in physical

interactions with one another after IFN stimulation.

The important observation that connects these two hypotheses is that proteins involved in the innate

immune response are among the most rapidly-evolving in mammalian genomes (e.g., Lindblad-Toh et

al., *Nature* 2011; Yue et al., *Nature* 2014; Lilue et al., *Nat. Genet.* 2018). Thus, a conserved *function*

in the innate immune response does not preclude divergence at the protein sequence level.

Consequently, we expect that although the “core ISGs” have a highly conserved induction in response

to IFN that spans ten different species, their primary sequences will in fact be significantly more

divergent than the genomic average. Indeed, this is what we find: “core ISGs” have a significantly

higher dN/dS ratio than the genomic average (p = 2.0 × 10 –8 , Brunner–Munzel test). We show this in

Figure S8A of the revised manuscript:

**Figure S8A.** Ratio of nonsynonymous to synonymous substitutions (dN/dS) between human and mouse

for ‘core ISGs’, induced in response to IFN stimulation in ten vertebrate species, and all other human

genes.

We believe the apparent conflict between these two hypotheses stems from a lack of clarity in our

description of our hypothesis and the procedure used to test it. Accordingly, we have revised the

relevant paragraph of the manuscript as follows:

Large-scale transcriptomic studies of the innate immune response across species and cell types have

contrasted extensive heterogeneity in species- or cell type-specific transcriptional programs with a core

module of universally upregulated genes, anchoring the transcriptional response across evolutionary and

cellular contexts [7–9] . We sought to characterize the properties of evolutionarily conserved and

species-specific ISGs at the interactome level. Using data from a comparative transcriptomic study of ten

vertebrates [7] , we defined sets of genes upregulated by IFN in all species (“core ISGs”), in human and at

least one other species (“conserved ISGs”), or in humans only (“human-specific ISGs”). We then asked

whether our SEC-PCP-SILAC data supported the hypothesis that ISGs in each category are induced to

interact physically with one another in response to IFN stimulation. To address this hypothesis, we

calculated the correlation between all pairs of ISGs in each condition, and tested for a significant increase

in the median correlation. Strikingly, only the core set of evolutionarily conserved ISGs displayed a

significant shift in chromatogram correlation upon IFN stimulation (p = 6.9 × 10 –3 , Brunner–Munzel test;

Figure 5A), suggesting these ISGs enter physical interactions or protein complexes during the IFN

response. Similar results were obtained when using the approach described in Figure S7A to test for

network rewiring, with significantly more interactions between core ISGs in the IFN-stimulated network (p

= 0.0072), but not conserved or human-specific ISGs (p ≥ 0.36). As expected, despite their evolutionarily

conserved induction in response to IFN stimulation, these core ISGs are also rapidly evolving at the

protein sequence level, consistent with the data presented in Figure S3 (p = 2.0 × 10 –8 , Brunner–Munzel

test; Figure S8A).

To experimentally validate this observation , we performed co-immunoprecipitations of four core

ISGs in unstimulated or IFN-stimulated cells (Figure 5B). In three of four co-immunoprecipitations, core

ISGs were significantly and selectively enriched among interactors induced by IFN stimulation, relative to

conserved or human-specific ISGs (gene set enrichment analysis, p ≤ 5.1 × 10 –3 ; Figure 5C). Moreover,

this enrichment was robust to filtering potential nonspecific interactors from the CRAPome database

(Figure S8B) [55].

9. Negative regulation of STAT1 by IFI35 was shown by Shirai et al in astrocytes

(https://www.ncbi.nlm.nih.gov/pubmed/28109979/). IFI35 mediated degradation of RIG-I results in

reduced IFNb cytokine expression. The reporter assay shown here indicates that treatment with

recombinant IFNb affects gene expression under control of ISRE. This is surprising since it implies

functions downstream of IFNb signaling. If the authors claim the same mechanism of action

(degradation), they should show degradation of STATs through IFI35. The STAT1-dependent

transcription experiment (Figure 5G) misses controls. The authors should show that the removal of

1-30 residues of IFI35 doesn't change its localization, and perform co-IP experiments to validate the

interaction of the coiled-coil domain of IFI35 and coiled-coil domain of STAT1. The authors suggest

that the interaction of STAT1 and IFI35 may be substoichiometric, does that mean that their

SEC-PCP-MS can predict transient interactions? The peaks of IFI35, NMI and STAT1 in

IFN-stimulated condition seem to be quite abundant (Figure 5B).

The reviewer notes that IFI35 has previously been shown to negatively regulate the IFN response in

the context of viral infection and poly(I:C) treatment. However, this likely occurs through RIG-I

degradation (which directly senses poly(I:C) and viral RNAs). In our work, we directly treat cells with

IFN, thus bypassing the need for signalling through sensors such as RIG-I. Instead, signalling occurs

directly through the well-established JAK-STAT pathway, which phosphorylates STAT1 whereupon it

subsequently translocates to the nucleus and activates transcription of target genes by binding ISREs

(Platanias et al., *Nat. Rev. Immunol.* 2005). The reviewer is correct that our results from the reporter

assay suggest that the negative regulation imposed by IFI35 on STAT1 occurs at a step between IFN

receptor binding and ISRE activation. Based on our data, we do not believe that STAT1 activity is

reduced through a degradation mechanism (Figure S8E), like RIG-I. We briefly discuss this possibility

in the revised Results section:

Of note, unlike previous work on RIG-I, we did not observe a decrease in STAT1 protein levels upon

overexpression of IFI35, suggesting substantial degradation is likely not occuring (Figure S8E).

We thank the reviewer for suggesting to assess localization of the IFI35 constructs. Surprisingly, we

found that truncating IFI35 dramatically changed its localization in the cell from a punctate granule-like

localization to diffuse cytoplasmic (Figure 5G). Given these observations we have revised our

conclusions on how IFI35 may be downregulating STAT1 activity to discuss this new data as follows:

To determine if truncating the coiled-coil region perturbs IFI35 function, we examined the localization of

wildtype HA-IFI35 or its truncated form upon IFN stimulation. Immunofluorescent staining revealed that

wildtype HA-IFI35 formed punctate cytoplasmic granules with IFN stimulation, as previously observed

(Figure 5G) [61, 62]. Strikingly, truncation of the first 30 N-terminal amino acid residues of IFI35

dramatically shifted the localization, to diffuse cytoplasmic and nuclear staining (Figure 5G). This data

suggests that disruption of the IFI35 N-terminal region may alleviate inhibition of STAT1 activity by

redistributing IFI35 localization in the cytoplasm.

Taken together, these data support a model whereby IFI35 interacts with STAT1, sequestering it to

cytoplasmic granules to fine-tune STAT-1-mediated transcription in response to IFN stimulation.

The reviewer also commented on the ability of SEC-PCP-SILAC to detect transient interactions. In

this context, we do not mean to imply that sub-stoichiometry arises via a transient interaction, but

instead that the interaction of IFI35 with STAT1 may not occur in a 1:1 ratio. We have revised the

Results section to clarify this as follows:

These observations suggest that the interaction between STAT1 and IFI35 may be substoichiometric,

whereby not all cellular IFI35 is bound to STAT1, or may potentially occur in an IFNβ-specific manner .

10. The authors show that the knock-down of RPL28 leads to upregulation of ISGs. However, the

observed effects are minor and most of the observed changes are not statistically significant, raising

the question of biological relevance of the observed knock-down effect. Further experiments are

required to confirm the hypothesis of RPL28 being the selective negative regulator of ISGs translation,

as it's still possible that the observed abundance changes might be a by-product of another process,

or be downstream of e.g. STAT1 regulation. Perhaps, the authors should explore their proteome data

for the alternative explanations (e.g. GO term enrichment analysis) as the volcano plot suggests there

is a number of proteins that are up- and down-regulated upon siRPL28 treatment.

To address this comment, we first conducted an unbiased GO term enrichment analysis of the RPL28

knockdown proteomics data, as suggested by the reviewer. Remarkably, we found that only 2 of

5,453 GO terms were more significantly enriched than the set of ISGs. Furthermore, both of these GO

terms (“positive regulation of interferon-alpha production” and “defense response to virus”) likewise

pointed strongly to a role for the antiviral response. These results reinforce the notion that knockdown

of RPL28 specifically affects ISG translation, rather than the observed abundance changes being a

byproduct of another process. In the revised manuscript, we include an additional panel in Figure 6,

as well as a new supplementary table (Table S10) presenting these analyses.

**Figure 6G.** P-values from gene set enrichment analysis (GSEA) of 5,453 GO terms in a comparison of

siRPL28-treated and IFN-stimulated cells compared to untreated controls. Dotted line shows the statistical

significance of ISGs by GSEA (Fig. S9E). Points in red represent GO terms more significantly enriched

than ISGs.

In addition to this analysis, we performed a new set of proteomic experiments designed to (i)

reproduce our original finding that knockdown of RPL28 results in upregulation of ISG protein

synthesis, and (ii) show that this effect is specific to knockdown of RPL28, rather than a consequence

of ribosomal protein perturbation more generally. To achieve this, we used a label-free quantitation

approach in combination with a timsTOF mass spectrometer, allowing us to achieve a substantially

deeper coverage of the HeLa proteome. We profiled HeLa cells treated with siRNAs targeting RPL28,

two additional ribosomal proteins (RPS26 and RPS28), and control siRNAs, each in four replicates.

Using this orthogonal technical system, we reproduced the highly significant upregulation of ISGs in

response to RPL28 knockdown (gene set enrichment analysis p-value = 7.1 × 10 –4 ), and found no

significant effects on ISG abundance after knockdown of either RPS26 or RPS28 (p = 0.69 and 0.18,

respectively). These experiments confirm our original observations that knockdown of RPL28 results

in increased ISG abundance, and provide evidence that this phenomenon is specific to knockdown of

RPL28. We describe these findings in the revised Results section as follows:

We next asked whether this phenomenon is specifically associated with knockdown of RPL28, or whether

it occurs in response to perturbation of ribosomal proteins more generally. To this end, we compared the

proteome of IFN-stimulated cells after treatment with siRNAs targeting RPL28 *,* RPS26 *,* or RPS28 using

label-free quantitation (Table S11). Following RPL28 knockdown, we reproduced the results from our

SILAC dataset, observing a significant increase in ISG abundance upon RPL28 depletion (p = 7.1 × 10 –4 ;

Figure S10A). However, this effect was not observed after depletion of RPS26 or RPS28 (p = 0.69 and

0.18, respectively; Figure S10B-C), suggesting that the increase in ISG abundance is specific to

perturbation of RPL28.

**Figure S10. Increased abundance of interferon-stimulated genes is specific to RPL28 knockdown.**

Gene set enrichment analysis barcode plots showing ranks of ISGs in a comparison of cells treated with

IFN-stimulated cells and siRNAs targeting RPL28 (A), RPS26 (B), and RPS28 (C), compared to cells

treated with control siRNA.

Finally, the reviewer brings up the possibility that upregulation of genes upon RPL28 knockdown may

be a consequence of STAT1 regulation: that is, increases in the abundance or activity of STAT1

would feed forward into increased ISG mRNA. However, if this were the case, we would expect to see

an increase in the mRNA levels of ISGs. With the exception of NMI, we do not observe any such

increase at the mRNA level in our RT-qPCR measurements (Figure S9G). Thus, we believe that a

feed-forward loop by STAT1 is unlikely to be the mechanism driving the increase in ISG protein

abundance observed upon RPL28 knockdown.

Minor points:

1. Page 5. Line 34 (Figures 1F,1G): It's not clear why protein interaction network randomization was

used to test whether IFN-affected proteins are enriched with pairwise interactions: essentially it tests a

weaker hypothesis, i.e. whether human interactome and the set of IFN-affected genes are both

non-random. It looks like the appropriate test for the original hypothesis would be to keep the

interactome intact and randomize just the set of proteins. Even more relevant would be to check

whether the observed enrichment in interactions is specific to IFN stimulation in comparison to the

other stimuli. To check this the authors may consider MSigDB C2 CGP sets

(http://software.broadinstitute.org/gsea/msigdb/genesets.jsp?collection=CGP).

Both reviewers raised questions about the analysis presented in Figure 1F-G, and we apologize that

this was unclear in the original manuscript. We have conducted the suggested analysis, wherein the

number of protein-protein interactions between DE proteins in literature-curated databases is

compared to a null model consisting of random sets of proteins of equal size ( **null 1** ), as opposed to

the null model presented in the original manuscript, which consisted of literature-curated protein

interaction networks rewired using degree preserving randomization ( **null 2** ). Reassuringly, there is an

even more significant enrichment for protein-protein interactions between proteins that are DE in

either comparison.

**Response figure 1.1.** Number of protein-protein interactions in the InBioMap database [22] between

differentially expressed proteins after 24 h (left) of IFNβ stimulation, or between cells stimulated for 4 and

24 h (right), arrows, and between proteins in 100 random sets of proteins of equivalent size, histograms.

However, we have elected not to include this analysis in the revised manuscript. Instead, we have

rewritten the paragraph in question extensively to clarify the motivation underlying the choice of **null**

**2** .

In contemplating this motivation, it is helpful to explicitly state our null hypothesis, which is as follows:

proteins that are differentially expressed in response to IFN stimulation do not interact with one

another any more frequently than we would expect *for a random set of proteins with equivalent*

*topological properties in the known human interactome network* . The importance of controlling for

network topology arises from the fact that in known protein-protein interaction networks, some

proteins participate in hundreds or even thousands of interactions, whereas others participate in only

a handful. Consequently, an arbitrary set of proteins may have an extremely high number of

interactions solely because the set contains a handful of extremely highly connected proteins.

Intuitively, if our sets of DE proteins happened to contain the single most highly connected protein in

the network, a statistically significant result could arise simply because that one protein participates in

many interactions, rather than because the set of DE proteins are highly interconnected with one

another in the protein interaction network as a whole. For this reason, a reasonably large body of

literature has discussed the importance of controlling for network topology, and observed that many

apparently interesting examples of interconnectedness in protein-protein interaction networks can be

attributed solely to the overall number of interactions made by the proteins in question (e.g., Fig. 3 in

the Verleyen *et al.* reference cited in the revised manuscript).

Throughout the manuscript, we were careful to control for this confounding factor, both in our analysis

of the shotgun proteomics data in Figure 1, and in the rewiring-based functional enrichment test

described in Figure S4 (now S7). Notably, the fact that **null 2** is a much more stringent null

hypotheses can be seen by comparing the number of interactions on the x-axis (that is, under the null

hypothesis) in Figure 1F-G and Response figure 1.1 : under **null 1** we expect only ~2,000 interactions

between the proteins DE between 0h and 24 h, but under **null 2** , given their connectivity in the

literature-curated network, we expect to see ~6,500.

To clarify our motivation for the rewired network null, and the procedure used to obtain the null

distribution, we have revised the paragraph in question as follows:

Our attention was drawn to the enrichment for GO terms related to protein-protein interactions after 24 h

of IFN stimulation, including “protein binding,” “protein heterodimerization,” and “protein

heterotetramerization.” We therefore sought to determine whether proteins that were differentially

expressed at 24 h, in comparison to cells stimulated with IFN for 4 h or to unstimulated cells, displayed a

statistically significant tendency to physically interact. To test this hypothesis, we compared the observed

number of protein-protein interactions between differentially expressed proteins in each of the two

comparisons, using the InBio Map database [22] , to the number of interactions observed in networks

rewired using degree-preserving randomization [23]. Importantly, unlike a null model in which an

equivalent number of proteins are drawn at random from the network, this rewiring-based null model

controls for artifactual differences stemming from the degree distribution of the network, whereby a

statistically significant result can reflect the high connectivity of the differentially exposed proteins across

the entire network as opposed to their selective interconnectivity with one another [24, 25]. This analysis

revealed a substantial excess of physical protein-protein interactions between differentially expressed

proteins , relative to random expectation (Figure 1F–G). We compared the strength of this enrichment to

that observed for 3,263 curated gene expression signatures of disparate biological and clinical states

catalogued in the MSigDB “chemical and genetic perturbations” (CGP) collection. Remarkably, proteins

differentially expressed in the 24 h vs. 4 h and 24 h vs. unstimulated comparisons displayed a greater

enrichment for physical interactions than 95% and 97% of CGP signatures, respectively (Figure 1H).

Together, these results led us to hypothesize that, in addition to its effects on ISG transcription, IFN

stimulation induces rewiring of cellular protein-protein interaction networks.

Finally, we additionally performed the analysis of gene signatures from the MSigDB “chemical and

genetic perturbations” collection that was suggested by the reviewer. Notably, many of these gene

signatures represent curated sets of genes involved in a coordinated biological program that one

would strongly expect *a priori* to be similarly enriched for physical interactions: for example, the single

most enriched gene set comprises genes involved in oxidative phosphorylation

( https://www.gsea-msigdb.org/gsea/msigdb/geneset_page.jsp?geneSetName=MOOTHA_VOXPHOS )

. Nonetheless, we found that the proteins differentially expressed in response to IFN in our SILAC are

more strongly enriched for physical interactions with one another than ≥95% of CGP signatures,

reflecting the marked tendency of these proteins to interact physically with one another. We have

added a new panel to Fig. 1 showing this analysis:

**Figure 1H.** Enrichment for protein-protein interactions between 3,263 curated gene signatures from the

MSigDB chemical and genetic perturbations collection, histogram, and the sets of proteins differentially

expressed after 24 h of IFN stimulation relative to unstimulated cells or cells stimulated with IFN for 4 h,

dotted lines.

2. Figure 2D. Adding secondary X axis with the approximate molecular weight would be beneficial.

Per the reviewer’s suggestion, we have added a secondary x-axis showing the approximate molecular

weight:

We have revised the Methods section to comment on how this was determined:

Samples were collected into 80 fractions by a 1200 Series analytical HPLC (Agilent Technologies) at a

flow rate of 0.5 mL/min at 8°C. To avoid aggregated proteins and small monomers, only fractions 6-65,

corresponding to molecular weights ~2 mDa - 65 kDa as approximated by the use of common standards

thyroglobulin, apoferritin, and bovine serum albumin (Sigma-Aldrich), were submitted for LC-MS/MS

analysis and utilized for PCP.

3. Page 6. L12. The text refers to 2590 protein groups identified, whereas Figure 2D mentions 1520

as the "complete set" of PCP profiles. What is the reason for this discrepancy?

We apologize for this oversight: the set of 1,520 profiles is the number of proteins whose

chromatograms passed the quality control filtering implemented in the PrInCE package. The figure

legend has been revised to clarify this point:

(D) Complete set of PCP chromatograms passing quality control in PrInCE (n = 1,520) defined by

heavy/light ratio, arranged by index of maximum protein abundance, from a representative biological

replicate.

4. Methods. Page 23 L19. The authors integrate quite a number of protein interaction databases.

However, InnateDB (https://www.innatedb.com/), which would be particularly relevant for estimating

the number of novel IFN-specific interactions is not included.

As noted and described more fully above in response to major point #4, we include an analysis of

overlap with InnateDB in the revised manuscript.

5. Page 23. L60 Shawl et al (2017) Reference number missing (should be [7]).

Fixed, thank you.

6. Page 29. Figure 5A. Is "Control" the same as "Unstimulated"? If so, it should be called so for

consistency. Also, the colors should be used consistently (Figure 3I uses reversed palette)

Thank you for pointing this out. We have adjusted the colour palette to be consistent throughout the

manuscript.

7. Page 29. Figure 5D. Does the middle panel show the same data as the lower one, just without

STAT1 profile? Is so, perhaps it should be removed to avoid confusion. Are the shown profiles the

average of 3 replicates?

We have removed the middle panel as requested, and clarified that these plots show data from a

representative PCP replicate.

8. Page 31. Figure 6E Legend. "yea"?

Fixed, thank you.

9. Page 34. Figure S2: Y axis should read: "Protein quantifications (thousands)". Using Log10 scale

for Y axis would make the plot more informative. What does the dashed line denote?

The y-axis title and scale of Figure S2A have been modified as requested by the reviewer, and the

figure legend revised to clarify that the dashed lines and shaded areas represent z score thresholds

corresponding to a two-tailed family-wise error rate of 0.05:

**Figure S2:** (A) Distribution of z scores for 245,841 total protein quantifications across three biological

replicates, as determined by MODERN (Methods). Dashed lines and shaded areas show z score

thresholds corresponding to a two-tailed family-wise error rate of 0.05 in each replicate, given the total

number of points observed.

10. Page 35. Figures S3B-S3D: It's not clear which method was used to obtain the trend line and

confidence intervals. The marginal density plots suggests that the distributions are not normal

(including the autocorrelation z-score). In such cases applying linear regression is problematic, since

the results could be biased by the outliers. The authors may consider transforming the metrics (e.g. by

log-transform), so that the distribution of the transformed data is more appropriate for linear

regression.

To address the possibility raised by the reviewer that the results presented in Figures S3B-D (now

S6B-D) are biased by a handful of outliers, we performed three analyses.

First, we compared the Spearman correlation (a nonparametric, rank-based method) and ordinary

least squares linear regression, which as the reviewer notes may be biased by outliers, between the

autocorrelation z score and the three different evolutionary outcomes. We consistently observed

statistically significant associations using either method. This suggests that the associations are not

being driven by a small number of outliers. We include a new panel in this figure in the revised

manuscript, Figure S6E, showing this analysis (included below).

Second, in order to visually depict the relationships between autocorrelation and the three

evolutionary outcomes in a more accurate and flexible manner, we have replaced the linear

regression trend lines in these figures with local polynomial regression (loess; see below).

Third, as detailed above in the response to reviewer #1, we also considered the possibility that our

results might be driven by outliers at the SEC chromatogram level, which could result in artifactually

large autocorrelation z scores for a subset of proteins. To address this possibility, we repeatedly

sampled with replacement from the raw chromatograms, then re-did the autocorrelation analysis,

effectively allowing us to sample autocorrelation values from the empirical distribution of the observed

PCP data. If the rank correlation observed over the entire dataset was an artefact of a relatively small

number of unreliable data points in the raw chromatograms, then we might expect that the Spearman

correlation in bootstrapped PCP datasets would frequently change sign (in other words, we would

sometimes observe a positive correlation instead of a negative one). However, this was not the case.

We include the distribution of Spearman correlation values for dN/dS, phylogenetic breadth, and pLI in

the revised Figure S6B-D:

**Figure S 6 . Evolutionary plasticity of the interactome response to IFN stimulation.**

(A) Distribution of autocorrelation z scores between IFN-stimulated and unstimulated protein correlation

profiles, derived from Stouffer integration across all three replicates. Selected highly rewired proteins are

highlighted.

(B) Top, relationship between autocorrelation z score and ratio of nonsynonymous to synonymous

substitutions (dN/dS; (Spearman ρ = –0.11, p = 1.9 × 10 –4 ). Bottom, distribution of Spearman correlation

coefficients when sampling with replacement from SEC-PCP-SILAC chromatograms and re-calculating

the autocorrelation z score between IFN-stimulated and unstimulated protein correlation profiles.

(C) Top, relationship between autocorrelation z score and proportion of genomes in the InParanoid

database [50] in which an ortholog of the protein was present ρ = 0.14, p = 5.3 × 10 –7 ). Bottom, distribution

of Spearman correlation coefficients when sampling with replacement from SEC-PCP-SILAC

chromatograms and re-calculating the autocorrelation z score between IFN-stimulated and unstimulated

protein correlation profiles.

(D) Top, relationship between autocorrelation z score and pLI score quantile (Spearman ρ = 0.18, p = 5.9

× 10 –5 ). Bottom, distribution of Spearman correlation coefficients when sampling with replacement from

SEC-PCP-SILAC chromatograms and re-calculating the autocorrelation z score between IFN-stimulated

and unstimulated protein correlation profiles.

(E) Spearman correlation coefficients, left, and linear regression coefficients, right, between the

autocorrelation z score and ratio of nonsynonymous to synonymous substitutions (dN/dS), the proportion

of genomes in the InParanoid database in which an ortholog of the protein was present (phylogenetic

breadth), and the pLI score quantile (pLI).

Collectively, these results indicate that, although the magnitudes of the observed associations are

relatively modest, they consistently remain statistically significant under a number of different analysis

paradigms. We have revised the relevant paragraph of the manuscript to emphasize the modest

magnitude of the associations and refer to the additional analyses presented in the supplementary

figure as follows:

Comparing the IFN-induced autocorrelation of each protein to its evolutionary rate, as quantified by the

ratio of nonsynonymous to synonymous substitutions (dN/dS), revealed a modest, but statistically

significant negative correlation (Spearman’s ρ = –0.11, p = 1.9 × 10 –4 ; Figure S 6 B). Similar ly modest but

significant associations conclusions were observed in comparisons to the total number of species in which

an ortholog of a given gene was present (ρ = 0.14, p = 5.3 × 10 –7 ; Figure S 6 C) [44] , or to the pLI score

[45] , a measure of mutational constraint derived from large-scale human exome sequencing (ρ = 0.18, p =

5.9 × 10 –5 ; Figure S 6 D). Moreover, these associations remained consistent when controlling for changes

in protein abundance observed in the shotgun proteomics data using the partial Spearman correlation (p ≤

2.6 × 10 –4 ), when analyzing the data using linear regression (p ≤ 6.2 10 –3 ; Figure S6E), and when

sampling with replacement from the underlying SEC-PCP-SILAC chromatograms (Figure S6B-D).

Collectively, these results indicate that rapidly evolving proteins are disproportionately rewired in the

protein-protein interaction network by IFN signalling, to a degree that cannot be explained by changes in

protein abundance alone, suggesting that species-specific differences in the innate immune response may

be mediated in part through the effects of protein sequence divergence on protein-protein interactions.

11. References to the "modern" method (e.g. Figure S2A, S2C legend). The term "modern method" is

confusing as it might be not clear that it refers to the new version of PrInCE package. Perhaps the

authors may consider using the more specific name for the method or, at least, using capital letter and

quotes ("Modern" method).

We agree that this could be confusing to readers, and have revised the manuscript to refer to

MODERN using all-caps throughout, emphasizing the fact that this is an acronym for the specific

piece of software.

12. Table S2: It would be nice to have a column listing the names or accession codes of the enriched

genes. E.g. 17 genes from the "viral entry into the host cell" are upregulated at 4h, so it would be nice

to know their identities.

A column containing the names of all genes associated with each GO term has been added to Table

S2. In doing so, we opted to take advantage of updated versions of the Gene Ontology that have

been released since our original analysis, leading to minor changes to Figures 1D-E and S1B.

13. Table S4: Column with the names of enriched genes would be helpful as well as the column with

ontology (BP/MF/CC).

We have updated this supplementary table (Table S6 in the revised manuscript) to include the branch

of the GO (BP/MF/CC) each term originates from as well as the identities of all the interactions within

each GO term.
